# Chromosome-level reference genome of the jellyfish *Rhopilema esculentum*

**DOI:** 10.1093/gigascience/giaa036

**Published:** 2020-04-21

**Authors:** Yunfeng Li, Lei Gao, Yongjia Pan, Meilin Tian, Yulong Li, Chongbo He, Ying Dong, Yamin Sun, Zunchun Zhou

**Affiliations:** 1 Liaoning Ocean and Fisheries Science Research Institute, 50 Heishijiao St., Dalian, Liaoning 116023, China; 2 Tianjin Biochip Corporation, 23 Hongda St., Tianjin 300457, China

**Keywords:** jellyfish, *Rhopilema esculentum*, whole-genome sequencing, chromosome-level assembly, toxin-like genes

## Abstract

**Background:**

Jellyfish belong to the phylum Cnidaria, which occupies an important phylogenetic location in the early-branching Metazoa lineages. The jellyfish *Rhopilema esculentum* is an important fishery resource in China. However, the genome resource of *R. esculentum* has not been reported to date.

**Findings:**

In this study, we constructed a chromosome-level genome assembly of *R. esculentum* using Pacific Biosciences, Illumina, and Hi-C sequencing technologies. The final genome assembly was ∼275.42 Mb, with a contig N50 length of 1.13 Mb. Using Hi-C technology to identify the contacts among contigs, 260.17 Mb (94.46%) of the assembled genome were anchored onto 21 pseudochromosomes with a scaffold N50 of 12.97 Mb. We identified 17,219 protein-coding genes, with an average CDS length of 1,575 bp. The genome-wide phylogenetic analysis indicated that *R. esculentum* might have evolved more slowly than the other scyphozoan species used in this study. In addition, 127 toxin-like genes were identified, and 1 toxin-related “hub” was found by a genomic survey.

**Conclusions:**

We have generated a chromosome-level genome assembly of *R. esculentum* that could provide a valuable genomic background for studying the biology and pharmacology of jellyfish, as well as the evolutionary history of Cnidaria.

## Data Description

### Background

Jellyfish belong to the phylum Cnidaria, which occupies an important phylogenetic location and is one of the earliest branching Metazoa lineages [1]. The jellyfish *Rhopilema esculentum* (Kishinouye, 1891), an edible species in the class Scyphozoa (also named "true jellyfish"), is widely distributed in the seas around China, Japan, and Korea [2], and it is one of the most abundant fishery animals in these locations. *R. esculentum* has been exploited as food for thousands of years and has been gaining more attention recently because of its pharmacological properties [3]. In contrast to many other jellyfish species that have drawn public attention because of their harmful blooms [4], the population of *R. esculentum* has declined in recent years as a result of overfishing [2]. The stock enhancement and aquaculture of *R. esculentum* have been initiated to meet the expanding market demand, which accounts for ∼82,280 tons per year, generating US $122,800,000 worth of profit per year for the Chinese economy [5]. The lack of genomic resource has limited the phylogenetic study of jellyfish and the investigation of their many specific characteristics. Recently, several genome assemblies have been reported for the medusozoan species, including the moon jellyfish (*Aurelia aurita*) [6, 7], the giant Nomura's jellyfish (*Nemopilema nomurai*) [8], the upside-down jellyfish (*Cassiopea xamachana*) [9], the hydrozoan jellyfish *Clytia hemisphaerica* [10], *Morbakka virulenta* [7], *Alatina alata* [9], and *Calvadosia cruxmelitensis* [9]. However, no chromosome-level reference genome has been reported for the class Scyphozoa, and at present, there is very limited information about the genome architecture of *R. esculentum*. In the present study, we sequenced the chromosome-level genome of *R. esculentum* and assembled and annotated it to improve our understanding of the evolutionary and pharmacology characteristics of jellyfish.

### Sample and sequencing

**Figure 1:**
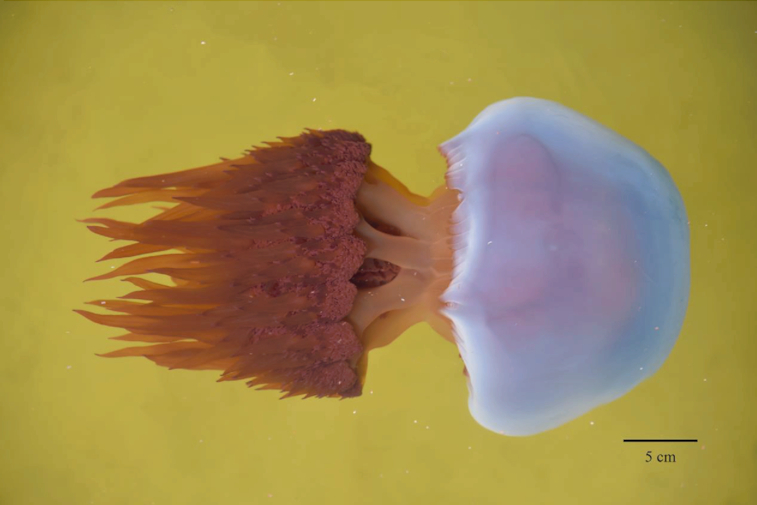
Picture of the jellyfish *R. esculentum* captured in Yingkou, Liaoning Province, China.

One cultured *R. esculentum* (NCBI:txid499914) individual was collected from Yingkou, Liaoning Province, China (Fig. 1). After starving for 2 days, the epidermis tissue was sampled, and genomic DNA was extracted using a TIANamp Marine Animal DNA Kit (Tiangen, Beijing, China) and then directly used for the genomic DNA sequencing. The genomic DNA was sheared using a sonication device, and the resulting fragments were used for the construction of short-insert paired-end (PE) libraries. Short-insert libraries with a size of 500 bp were constructed in accordance with the instructions in the Illumina library preparation kit. All libraries were sequenced on an Illumina HiSeq2500 platform (Illumina, San Diego, CA, USA) with 150-bp PE. In total, ∼22.6 Gb (80×) of raw data were generated, and 20.03 Gb (71×) of clean data were filtered by FastQC (FastQC, RRID:SCR_014583) v0.11.2 (Supplementary Table S1). The genomic DNA used for sequencing was also sheared to yield ∼20 kb fragments for the construction of Pacific Biosciences (PacBio) libraries. DNA fragments of <7 kb were filtered using BluePippin (Sage Science, Beverly, MA, USA). The filtered DNA was then converted into the proprietary SMRTbell library using the PacBio DNA Template Preparation Kit. In total, 39.76 Gb (140×) of quality-filtered data with a mean length of 7196 bp were obtained from the PacBio Sequel platform (Supplementary Table S1).

### Genome size and heterozygosity estimation

The distribution of *k*-mer frequency, also known as the *k*-mer spectrum, is widely used for the estimation of genome size. We used a jellyfish software based on a *k*-mer distribution [11] to estimate the genome size with high-quality reads >Q20 from short-insert libraries (500 bp). We obtained a *k*-mer (*k* = 17) depth distribution from the jellyfish analysis and clearly observed the peak depth from the distribution data. We obtained a genome size estimation of 290 Mb and a heterozygosity of 1.68% by GenomeScope v1.0.0 (Supplementary Fig. S1) [12]. A total of 54.4% of the genome was predicted to be non-repetitive sequences.

### Genome assembly and annotation

In the present study, the long reads of PacBio sequencing data were used to solve the high level of heterozygosity, which is one of the main challenges in the assembly of marine invertebrate genomes [13, 14]. The genome assembly was performed using the software wtdbg2 with default parameters [15]. The assembly sequences were then polished using Quiver (SMRT Analysis v2.3.0) with default parameters. To achieve higher continuity and accuracy for the assembled genome, 5 rounds of iterative error correction were performed with the Illumina clean genome data using in-house script. Finally, a genome of 275.42 Mb was assembled, with 760 contigs and a contig N50 size of 1.13 Mb (Table 1 and Supplementary Fig. S2).

**Table 1:** Statistics of the assembly and annotation of *R. esculentum* genome

Genome feature	Value
**Genome assembly**	
Total length (Mb)	275.42
Contig N50 (Mb)	1.13
Longest contig (Mb)	6.59
Contig number	760
GC content (%)	36.25
Pseudochromosome number	21
Scaffold N50 (Mb)	12.97
**Genome annotation**	
Gene number	17,219
Gene density (per 100 kb)	62.52
CDS mean length (bp)	1,575
Exon mean length (bp)	198.8
Intron mean length (bp)	987.2
Exon number per gene	7.92
Exon GC content (%)	42.29

Both RepeatModeler (RepeatModeler, RRID:SCR_015027) and RepeatMasker (RepeatMasker, RRID:SCR_012954) [16] were used to perform the *de novo* identification and masking of repeat sequences. To ensure the integrity of the genes in subsequent analysis, all repeat sequences, except for the low-complexity or simple repeats, were masked in this analysis because some of the low-complexity or simple repeats could be found in the genes. Finally, 29.23% of the assembled bases (80,495,815 bp) were masked (Supplementary Table S2). Of these, 9.93% could be annotated with known repeat families, and 19.30% were unclassified repeats.

The identification of protein-coding regions and the prediction of genes were performed using a combination of *ab initio* prediction, homology-based prediction, and transcriptome-based prediction methods. The *ab initio* gene prediction was conducted with Augustus (Augustus: Gene Prediction, RRID:SCR_008417) version 2.5.5 [17], GlimmerHMM (GlimmerHMM, RRID:SCR_002654) version 3.0.1 [18], and SNAP15 [19] to predict the coding genes. For the homology-based prediction, homologous proteins of several Cnidarian species (myxosporean [*Thelohanellus kitauei*], coral [*Stylophora pistillata* and *Orbicella faveolata*], hydrozoan [*Hydra vulgaris*], sea anemone [*Exaiptasia pallida*], and the Cnidaria EST database) were downloaded from NCBI and aligned with our assembled genome. Then, GeneWise (GeneWise, RRID:SCR_015054) version 2.2.0 [20, 21] was used to generate the gene structures based on the homology alignments. For transcriptome-based prediction, 60 individuals of 4 development periods (scyphistoma, strobili, ephyra, and juvenile medusa) were collected. Five individuals were pooled and 3 replicates were set for each development period analysis. The transcriptome of samples were sequenced using the Illumina HiSeq2500 platform (154.6 Gb clean reads, PE-250) (Supplementary Table S3) and the resulting sequences were mapped to the genome assembly using TopHat (TopHat, RRID:SCR_013035) version 2.0.8 [22]. Cufflinks (Cufflinks, RRID:SCR_014597) version 2.1.1 [23, 24] was then used to identify the spliced transcripts in the gene models. All the gene evidence predicted from the above 3 approaches were integrated by EvidenceModeler (EVM) [25] into a weighted and non-redundant consensus of the gene structures. A total of 17,219 genes, with an average CDS length of 1,575 bp, were finally predicted to be present in the genome of *R. esculentum* (Table 1). All the gene sequences were searched using BLASTP with an *E*-value of 1e^−5^ against several public databases, including NR [26], GO (Supplementary Fig. S3) [27], Swiss [28], KOG (Supplementary Fig. S4) [29], and KEGG [30], to obtain the functional annotation. A total of 16,713 genes (97.1%) were successfully mapped to ≥1 database, and 8,880 genes were annotated in all 4 databases (*E-*value < 1e^−5^) (Supplementary Fig. S5).

### Quality assessment

We first aligned all the Illumina genome reads against the *R. esculentum* assembled genome using BWA (BWA, RRID:SCR_010910), version 0.7.17, to evaluate the coverage of the genome. The percentage of aligned reads was estimated to be 99.81%. BUSCO (BUSCO, RRID:SCR_015008), version 3.0.2 [31], was then used to evaluate the integrity of the genome (Supplementary Table S4). The values of core gene estimation were calculated as follows: C: 97.0% (S: 92.1%, D: 5.0%), F: 1.7%, M: 1.3%, n: 303, where C, S, D, F, M, and n indicate complete BUSCOs, complete and single-copy BUSCOs, complete and duplicated BUSCOs, fragmented BUSCOs, missing BUSCOs, and total BUSCO groups searched, respectively (Supplementary Table S5). The results indicated that the assembly covered most of the genetic regions, further confirming the assembly quality of the *R. esculentum* genome.

### Pseudochromosome construction

**Figure 2:**
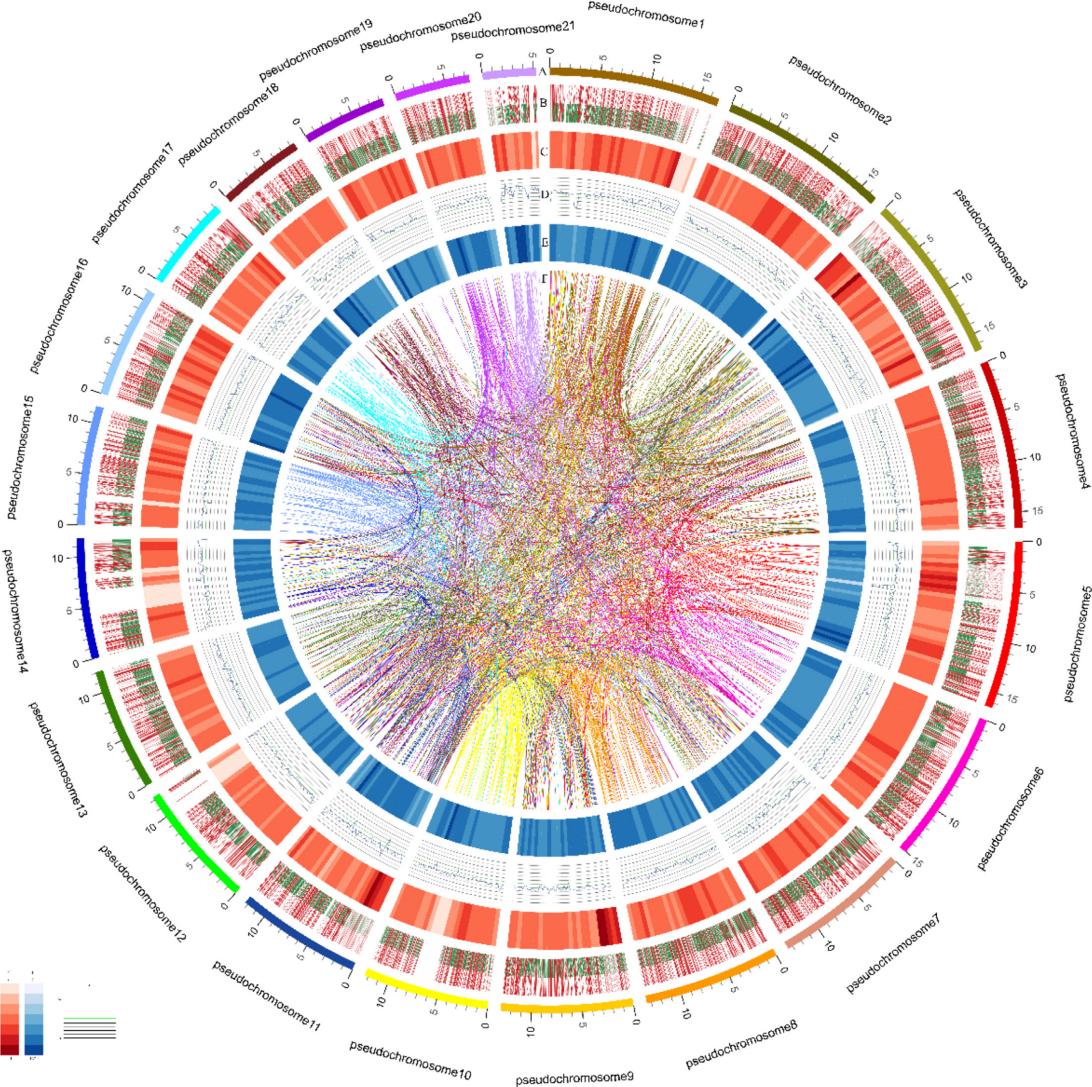
Schematic representation of the genomic characteristics of *R. esculentum*. Track A: 21 pseudochromosomes of *R. esculentum* genome (Mb). Track B: Protein-coding genes present in the scaffolds. Red represents genes on forward strand, and green, genes on reverse strand. Track C: Distribution of gene density with sliding windows of 1 Mb. Higher density is shown in darker red color. Track D: Distribution of GC content in the genome. Track E: Distribution of repeats in the genome. Track F: Schematic presentation of major interchromosomal relationships.

Hi-C experiments were used for the chromosome assembly of *R. esculentum*. The whole-body homogenate of 1 *R. esculentum* was fixed in 1% (vol/vol) formaldehyde and was then used to prepare the Hi-C libraries. Nuclei extraction and permeabilization, chromatin digestion, and proximity-ligation treatments were performed as previously described [32]. The DNA was digested overnight (12 h) with 200 U of the restriction enzyme *MboI*at 37°C with shaking. The libraries were sequenced on the Illumina X-TEN platform (San Diego, CA, USA) with 2 × 150 bp reads. They were independently analysed in the HiC-Pro pipeline (default parameters and LIGATION_SITE = GATC) [33]. A total of 23.96 Gb of trimmed reads were obtained, accounting for ∼82-fold coverage of the *R. esculentum* genome. The 3D-DNA was used to assign the order and orientation of each group [34]. The contact maps were plotted using HiCPlotter software [35]. Finally, 260.17 Mb (94.46%) of the assembly was anchored onto 21 pseudochromosomes, which was in agreement with the karyotype (2n = 42) of *R. esculentum* [36] (Fig. 2, Supplementary Fig. S6, and Supplementary Table S6). This chromosome-level assembly resulted in a scaffold N50 of 12.97 Mb.

### Phylogenetic analysis

To examine the evolutionary relationships among *R. esculentum* and other species, the whole protein sequences of *R. esculentum* and 12 other species (Supplementary Table S7) were analysed, including species from Ctenophora (ctenophore [*Mnemiopsis leidyi*]), Porifera (demosponge [*Amphimedon queenslandica*]), Placozoa (*Trichoplax adhaerens*), Cnidaria (jellyfish [*R. esculentum* and *A. aurita*], Hydrozoa (*H. vulgaris*), coral (*S. pistillata*), sea anemone (*Nematostella vectensis*), Protostomia (Lophotrochozoa [Pacific oyster (*Crassostrea gigas*)], Ecdysozoa [cladoceran (*Daphnia pulex*)]), and Deuterostomia (Echinodermata [sea urchin (*Strongylocentrotus purpuratus*)], Hemichordata [acorn worm (*Saccoglossus kowalevskii*)], Chordata [zebrafish (*Danio rerio*)]). All protein models of the 12 other species were obtained from Ensembl or NCBI. Orthologous alignment analysis was performed using OrthoMCL (OrthoMCL DB: Ortholog Groups of Protein Sequences, RRID:SCR_007839) [37]. In detail, the protein-coding genes from the above-sequenced genomes were aligned with each other using the BLASTP program [38]. Similarity in the pair-wise sequence alignments generated by BLASTP was used as distance parameters for gene family clustering by MCL with an inflation value of 1.5.

**Figure 3:**
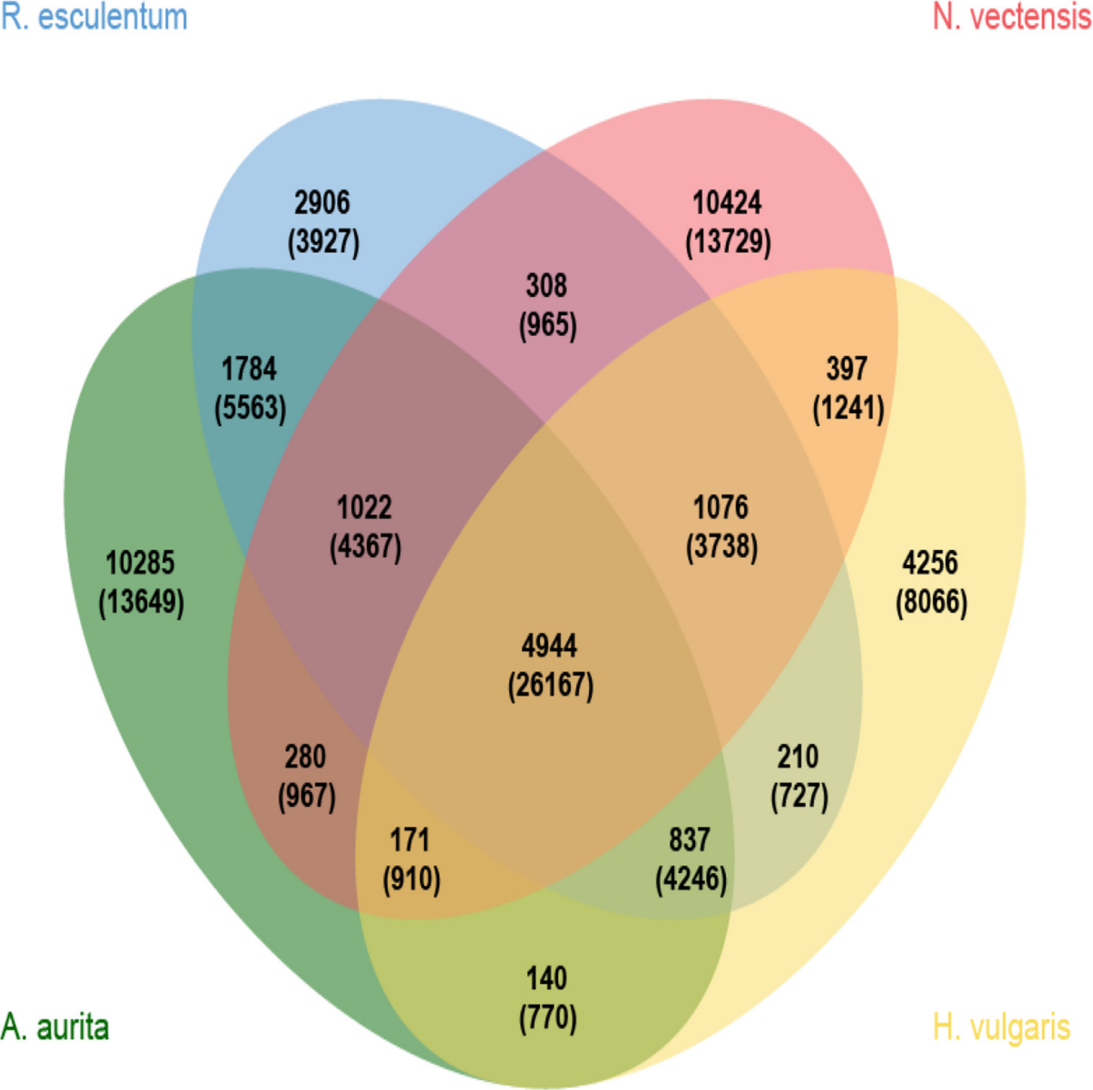
Venn diagram of the protein-coding orthologues shared among *R. esculentum, H. vulgaris, N. vectensis*, and *A. aurita*. Each number represents the number of gene families, and the number in parentheses is the number of genes.

**Figure 4:**
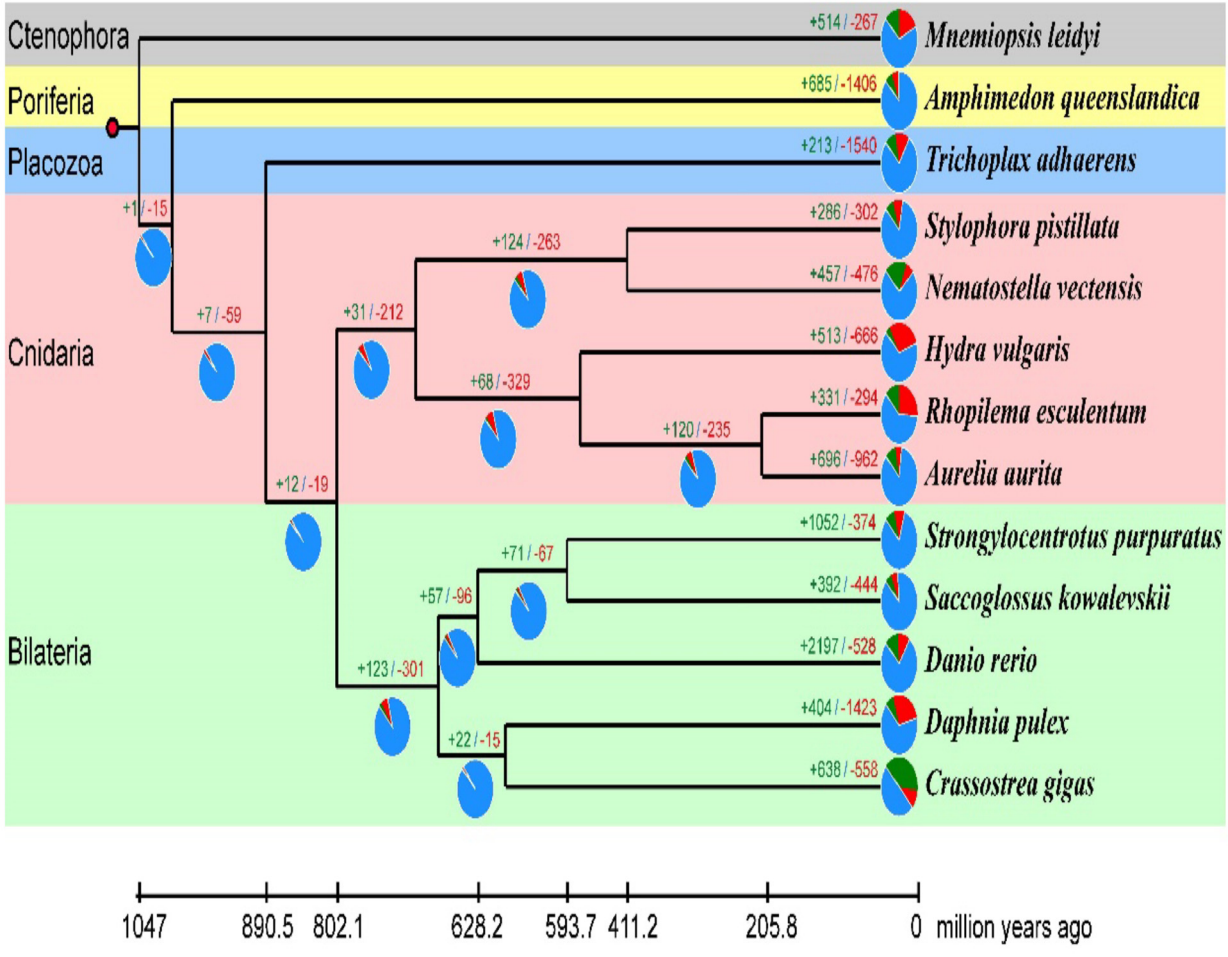
Phylogenetic analysis of *R. esculentum* and other metazoan species. The numbers of gene gains (plus sign) and gene losses (minus sign) are shown on the branches, which are also displayed as pie plots (green: gene gain; red: gene loss; blue: gene persistence). The divergence times are dated and displayed below the phylogenetic tree.

A set of 32,138 gene families were eventually identified among the other 12 species, of which 2,092 families were present in all 13 species (Fig. 3 and Supplementary Table S8). A total of 335 selected single-copy orthologous genes were aligned using MUSCLE (MUSCLE, RRID:SCR_011812) v3.6 [39] and then concatenated into a single multiple sequence alignment through an in-house Perl script. A maximum likelihood phylogeny was reconstructed using RAxML (RAxML, RRID:SCR_006086) [40] (Fig. 4). The phylogenetic results supported the monophyly of *R. esculentum, A. aurita*, and *H. vulgaris*. The PROTGAMMAJTT model was used for RaXML analyses [40]. The divergence times of *M. leidyi*vs *A. aurita, S. purpuratus*vs *A. aurita*, and *D. rerio*vs *N. vectensis* were retrieved from the time tree [41] and used as the fossil calibration. R8s was used to calculate the divergence time of each node in the phylogenetic tree [42]. We dated the divergence time of *R. esculentum* and *H. vulgaris* to ∼501.71 million years ago, consistent with previous studies [43]. To compare the jellyfish genomic traits with those of the other 12 species, we performed a comparative genomic analysis for all 13 species using CAFE software (Supplementary Table S9) [44]. Twenty-seven gene families were found to be significantly expanded and another 27 gene families were found to be significantly contracted in *R. esculentum* (*P* < 0.05) (Supplementary Tables S10 and S11). Interestingly, the gene families enriched in the GO category of transmembrane transport were significantly expanded, and the relative GO sub-categories included drug transmembrane transport, drug transmembrane transporter activity, ion transmembrane transporter activity, and amino acid transmembrane transporter activity. The action of venom, an important characteristic of jellyfish species, may contribute to gene expansion in the transmembrane transport category [45, 46].

A comparative genomic analysis was performed for the 4 jellyfish species in the class Scyphozoa (including *R. esculentum, A. aurita, N. nomurai*, and *C. xamachana*) and *H. vulgaris* (used as outgroup, and to calculate the divergence time). A total of 244 unique gene families were identified in *R. esculentum* using BLASTP with an *E-*value of 1e^−5^ in the NR database. It was surprising that more than half of those (136 unique gene families) were best annotated with Anthozoa species in the NR database. It was suggested that the 136 unique gene families were not from the split of *R. esculentum* but from the ancestor of Anthozoa and Scyphozoa. This result implied that some gene families that were possessed by the last common ancestor of Anthozoans and Scyphozoans were kept by the Anthozoan species and *R. esculentum* but were lost in *A. aurita, N. nomurai, C. xamachana*, and *H. vulgaris*. This was also supported by the phylogenetic analysis of the 13 species, in which *R. esculentum* was found to exhibit fewer gene gains (331) and fewer gene losses (294) compared with *H. vulgaris* (513 gains and 666 losses) and *A. aurita* (696 gains and 962 losses) (Fig. 4). This indicated that *R. esculentum* might have evolved more slowly than the other scyphozoan species used in this study.

### Analysis of toxin-like genes in jellyfish

Jellyfish is one important lineage of extant venomous animals [47, 48]. The venom is injected into the victim or prey when triggered to discharge. Jellyfish stings are dangerous to swimmers and fishermen because they can cause local oedema, vesicular eruption, shock, and even death [49, 50]. The venom of jellyfish consists of polypeptides, enzymes, and some non-protein bioactive components [48], such as neurotoxins, myotoxins, hemolytic toxins, and cardiotoxins [51]. The venom constituents of jellyfish have been investigated by pharmacological studies in recent years. Omics analyses, especially transcriptomic and proteomic analyses, have been used to conduct large-scale identification of toxins and related genes from jellyfish, and many putative toxins have been identified [47, 50–53]. However, owing to the limitation of genome information and sampling [50], the overall understanding of toxin-like genes is limited, which may be responsible for the lack of consistency among the results obtained from previous studies [53]. Here, we conducted a genomic survey of toxin-like genes in the assembled *R. esculentum* genome.

In step 1, all the genes of *R. esculentum* were screened using BLASTP with a cutoff *E*-value of 1e^−10^ against the database of the animal toxin annotation project (Tox-Prot) in UniProt. In step 2, according to the best hits of gene annotations of NR, Uniprot, and Tox-Prot, the genes that were consistently annotated as toxin-like genes were then chosen. In step 3, to make the pool of venom-related genes more complete, we checked all the gene annotations of the jellyfish and picked out the genes where the annotations were consistent with the annotations in the database of Tox-Prot and were not identified in the first 2 steps. These genes were also considered as toxin-like genes.

There were 127 toxin-like genes identified, including 60 metalloproteinases, 18 phospholipases, 13 nucleases and nucleotidases, 13 peptidases and inhibitors, 12 genes with toxin activity, and 11 other venom-related genes (Table 2). It is not surprising that metalloproteases were the most abundant group of toxins because they are widely considered to be a key toxic component in various venomous animals, such as spiders [54], snakes [55], scorpions [56], and jellyfish [50, 57]. Metalloprotease can interfere with blood coagulation and induce necrosis. Metalloprotease is always associated with the symptoms of stings, such as swelling, myonecrosis, inflammation, and blister formation [48, 53].

**Table 2:** Summary of all the identified toxin-like genes from the genome of the jellyfish *R. esculentum*

Gene	Copy No.	Description	Family	Reported in jellyfish
Phospholipase A2	9	Phospholipase A2 activity	Phospholipase A2 family	Yes
Acidic phospholipase A2 PA4	4	Phospholipase A2 activity	Phospholipase A2 family	Yes
Phospholipase A2 isozymes PA3A/PA3B/PA5	4	Phospholipase A2 activity	Phospholipase A2 family	Yes
Putative phospholipase B-like 2	1	Hydrolase activity	Phospholipase B-like family	Yes
Zinc metalloproteinase nas	39	Metalloendopeptidase activity		Yes
Disintegrin and metalloproteinase	21	Metalloendopeptidase activity		Yes
Ectonucleotide pyrophosphatase/phosphodiesterase	8	Nuclease activity	Nucleotide pyrophosphatase/phosphodiesterase family	Yes
5′-Nucleotidase	5	5′-Nucleotidase activity	5′-Nucleotidase family	Yes
Serine carboxypeptidase	1	Serine-type carboxypeptidase activity	Peptidase S10 family	Yes
Serine protease	7	Serine-type endopeptidase activity	Peptidase S1 family	Yes
Prothrombin	2	Serine-type endopeptidase activity	Peptidase S1 family	Yes
Dipeptidyl peptidase 9	1	Serine-type peptidase activity	Peptidase S9B family	Yes
Kunitz-type serine protease inhibitor	1	Serine-type endopeptidase inhibitor activity	Venom Kunitz-type family	Yes
Cystatin	1	Cysteine-type endopeptidase inhibitor activity	Cystatin family	Yes
Plancitoxin-1	3	Toxin activity	DNase II family	Yes
Ryncolin	6	Toxin activity	Ficolin lectin family	Yes
Toxin TX	2	Toxin activity	Jellyfish toxin family	Yes
Trpa1	1	Toxin activity	(High similarity with α-latrotoxin-Lt1a)	Yes
Peroxiredoxin-4	2	Protein homodimerization activity	Peroxiredoxin family	Yes
Glutaminyl-peptide cyclotransferase-like protein	1	Glutaminyl-peptide cyclotransferase activity	Glutaminyl-peptide cyclotransferase family	Yes
Lysosomal acid lipase/cholesteryl ester hydrolase	1	Lipase activity	Lipase family	Yes
Trehalase	1	α-trehalase activity	Glycosyl hydrolase 37 family	Yes
Acetylcholinesterase	1	Acetylcholinesterase activity	Type-B carboxylesterase/lipase family	Yes
Lysosomal acid phosphatase	1	Acid phosphatase activity	Histidine acid phosphatase family	No
Reticulocalbin	1	Calcium ion binding	CREC family	No
Translationally controlled tumour protein homolog	1	Calcium ion binding	TCTP family	Yes
Hyaluronidase-1	2	Hyaluronan synthase activity	Glycosyl hydrolase 56 family	Yes

Full gene names are provided in Supplementary Table S12.

Phospholipases comprise the second most abundant group of toxins. Various forms of phospholipases have been identified, such as phospholipase A2, acidic phospholipase A2 PA4, phospholipase A2 isozymes PA3A/PA3B/PA5, and putative phospholipase B-like 2. Phospholipases are ubiquitous in the venom of many poisonous animals and they exhibit various degrees of toxicity, among which hemolytic activity is the most striking [51]. High levels of phospholipase A2 activity have been observed in the tentacles of scyphozoan and cubozoan species [51, 58] and are presumably involved in defence and in the capturing of prey [48]. In the present study, 9 copies of phospholipase were found in a tandem fashion located on 3 loci of the genome.

Two copies of “jellyfish toxin,” also called "cubozoan-related porins," were also found. The jellyfish toxins have been observed in high abundance in cubozoan venoms [52] and they have also been reported in other medusozoans, such as Scyphozoans [51], Hydrozoans [59], and Anthozoans [60]. They are potent and rapid-acting toxins, having both hemolytic and pore-forming activities [48, 51]. Compared with the high abundance in cubozoans, where as many as 15 isoforms of the jellyfish toxin were found in *Chironex fleckeri*, the relatively fewer copies found in scyphozoan species may be linked to the less severe stings inflicted by these species of jellyfish [52].

Two new toxins were identified: reticulocalbin and lysosomal acid phosphatase. These toxins have not been reported in jellyfish. Reticulocalbin is known to have calcium ion–binding activity. Its role in venom is still unclear, although it was speculated to play a potentially unknown role in prey incapacitation by binding with phospholipase A2 [61, 62]. Lysosomal acid phosphatase is an orthologue of venom acid phosphatase, which is an acidic heat-labile protein with carbohydrate IgE binding epitopes [63]. It is mostly found in the honeybee and has been implicated in allergic reaction [63–65]. The discovery of these toxin-coding genes in *R. esculenum* would add to a growing understanding of the composition of jellyfish venoms. When compared with the venom composition of the jellyfish *N. nomurai* (also named *Stomolophus meleagris*), a species closely related to *R. esculentum*, it was noted that 2 types of main toxins were lost in *R. esculentum*, including a serine protease inhibitor (only 1 copy found) and a potassium channel inhibitor ShK [50]. They are known to block the activities of trypsin and plasmin and to function as neurotoxins [50]. The different compositions of the venom may account for the different symptoms after the sting. For instance, *R. esculentum* sting always causes strong pruritus compared with stings of other jellyfish species [66].

Interestingly, 8 toxin-like genes were located closely on contig 521 as a “hub,” including 4 PLA2s, 2 ENPP5s, 1 TRPA1, and 1 SLC47A1 (Table 3). The functions of toxin-like genes in the hub included phospholipase A2 activity, nuclease activity, toxin activity, and toxin extrusion. In addition, according to the chromosome-level analysis, contig 747 and contig 751 were located on the 2 sides of contig 521 and contained 5 and 3 toxin-like genes, respectively. These 3 contigs were arranged in chromosome 7 (3,691,690–13,486,489 bp) as a head-to-tail tandem, forming a bigger hub. The neighbouring genes have been shown to co-express rather than express independently [67, 68]. Thus, we speculated that contig747-contig521-contig751 tandem on chromosome 7 may play important roles in the formation and function of venom in *R. esculentum*. Further studies are needed to clarify their specific functions.

**Table 3:** Structure of the toxin-related hub on contig 521

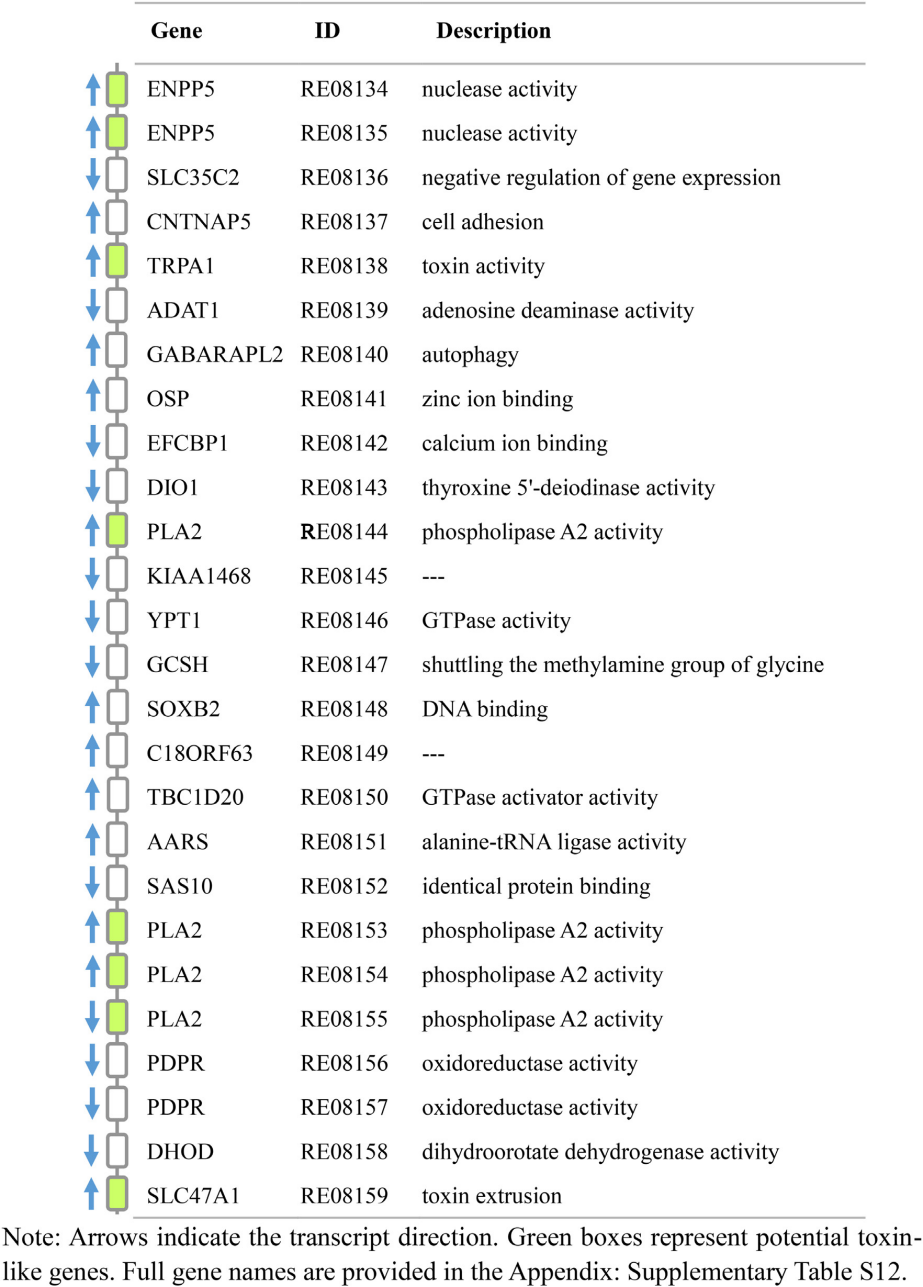

In summary, we have sequenced and assembled the genome of *R. esculentum* at the chromosome level. The obtained genome data will provide a valuable resource for conducting further study on *R. esculentum* and other Cnidarian species.

## Availability of Supporting Data and Materials

The raw genome sequencing data obtained by Illumina and PacBio platform are available via NCBI with accession Nos. SRR8617500 and SRR8617499, respectively (BioProject accession No. PRJNA523480). The raw sequencing data of the transcriptome are available via NCBI with accession No. SRR8401786-SRR8401797 (BioProject accession No. PRJNA512552). Supporting data are available via the *GigaScience* GigaDB repository [69].

## Additional Files

**Supplementary Fig. S1:** *k*-mer estimation of the genome size of *R. esculentum*.

**Supplementary Fig. S2:** Contig length distribution of the assembled genome of *R. esculentum*.

**Supplementary Fig. S3:** GO analysis and functional classification of the protein coding genes in *R. esculentum*.

**Supplementary Fig. S4:** KOG analysis and functional classification of the protein coding genes in *R. esculentum*.

**Supplementary Fig. S5:** Venn diagram of the statistics of the functional annotation.

**Supplementary Fig. S6:** Interaction frequency distribution of Hi-C links among chromosomes of *R. esculentum*.

**Supplementary Table S1:** Statistics of the clean data of Illumina and PacBio sequencing for *R. esculentum*.

**Supplementary Table S2:** Statistics of the repeat elements of *R. esculentum* genome assembly indicated by both RepeatModeler and RepeatMasker software.

**Supplementary Table S3:** Summary of the transcriptome sequenced data of *R. esculentum*.

**Supplementary Table S4:** Core gene estimation for the *R. esculentum* genome assembly obtained using BUSCO.

**Supplementary Table S5:** BUSCO scores of gene model and trinity assembly of *R. esculentum*.

**Supplementary Table S6:** Quantity of the contigs anchored with Hi-C.

**Supplementary Table S7:** Information of the 12 representative species that were used in the analysis of evolutionary relationships.

**Supplementary Table S8:** Summary of the orthologous gene clusters analysed in 13 species that were used in the analysis of evolutionary relationships.

**Supplementary Table S9:** Gene family analysis performed with CAFE.

**Supplementary Table S10:** Annotations of the significantly expanded gene families of *R. esculentum*.

**Supplementary Table S11:** Annotations of the significantly contracted gene families of *R. esculentum*.

**Supplementary Table S12:** Abbreviations and full names of the genes used in this study.

giaa036_GIGA-D-19-00354_Original_Submission

giaa036_GIGA-D-19-00354_Revision_1

giaa036_GIGA-D-19-00354_Revision_2

giaa036_Response_to_Reviewer_Comments_Original_Submission

giaa036_Response_to_Reviewer_Comments_Revision_1

giaa036_Reviewer_1_Report_Original_Submission Joseph F. Ryan -- 10/31/2019 Reviewed

giaa036_Reviewer_2_Report_Original_Submission Lucas LeclÃ¨re -- 11/10/2019 Reviewed

giaa036_Reviewer_2_Report_Revision_1 Lucas LeclÃ¨re -- 3/12/2020 Reviewed

giaa036_Reviewer_3_Report_Original_Submission David A. Gold -- 11/19/2019 Reviewed

giaa036_Supplemental_File

## Competing Interests

The authors declare that they have no competing interests.

## Abbreviations

BLAST: Basic Local Alignment Search Tool; bp: base pairs; BUSCO: Benchmarking Universal Single-Copy Orthologs; BWA: Burrows-Wheeler Aligner; CAFE: computational analysis of gene family evolution; CDS: coding domain sequence; Gb: gigabase pairs; GC: guanine-cytosine; GO: Gene Ontology; kb: kilobase pairs; KEGG: Kyoto Encyclopedia of Genes and Genomes; KOG: Eukaryotic Orthologous Groups; Mb: megabase pairs; NCBI: National Center for Biotechnology Information; NR: Non-Redundant database; PacBio: Pacific Biosciences; PE: paired-end; RAxML: Randomized Axelerated Maximum Likelihood; tRNA: transfer RNA.

## Ethics Statement

This study was approved by the Animal Care and Use Committee of Liaoning Ocean and Fisheries Science Research Institute. This study did not involve endangered or protected species.

## Authors' Contributions

Z.Z. and Yunfeng Li designed the project. M.T. and Yulong Li collected the samples. Y.P., C.H., and Y.D. extracted the genomic DNA. L.G., Y.S., and Y.P. participated in data analyses. L.G. and Y.S. wrote the manuscript. All authors have read and approved the final manuscript.

## Funding

This work was supported by the National Natural Science Foundation of China (31,302,173; 31,602,156; 31,602,155); the Science and Technology Program of Liaoning Province, China (2,013,203,001); the Natural Science Foundation of Liaoning Province, China (20,180,551,158); the Scientific Research Program of Ocean and Fisheries Administration of Liaoning Province, China (201,827); and Liaoning Science Public Welfare Research Fund Project (20,180,015).
