# Supplementary material for: Chromosome-level reference genome of the jellyfish Rhopilema esculentum
Source: Gigascience. 2020 Apr 21;9(4):giaa036. doi: 10.1093/gigascience/giaa036 (PMC7172023; doi:10.1093/gigascience/giaa036)
Supplement: giaa036_GIGA-D-19-00354_Original_Submission [file giaa036_giga-d-19-00354_original_submission.pdf]

GigaScience

Chromosome-level reference genome of the jellyfish (Rhopilema esculentum)

--Manuscript Draft--

**Manuscript Number:**	GIGA-D-19-00354
**Full Title:**	Chromosome-level reference genome of the jellyfish (Rhopilema esculentum)
**Article Type:**	Data Note
**Funding Information:**	National Natural Science Foundation of China (31302173) — Mr. Yunfeng Li<br>National Natural Science Foundation of China (31602156) — Dr. Meilin Tian<br>National Natural Science Foundation of China (31602155) — Dr. Lei Gao<br>the Science and Technology Program of Liaoning Province, China (2013203001) — Dr. Zunchun Zhou<br>the Natural Science Foundation of Liaoning Province, China (20180551158) — Mr. Yunfeng Li<br>the Scientific Research Program of Ocean and Fisheries Administration of Liaoning Province, China (201827) — Mr. Yunfeng Li<br>Liaoning Science Public Welfare Research Fund Project (20180015) — Dr. Yulong Li
**Abstract:**	Background<br><br>Jellyfish belongs to Cnidaria and it occupies an important phylogenetic location together with Porifera as one of the earliest branching Metazoa lineages after their divergence with Ctenophora. The jellyfish Rhopilema esculentum is important fishery resource in China. However, genome resource of R. esculentum has not been reported to date.<br><br>Findings<br><br>We constructed a chromosome-level genome assembly of R. esculentum using Pacific Biosciences, Illumina and Hi-C sequencing technologies. A total of 22.6 Gb and 39.76 Gb raw reads were generated using the Illumina HiSeq2500 platform and the PacBio Sequel platform, respectively. The final genome assembly was approximately 275.42 Mb, with a contig N50 length of 1.13 Mb. Using Hi-C technology to identify the contacts among contigs, 260.17 Mb (94.46%) of the assemblies were anchored onto 21 pseudochromosomes with a scaffold N50 of 12.97 Mb. We identified 17,219 protein-coding genes, with an average CDS length of 1,575 bp. The genome-wide phylogenetic analyses indicated that R . esculentum was less evolved than other species in Scyphozoa. In addition, 127 toxin-like genes were identified, and one toxin-related “hub” was found by genomic survey.<br><br>Conclusions<br><br>We first finished chromosome-level genome assembly of R . esculentum , which will provide a valuable genomic background for the studies of the jellyfish biology, the evolutionary history of Cnidaria and the pharmacology of jellyfish.
**Corresponding Author:**	Zunchun Zhou<br><br>CHINA
**Corresponding Author Secondary Information:**	

Corresponding Author's Institution:	
Corresponding Author's Secondary Institution:	
First Author:	Yunfeng Li
First Author Secondary Information:	
Order of Authors:	Yunfeng Li
	Lei Gao
	Yongjia Pan
	Meilin Tian
	Yulong Li
	Chongbo He
	Ying Dong
	Yamin Sun
	Zunchun Zhou
Order of Authors Secondary Information:	
Additional Information:	
**Question**	**Response**
Are you submitting this manuscript to a special series or article collection?	No
**Experimental design and statistics**<br><br>Full details of the experimental design and statistical methods used should be given in the Methods section, as detailed in our Minimum Standards Reporting Checklist. Information essential to interpreting the data presented should be made available in the figure legends.<br><br>Have you included all the information requested in your manuscript?	Yes
**Resources**<br><br>A description of all resources used, including antibodies, cell lines, animals and software tools, with enough information to allow them to be uniquely identified, should be included in the Methods section. Authors are strongly encouraged to cite Research Resource Identifiers (RRIDs) for antibodies, model organisms and tools, where possible.	Yes

Have you included the information requested as detailed in our Minimum Standards Reporting Checklist?	
**Availability of data and materials**<br><br>All datasets and code on which the conclusions of the paper rely must be either included in your submission or deposited in publicly available repositories (where available and ethically appropriate), referencing such data using a unique identifier in the references and in the “Availability of Data and Materials” section of your manuscript.<br><br>Have you have met the above requirement as detailed in our Minimum Standards Reporting Checklist?	Yes

Manuscript Click here to download Manuscript Manuscript.docx

Chromosome-level reference genome of the jellyfish (*Rhopilema esculentum*)

Yunfeng Li[1,†], Lei Gao[1,†], Yongjia Pan[1,†], Meilin Tian[1], Yulong Li[1], Chongbo He[1], Ying Dong[1], Yamin Sun[2,*], Zunchun Zhou[1,*]

[1]Liaoning Ocean and Fisheries Science Research Institute, 50 Heishijiao St., Dalian, Liaoning 116023, China, [2]Tianjin Biochip Corporation, 23 Hongda St., Tianjin 300457, China

***Correspondence address.** Zunchun Zhou, Liaoning Ocean and Fisheries Science Research Institute, 50 Heishijiao St., Dalian, Liaoning 116023, China, E-mail: zunchunz@hotmail.com; Yamin Sun, Tianjin Biochip Corporation, 23 Hongda St., Tianjin 300457, China, E-mail: nksunyamin@aliyun.com.

[†]These authors contributed equally to this work.

Abstract

Background: Jellyfish belongs to Cnidaria and it occupies an important phylogenetic location together with Porifera as one of the earliest branching Metazoa lineages after their divergence with Ctenophora. The jellyfish *Rhopilema esculentum* is important fishery resource in China. However, genome resource of *R. esculentum* has not been reported to date. **Findings:** We constructed a chromosome-level genome assembly of *R. esculentum* using Pacific Biosciences, Illumina and Hi-C sequencing technologies. A total of 22.6 Gb and 39.76 Gb raw reads were generated using the Illumina HiSeq2500 platform and the PacBio Sequel platform, respectively. The final genome assembly was approximately 275.42 Mb, with a contig N50 length of 1.13 Mb. Using Hi-C technology to identify the contacts among contigs, 260.17 Mb (94.46%) of the assemblies were anchored onto 21 pseudochromosomes with a scaffold N50 of 12.97 Mb. We identified 17,219 protein-coding genes, with an average CDS length of 1,575 bp. The genome-wide phylogenetic analyses indicated that *R. esculentum* was less evolved than other species in Scyphozoa. In addition, 127 toxin-like genes were identified, and one toxin-related "hub" was found by genomic survey. **Conclusions:** We first finished chromosome-level genome assembly of *R. esculentum*, which will provide a valuable genomic background for the studies of the jellyfish biology, the evolutionary history of Cnidaria and the pharmacology of jellyfish.

Keywords: Jellyfish; *Rhopilema esculentum*; whole genome sequencing; chromosome-level assembly; toxin-like genes

Data Description

Background

Jellyfish belongs to Cnidaria and it occupies an important phylogenetic location together with Porifera as one of the earliest branching Metazoa lineages after their divergence with Ctenophora [1]. The jellyfish *Rhopilema esculentum* Kishinouye, an edible species in the Class Scyphozoa (also named true jellyfish), is widely distributed in the seas around China, Japan and Korea [2], and is one of the most abundant fishery animals in China. It has been exploited as food in China for thousands of years and is gaining more attention recently due to its pharmacological properties [3]. In contrast to many other jellyfish species, which have always raised concerns due to their harmfulness to industry and the community in blooms [4], the population of *R. esculentum* has declined in recent years for overfishing. The stock enhancement and aquaculture have been initiated to meet the expanding market demand of *R. esculentum*, accounting for approximately 82,280 tons/year of the production and 122,800,000 dollars/year of the profit in China, respectively [5]. The lack of genomic resource limits the study of jellyfish phylogeny and the investigation of many specific characteristics. Recently, several genome assemblies have been reported for the species in the class Scyphozoa, including the moon jellyfish (*Aurelia* and *Aurelia aurita*) [6, 7], the giant Nomura's jellyfish (*Nemopilema nomurai*) [8] and the upside-down jellyfish (*Cassiopea xamachana*) [9], mainly concerning the increasing jellyfish blooms throughout the world [10]. However, no chromosome-level reference genome has been reported in the class Scyphozoa, and there is very limited information on the genome

architecture of *R. esculentum*. In the present study, the chromosome-level genome of *R. esculentum* was sequenced, assembled and annotated, which will improve our understanding of evolutionary characteristics and pharmacology of jellyfish.

Sample and sequencing

R. esculentum samples were collected from Yingkou, Liaoning Province, China (Fig. 1). Genomic DNA was extracted from the ectoderm tissue using TIANamp Marine Animal DNA Kits (Tiangen, Beijing, China) and used for sequencing. Genomic DNA was sheared using a sonication device for short-insert paired-end (PE) library construction. The short-insert libraries with a size of 500 bp were constructed according to the instruction described in the Illumina library preparation kit. All libraries were sequenced on an Illumina HiSeq2500 platform (Illumina, San Diego, CA, USA) with paired-ends and 150 bp read length. In total, approximately 22.6 Gb (77×) of raw data were generated, and 20.03 Gb (68×) of clean data were filtered by FastQC (v0.11.2) (Supplementary Table S1). For PacBio library construction, genomic DNA of *R. esculentum* was sheared to ~20 kb, and the short fragments below the size of 7 kb were filtered using BluePipin (Sage Science, MA, USA). Filtered DNA was then converted into the proprietary SMRTbell library using the PacBio DNA Template Preparation Kit. In total, 39.76 Gb (135×) of quality-filtered data with a mean length of 7,196 bp were obtained from the PacBio Sequel platform (Supplementary Table S1).

Genome size and heterozygosity estimation

The distribution of *k*-mer frequency, also known as the *k*-mer spectrum, is widely used for the estimation of genome size. We adapted a method using Jellyfish software based on a *k*-mer distribution [11] to estimate the genome size with high quality reads above Q20 from short-insert size libraries (500 bp). We obtained a *k*-mer (K=17) depth distribution from the Jellyfish analysis and clearly observed the peak depth from the distribution data. To get the estimation of genome size, the following formula was applied: genome size = total_*k*-mer_num/*k*-mer_depth, where total_*k*-mer_num (1.92×10^{10}) is the total number of *k*-mers from all reads, and *k*-mer_depth (63) is the peak depth (Supplementary Fig. S1). Based on this method, the genome size and the heterozygosity rate were estimated to be 301 Mb and 1.42%, respectively. *K*-mer analysis also indicated that the repetitive sequences accounted for approximately 44.28% of the genome.

Genome assembly and annotation

In the present study, the long reads of PacBio sequencing data helped to solve the high level of heterozygosity, which is one of the main challenges in the genome assembly of marine invertebrates [12, 13]. The genome assembly was performed using software wtdbg2 with default parameters (https://github.com/ruanjue/wtdbg2). The assembly sequence was then polished using Quiver (SMRT Analysis v2.3.0) with default parameters. To achieve higher continuity and accuracy of the assembly genome, 5 rounds of iterative error correction were performed using the Illumina clean genome data by in-house script. Finally, a genome of 275.42 Mb was assembled, with 760

contigs and a contig N50 size of 1.13 Mb (Table 1 and Supplementary Fig. S2).

Both RepeatModeler and RepeatMasker (http://www.repeatmasker.org) were used to perform *de novo* identification and masking of repeat sequences. To ensure the integrity of genes in the subsequent analysis, the low complexity or simple repeats were not masked in this analysis because some of these repeats could be found in genes. Finally, 29.23% of assembled bases (80,495,815 bp) were masked (Supplementary Table S2). Of these, 9.93% could be annotated with known repeat families, and 19.30% were unclassified repeats.

Protein-coding region identification and gene prediction were performed through a combination of *ab initio* prediction, homology-based prediction and transcriptome-based prediction methods. The *ab initio* gene prediction was conducted with Augustus (version 2.5.5) [14], GlimmerHMM (version 3.0.1) [15] and SNAP15 [16] to predict coding genes. For the homology-based prediction, homologous proteins of several Cnidaria species (myxosporean (*Thelohanellus kitauei*), coral (*Stylophora pistillata* and *Orbicella faveolata*), hydrozoan (*Hydra vulgaris*), sea anemone (*Exaiptasia pallida*) and the Cnidaria EST database) were downloaded from NCBI and aligned to the assembled genome. Then, Genewise (version 2.2.0) [17, 18] was used to generate gene structures based on the homology alignments. For transcriptome-based prediction, transcriptome data were generated from scyphistoma, strobili, ephyra and juvenile medusa on the Iillumina HiSeq 3500 platform (154.6 Gb clean reads) (Supplementary Table S3) and then mapped to the genome assembly using TopHhat (version 2.0.8) [19]. Cufflinks (version 2.1.1) [20] (http://cufflinks.cbcb.umd.edu/) was then used to identify

spliced transcripts in the gene models. All the gene evidence predicted from the above three approaches were integrated by EvidenceModeler (EVM) [21] into a weighted and non-redundant consensus of the gene structures. A total of 17,219 genes, with an average CDS length of 1,575 bp, were finally predicted to be present in the genome of *R. esculentum* (Table 1). All the gene sequences were mapped to several public databases, including NR [22], GO (Supplementary Fig. S3) [23], Swiss [24], KOG (Supplementary Fig. S4) [25] and KEGG [26], to obtain the functional annotation. A total of 16,713 genes (97.1%) could be mapped to at least one database, and 8,880 genes were annotated in all four databases (Supplementary Fig. S5).

Quality assessment

We first aligned all the Illumina genome reads with the *R. esculentum* assembly genome using the Burrows-Wheeler Aligner (BWA, version 0.7.17) to evaluate the coverage of the genome. The percentage of aligned reads was estimated to be 99.81%. BUSCO (version: 3.0.2) [27] was then used to evaluate the integrity of the genome (Supplementary Table S4). The values of core gene estimation were calculated as follows: C: 97.0% [S: 92.1%, D: 5.0%], F: 1.7%, M: 1.3%, n: 303, wherein C, S, D, F, M and n indicated complete BUSCOs, complete and single-copy BUSCOs, complete and duplicated BUSCOs, fragmented BUSCOs, missing BUSCOs and total BUSCO groups searched, respectively. The results indicated that the assembly covered most genetic regions, further confirming the assembly quality of the *R. esculentum* genome.

Pseudochromosome construction

Hi-C experiments were used for the chromosome assembly of jellyfish. The whole-body homogenate of jellyfish was fixed in 1% (vol/vol) formaldehyde and was then used for the preparation of in situ Hi-C libraries. Nuclei extraction and permeabilization, chromatin digestion and proximity-ligation treatments were performed essentially as previously described [28]. MboI was used as the restriction enzyme. Libraries were sequenced on the Illumina X-TEN platform (San Diego, CA, USA) with 2×150 bp reads. They were independently analyzed in the HiC-Pro pipeline (default parameters and LIGATION_SITE = GATC) [29]. A total of 23.96 Gb of trimmed reads were obtained, accounting for around 82-fold coverage of the jellyfish genome. The 3D-DNA was used to assign the order and orientation of each group [30]. The contact maps were plotted using HiCPlotter software [31]. Finally, 260.17 Mb (94.46%) of the assemblies were anchored onto 21 pseudochromosomes with a scaffold N50 of 12.97 Mb (Fig. 2, Supplementary Fig. S6 and Supplementary Table S5), which was in agreement with the Karyotype (2n=42) of jellyfish [32].

Phylogenetic analysis

To examine the evolutionary relationships of jellyfish and other species, the whole protein sequences of 13 species, including species from Ctenophora (ctenophore (*Mnemiopsis leidyi*)), Porifera (demosponge (*Amphimedon queenslandica*)), Placozoa (*Trichoplax adhaerens*), Cnidaria (jellyfish (*R. esculentum* and *Aurelia*), Hydrozoa (*H. vulgaris*), coral (*S. pistillata*), sea anemone (*Nematostella vectensis*)), Protostomia

(Lophotrochozoa (pacific oyster (*Crassostrea gigas*)), Ecdysozoa (cladoceran (*Daphnia pulex*))), and Deuterostomia (Echinodermata (sea urchin (*Strongylocentrotus purpuratus*)), Hemichordata (acorn worm (*Saccoglossus kowalevskii*)), Chordata (zebrafish (*Danio rerio*))) were analyzed. All data of the other 12 species were obtained from Ensembl or NCBI. Gene family analysis was performed using OrthoMCL [33]. In detail, the protein-coding genes from the above sequenced genomes were aligned to each other using the BLASTP program [34]. Similarity information from the pair-wise sequence alignments was used as distance parameters for the gene family clustering by MCL with an inflation value of 1.5.

A set of 32,138 gene families were eventually identified among the 13 species, of which 2,092 families were present in all 13 species (Fig. 3 and Supplementary Table S6). A total of 335 single-copy orthologous genes were selected for further alignment using MUSCLE (v3.6) [35] and then concatenated into a single multiple sequence alignment by an in-house Perl script. A maximum likelihood phylogeny was reconstructed using RAxML [36] (Fig. 4). The results supported the view that *R. esculentum* and *H. vulgaris* are sister groups. Based on the phylogeny and fossil records, we dated the divergence time of *R. esculentum* and *H. vulgaris* to approximately 501.71 million years ago (mya), which was consistent with previous studies [37]. To compare genomic traits with other species, we performed a comparative genomic analysis among the above 13 species using CAFE software (Supplementary Table S7) [38]. It was found that 27 and 27 gene families were significantly expanded or contracted in *R. esculentum*, respectively ($P < 0.05$) (Supplementary Table S8 and Supplementary Table S9).

Interestingly, the gene families enriched in the GO category of transmembrane transport were significantly expanded, and the relative GO sub-categories included drug transmembrane transport, drug transmembrane transporter activity, ion transmembrane transporter activity and amino acid transmembrane transporter activity. The venom action, which is always characterized in jellyfish species, may contribute to the gene expansion in transmembrane transport [39, 40].

A comparative genomic analysis was performed among the four jellyfish species in the Class Scyphozoa (including *R. esculentum, Aurelia, N. nomurai* and *C. xamachana*) and *H. vulgaris* (used as outgroup, and also used to calculate the divergence time). A total of 244 unique gene families that could be annotated in NR database were identified in *R. esculentum*. It was surprising that more than half of those (136 unique gene families) were annotated with the proteins of the species in Anthozoa. This was also supported by the results of phylogenetic analysis among the 13 species (Fig. 4). Compared to *H. vulgaris* and *Aurelia*, *R. esculentum* had fewer gene gains (331) and fewer gene losses (294). The above results implied that some gene families those were possessed by the last common ancestor of Anthozoa and Scyphozoa were kept by Anthozoa species and *R. esculentum*, but were lost in other Scyphozoa species.

To further explore this hypothesis, the level of positive selection was analyzed between Scyphozoa species and *H. vulgaris* (*R. esculentum vs. H. vulgaris* (Re-Hv), *A. aurita vs. H. vulgaris* (Aa-Hv), *N. nomurai vs. H. vulgaris* (Nn-Hv), *C. xamachana vs. H. vulgaris* (Cx-Hv)), using ParaAT and KaKs_Calculator [41, 42]. There were 7542, 7864, 7611 and 6141 orthogroups found in Re-Hv, Aa-Hv, Nn-Hv and Cx-Hv,

respectively. The number of positive selective orthogroups with Ka/Ks>0.5 in Re-Hv was 62 [43], which was fewer than those of Aa-Hv (83), Nn-Hv (99) and Cx-Hv (71). Therefore, it indicated that *R. esculentum* kept more genes from the ancestor and was less evolved compared to other Scyphozoa species.

Analysis of toxin-like genes in jellyfish

Jellyfish, one of the main subgroups of Cnidaria, is one of the oldest extant lineages of venomous animals [44, 45]. The venom, stored in nematocysts, is injected into the victim or prey when triggered to discharge. Jellyfish stings are dangerous to swimmers and fishermen and can cause local oedema, vesicular eruption, shock, and even death [10, 46]. The jellyfish venom consists of polypeptides, enzymes and some non-protein bioactive components [45] with various bioactivities, such as neurotoxins, myotoxins, hemolytic toxins and cardiotoxins [47]. The venom constituents of jellyfish are already used in pharmacological studies in the recent years. Omics analyses, especially transcriptomic and proteomic analyses, have been used to conduct large-scale identification of toxins and related genes from jellyfish, and many putative toxins have been identified [44, 46-49]. However, due to the absence of genome information and the limitation of sampling [46], the overall understanding of toxin-like genes is limited, which may be responsible for the poor consistency of the results among previous studies [49]. Here, we conducted a genomic survey of toxin-like genes in the assembled jellyfish genome.

First, the genes were identified using BLASTP with a cutoff *E-value* of $1e^{-10}$

against the database of animal toxin annotation project (Tox-Prot) in UniProt. Second, according to the gene annotations of NR, Uniprot and Tox-Prot, the genes annotated consistently were chosen as the toxin-like genes. Third, to make the pool of venom-related genes more complete, we screened all the genes predicted in the genome manually by their annotation.

There were 127 toxin-like genes identified, including 60 metalloproteinases, 18 phospholipases, 13 nucleases and nucleotidases, 13 peptidases and inhibitors, 12 genes with toxin activity and 11 other venom-related genes (Table 2). It is not surprising that metalloprotease was the most abundant group of toxins because they are widely considered to be a key toxic component in various venomous animals, such as spiders [50], snakes [51], scorpions [52] and jellyfish [46, 53]. Metalloprotease can interfere with blood coagulation and induce necrosis and can be related to the symptoms of stings, such as swelling, myonecrosis, inflammation and blister formation [45, 49].

Phospholipases, the second most abundant toxins, were identified with various isoforms, such as phospholipase A2, acidic phospholipase A2 PA4, phospholipase A2 isozymes PA3A/PA3B/PA5 and putative phospholipase B-like 2. Phospholipases are ubiquitous in the venom of many toxic animals and exhibit various toxicities, among which hemolytic activity is the most striking [47]. High levels of phospholipase A2 activity were observed in the tentacles of scyphozoan and cubozoan species [47, 54] and were presumably involved in the defence against and digestion of prey [45]. In the present study, it was also found that nine copies of phospholipase were duplicated in tandem fashion located on three loci of the genome, suggesting their important roles

during evolution.

Two copies of jellyfish toxin were also found. The jellyfish toxins, also called cubozoan-related porins, were highly abundant in cubozoan venoms [48] and were also reported in other medusozoans, such as Scyphozoa [47], Hydrozoa [55] and Anthozoa [56]. They are potent and rapid-acting toxins with hemolytic and pore-forming activity [45, 47]. Compared to the high abundance in cubozoans, such as 15 isoforms found in *Chironex fleckeri*, the relatively fewer copies in scyphozoan species may lead to less severe stings, which is consistent with the report of DL Brinkman et al. [48].

Three new toxin-like genes that have not been previously reported in jellyfish were identified in the present study. Prothrombin, with the activity of snake prothrombin activator annotated by Tox-Prot, was found to have two copies in the genome of the *R. esculentum*. Prothrombin can attack the hemostatic system of prey by converted into thrombin with serine-type endopeptidase activity [57, 58]. Reticulocalbin is known to have calcium ion-binding activity. Its role in venom is still unclear, though it was speculated to play a potential unknown role in prey incapacitation by binding phospholipase A2 [59, 60]. Lysosomal acid phosphatase is an ortholog of venom acid phosphatase, which is an acidic heat-labile protein with carbohydrate IgE binding epitopes [61]. It was mainly identified in the honeybee and has been indicated to cause an allergic reaction [61-63]. The new toxin-like genes identified in this study will provide insight into the complex composition of jellyfish venom. When compared to the venom composition of the jellyfish *Stomolophus meleagris*, a closely related species of *R. esculentum*, it was noted that two types of main toxins were lost in *R. esculentum*,

including serine protease inhibitor (only one copy found) and potassium channel inhibitor ShK [46]. They are known to block the activity of trypsin and plasmin and function as neurotoxins. The different composition of venom may account for the different symptoms after sting.

Interestingly, some toxin-like genes were located closely on contig 521 as a “hub”, including four PLA2s, two ENPP5s, one TRPA1 and one SLC47A1 (Table 3). These toxins were associated with phospholipase A2 activity, nuclease activity, toxin activity and toxin extrusion. In addition, according to the chromosome-level analysis, contig 747 and contig 751 were found to be located on the two sides of contig 521 and had five and three toxin-like genes respectively. These three contigs were arranged in chromosome 7 (3691690~13486489 bp) as a head-to-tail tandem to be a bigger “hub”. The genes located closely in genome are always involved in related functions and are expressed in similar patterns [64-66]. It was also reported that neighboring genes tend to be co-expressed rather than expressed by chance [67]. Thus, it was suggested that contig747-contig521-contig751 tandem on chromosome 7 may play important roles in the venom formation and function of jellyfish. Further studies are needed to clarify its specific functions.

Availability of supporting data

The raw sequencing data of genome obtained by Illumina and PacBio platform are available via NCBI with accession numbers SRR8617500 and SRR8617499 respectively (BioProject accession number PRJNA523480). The raw sequencing data

of transcriptome are available via NCBI with accession numbers SRR8401786-SRR8401797 (BioProject accession number PRJNA512552).

Competing interests

The authors declare that they have no competing interests.

Abbreviations

CDS, Coding Domain Sequence; NCBI, National Center for Biotechnology Information; BLAST, Basic Local Alignment Search Tool; BUSCO, Benchmarking Universal Single-Copy Orthologs; GO, Gene Ontology; NR, Non Redundant database; KOG, Eukaryotic Orthologous Groups; KEGG, Kyoto Encyclopedia of Genes and Genomes; CAFE, computational analysis of gene family evolution.

Ethics statement

This study was approved by the Animal Care and Use Committee of Liaoning Ocean and Fisheries Science Research Institute. This study did not involve endangered or protected species.

Author contributions

Z.Z. and Y.L. designed the project. M.T. and Y.L. collected the samples. Y.P., C.H. and Y.D. extracted the genomic DNA. L.G., Y.S. and Y.P. were involved in the data analyses. L.G. and Y.S. wrote the manuscript. All authors read and approved the final manuscript.

Funding

This work was supported by the National Natural Science Foundation of China (31302173; 31602156; 31602155); the Science and Technology Program of Liaoning Province, China (2013203001); the Natural Science Foundation of Liaoning Province, China (20180551158); the Scientific Research Program of Ocean and Fisheries Administration of Liaoning Province, China (201827); Liaoning Science Public Welfare Research Fund Project (20180015).

References

1. Dunn CW, Hejnol A, Matus DQ et al. Broad phylogenomic sampling improves resolution of the animal tree of life. Nature 2008;**452**(7188):745.
2. Dong Z, Liu D, Keesing JK. Contrasting trends in populations of *Rhopilema esculentum* and *Aurelia aurita* in Chinese waters. Jellyfish blooms. Springer; 2014. p. 207-18.
3. Zhuang Y, Hou H, Zhao X et al. Effects of collagen and collagen hydrolysate from jellyfish (*Rhopilema esculentum*) on mice skin photoaging induced by UV irradiation. J Food Sci 2009;**74**(6):H183-H8.
4. Dong Z, Liu D, Keesing JK. Jellyfish blooms in China: dominant species, causes and consequences. Mar Pollut Bull 2010;**60**(7):954-63.
5. Ministry of Agriculture Bureau of Fisheries. 2018 China Fisheries Statistical Yearbook. Beijing: China Agriculture Publishing Company; 2018.
6. Gold DA, Katsuki T, Li Y et al. The genome of the jellyfish *Aurelia* and the evolution of animal complexity. Nat Ecol Evol 2019;**3**(1):96.
7. Khalturin K, Shinzato C, Khalturina M et al. Medusozoan genomes inform the evolution of the jellyfish body plan. Nat Ecol Evol 2019;**3**(5):811.
8. Kim H-M, Weber JA, Lee N et al. The genome of the giant Nomura's jellyfish sheds light on the early evolution of active predation. BMC Biology 2019;**17**(1):28.
9. Ohdera A, Ames CL, Dikow RB et al. Box, stalked, and upside-down? Draft genomes from diverse jellyfish (Cnidaria, Acraspeda) lineages: *Alatina alata* (Cubozoa), *Calvadosia cruxmelitensis* (Staurozoa), and *Cassiopea xamachana* (Scyphozoa). GigaScience 2019;**8**(7):giz069.
10. Lee H, Jung E, Kang C et al. Scyphozoan jellyfish venom metalloproteinases and their role in the cytotoxicity. Toxicon 2011;**58**(3):277-84.
11. Marçais G, Kingsford C. A fast, lock-free approach for efficient parallel counting of occurrences of *k*-mers. Bioinformatics 2011;**27**(6):764-70.
12. Zhang X, Sun L, Yuan J et al. The sea cucumber genome provides insights into morphological

evolution and visceral regeneration. PLoS Biol 2017;**15**(10):e2003790.

13. Zhang G, Fang X, Guo X et al. The oyster genome reveals stress adaptation and complexity of shell formation. Nature 2012;**490**(7418):49-54.
14. Stanke M, Diekhans M, Baertsch R et al. Using native and syntenically mapped cDNA alignments to improve *de novo* gene finding. Bioinformatics 2008;**24**(5):637-44.
15. Majoros WH, Pertea M, Salzberg SL. TigrScan and GlimmerHMM: two open source *ab initio* eukaryotic gene-finders. Bioinformatics 2004;**20**(16):2878-9.
16. Korf I. Gene finding in novel genomes. BMC Bioinformatics 2004;**5**(1):59.
17. Birney E, Durbin R. Using GeneWise in the *Drosophila* annotation experiment. Genome Res 2000;**10**(4):547-8.
18. Birney E, Clamp M, Durbin R. GeneWise and genomewise. Genome Res 2004;**14**(5):988-95.
19. Trapnell C, Pachter L, Salzberg SL. TopHat: discovering splice junctions with RNA-Seq. Bioinformatics 2009;**25**(9):1105-11.
20. Trapnell C, Roberts A, Goff L et al. Differential gene and transcript expression analysis of RNA-seq experiments with TopHat and Cufflinks. Nature protocols 2012;**7**(3):562.
21. Haas BJ, Salzberg SL, Zhu W et al. Automated eukaryotic gene structure annotation using EVidenceModeler and the Program to Assemble Spliced Alignments. Genome Biol 2008;**9**(1):1.
22. Benson DA, Karsch-Mizrachi I, Lipman DJ et al. GenBank. Nucleic Acids Res 2005;**33**(suppl_1):D34-D8.
23. Consortium GO. Gene Ontology annotations and resources. Nucleic Acids Res 2012;**41**(D1):D530-D5.
24. Bairoch A, Apweiler R. The SWISS-PROT protein sequence database and its supplement TrEMBL in 2000. Nucleic Acids Res 2000;**28**(1):45-8.
25. Tatusov RL, Fedorova ND, Jackson JD et al. The COG database: an updated version includes eukaryotes. BMC Bioinformatics 2003;**4**(1):41.
26. Kanehisa M, Goto S. KEGG: kyoto encyclopedia of genes and genomes. Nucleic Acids Res 2000;**28**(1):27-30.
27. Waterhouse RM, Seppey M, Simão FA et al. BUSCO applications from quality assessments to gene prediction and phylogenomics. Mol Biol Evol 2017;**35**(3):543-8.
28. Zhu W, Hu B, Becker C et al. Altered chromatin compaction and histone methylation drive non-additive gene expression in an interspecific *Arabidopsis* hybrid. Genome Biol 2017;**18**(1):157.
29. Servant N, Varoquaux N, Lajoie BR et al. HiC-Pro: an optimized and flexible pipeline for Hi-C data processing. Genome Biol 2015;**16**:259.
30. Dudchenko O, Batra SS, Omer AD et al. De novo assembly of the *Aedes aegypti* genome using Hi-C yields chromosome-length scaffolds. Science 2017;**356**(6333):92-5.
31. Akdemir KC, Chin L. HiCPlotter integrates genomic data with interaction matrices. Genome Biol 2015;**16**(1):198.
32. Guo P. The karyotype of *Rhopilcma esculenta*. Journal of Fisheries of China 1994;**18**(3):253-5.
33. Li L, Stoeckert CJ, Roos DS. OrthoMCL: identification of ortholog groups for eukaryotic genomes. Genome Res 2003;**13**(9):2178-89.
34. Altschul SF, Madden TL, Schäffer AA et al. Gapped BLAST and PSI-BLAST: a new generation of protein database search programs. Nucleic Acids Res 1997;**25**(17):3389-402.
35. Edgar RC. MUSCLE: multiple sequence alignment with high accuracy and high throughput. Nucleic Acids Res 2004;**32**(5):1792-7.

36. Stamatakis A. RAxML version 8: a tool for phylogenetic analysis and post-analysis of large phylogenies. Bioinformatics 2014;**30**(9):1312-3.
37. Park E, Hwang D-S, Lee J-S et al. Estimation of divergence times in cnidarian evolution based on mitochondrial protein-coding genes and the fossil record. Mol Phylogenet Evol 2012;**62**(1):329-45.
38. De Bie T, Cristianini N, Demuth JP et al. CAFE: a computational tool for the study of gene family evolution. Bioinformatics 2006;**22**(10):1269-71.
39. Grishin EV. Neurotoxin from black widow spider venom structure and function. Natural Toxins 2. Springer; 1996. p. 231-6.
40. Meldolesi J, Scheer H, Madeddu L et al. Mechanism of action of α-latrotoxin: the presynaptic stimulatory toxin of the black widow spider venom. Trends Pharmacol Sci 1986;**7**:151-5.
41. Zhang Z, Xiao J, Wu J et al. ParaAT: a parallel tool for constructing multiple protein-coding DNA alignments. Biochem Bioph Res Co 2012;**419**(4):779-81.
42. Zhang Z, Li J, Zhao X-Q et al. KaKs_Calculator: calculating Ka and Ks through model selection and model averaging. Genom Proteom Bioinf 2006;**4**(4):259-63.
43. Swanson WJ, Wong A, Wolfner MF et al. Evolutionary expressed sequence tag analysis of *Drosophila* female reproductive tracts identifies genes subjected to positive selection. Genetics 2004;**168**(3):1457-65.
44. Jaimes-Becerra A, Chung R, Morandini AC et al. Comparative proteomics reveals recruitment patterns of some protein families in the venoms of Cnidaria. Toxicon 2017;**137**:19-26.
45. Jouiaei M, Yanagihara A, Madio B et al. Ancient venom systems: a review on cnidaria toxins. Toxins 2015;**7**(6):2251-71.
46. Li R, Yu H, Xue W et al. Jellyfish venomics and venom gland transcriptomics analysis of *Stomolophus meleagris* to reveal the toxins associated with sting. J Proteomics 2014;**106**:17-29.
47. Liu G, Zhou Y, Liu D et al. Global transcriptome analysis of the tentacle of the jellyfish *Cyanea capillata* using deep sequencing and expressed sequence tags: Insight into the toxin-and degenerative disease-related transcripts. PloS one 2015;**10**(11):e0142680.
48. Brinkman DL, Jia X, Potriquet J et al. Transcriptome and venom proteome of the box jellyfish *Chironex fleckeri*. BMC Genomics 2015;**16**(1):407.
49. Li R, Yu H, Yue Y et al. Combined proteomics and transcriptomics identifies sting-related toxins of jellyfish *Cyanea nozakii*. J Proteomics 2016;**148**:57-64.
50. Trevisan-Silva D, Gremski LH, Chaim OM et al. Astacin-like metalloproteases are a gene family of toxins present in the venom of different species of the brown spider (genus *Loxosceles*). Biochimie 2010;**92**(1):21-32.
51. Markland Jr FS, Swenson S. Snake venom metalloproteinases. Toxicon 2013;**62**:3-18.
52. Brazón J, Guerrero B, D'Suze G et al. Fibrin(ogen)olytic enzymes in scorpion (*Tityus discrepans*) venom. Comp Biochem Physiol B, Biochem Mol Biol 2014;**168**:62-9.
53. Jouiaei M, Casewell NR, Yanagihara AA et al. Firing the sting: chemically induced discharge of cnidae reveals novel proteins and peptides from box jellyfish (*Chironex fleckeri*) venom. Toxins 2015;**7**(3):936-50.
54. Nevalainen TJ, Peuravuori HJ, Quinn RJ et al. Phospholipase A2 in cnidaria. Comp Biochem Physiol B, Biochem Mol Biol 2004;**139**(4):731-5.
55. Brinkman DL, Konstantakopoulos N, McInerney BV et al. *Chironex fleckeri* (box jellyfish) venom proteins: expansion of a cnidarian toxin family that elicits variable cytolytic and cardiovascular effects. J Biol Chem 2014:jbc. M113. 534149.

56. Jouiaei M, Sunagar K, Federman Gross A et al. Evolution of an ancient venom: recognition of a novel family of cnidarian toxins and the common evolutionary origin of sodium and potassium neurotoxins in sea anemone. Mol Biol Evol 2015;**32**(6):1598-610.
57. Rosing J, Tans G. Structural and functional properties of snake venom prothrombin activators. Toxicon 1992;**30**(12):1515-27.
58. Kini RM, Morita T, Rosing J. Classification and nomenclature of prothrombin activators isolated from snake venoms. Thromb Haemost 2001;**86**:710-1.
59. Dodds DN, Schlimgen AK, Lu SY et al. Novel reticular calcium binding protein is purified on taipoxin columns. J Neurochem 1995;**64**(5):2339-44.
60. Margres MJ, McGivern JJ, Wray KP et al. Linking the transcriptome and proteome to characterize the venom of the eastern diamondback rattlesnake (*Crotalus adamanteus*). J Proteomics 2014;**96**:145-58.
61. Hoffman D, Weimer E, Sakell R et al. Sequence and characterization of honeybee venom acid phosphatase. J Allergy Clin Immun 2005;**115**(2):S107.
62. Grunwald T, Bockisch B, Spillner E et al. Molecular cloning and expression in insect cells of honeybee venom allergen acid phosphatase (Api m 3). J Allergy Clin Immun 2006;**117**(4):848-54.
63. Kim BY, Jin BR. Molecular characterization of a venom acid phosphatase Acph-1-like protein from the Asiatic honeybee *Apis cerana*. J Asia-Pac Entomol 2014;**17**(4):695-700.
64. Williams EJ, Bowles DJ. Coexpression of neighboring genes in the genome of *Arabidopsis thaliana*. Genome Res 2004;**14**(6):1060-7.
65. Michalak P. Coexpression, coregulation, and cofunctionality of neighboring genes in eukaryotic genomes. Genomics 2008;**91**(3):243-8.
66. Sémon M, Duret L. Evolutionary origin and maintenance of coexpressed gene clusters in mammals. Mol Biol Evol 2006;**23**(9):1715-23.
67. Schmid M, Davison TS, Henz SR et al. A gene expression map of *Arabidopsis thaliana* development. Nat Genet 2005;**37**(5):501-6.

 Click here to download Table Tables.docx

Table 1: Statistics of the assembly and annotation of jellyfish genome.

Genome feature	Parameter
**Genome assembly**	
Total length (Mb)	275.42
Contig N50 (Mb)	1.13
Longest contig (Mb)	6.59
Contig number	760
GC content (%)	36.25
Pseudochromosome number	21
Scaffold N50 (Mb)	12.97
**Genome annotation**	
Gene number	17,219
Gene density (per 100kb)	62.52
Average CDS length (bp)	1,575
Average exon length (bp)	198.8
Average intron length (bp)	987.2
Exon number per Gene	7.92
Exon GC content (%)	42.29

Table 2: Summary of all identified toxin-like genes from the genome of jellyfish *R. esculentum*. Full gene names are provided in Appendix: Supplementary Table S10.

Gene	Copy number	Description	Family	Reported in jellyfish
phospholipase A2	9	phospholipase A2 activity	phospholipase A2 family	YES
Acidic phospholipase A2 PA4	4	phospholipase A2 activity	phospholipase A2 family	YES
Phospholipase A2 isozymes PA3A/PA3B/PA5	4	phospholipase A2 activity	phospholipase A2 family	YES
Putative phospholipase B-like 2	1	hydrolase activity	phospholipase B-like family	YES
Zinc metalloproteinase nas	39	metalloendopeptidase activity		YES
Disintegrin and metalloproteinase	21	metalloendopeptidase activity		YES
Ectonucleotide pyrophosphatase/phosphodiesterase	8	nuclease activity	nucleotide pyrophosphatase/ phosphodiesterase family	YES
5'-nucleotidase	5	5'-nucleotidase activity	5'-nucleotidase family	YES
serine carboxypeptidase	1	serine-type carboxypeptidase activity	peptidase S10 family	YES
serine protease	7	serine-type endopeptidase activity	peptidase S1 family	YES
Prothrombin	2	serine-type endopeptidase activity	peptidase S1 family	NO
Dipeptidyl peptidase 9	1	serine-type peptidase activity	peptidase S9B family	YES
Kunitz-type_serine_protease_inhibitor	1	serine-type endopeptidase inhibitor activity	venom Kunitz-type family	YES
Cystatin	1	cysteine-type endopeptidase inhibitor activity	cystatin family	YES
Plancitoxin-1	3	toxin activity	DNase II family	YES
Ryncolin	6	toxin activity	ficolin lectin family	YES
Toxin TX	2	toxin activity	jellyfish toxin family	YES
Trpa1	1	toxin activity	(high similarity with Alpha-latrotoxin-Lt1a)	YES
Peroxiredoxin-4	2	protein homodimerization activity	peroxiredoxin family	YES
Glutaminyl-peptide cyclotransferase-like protein	1	glutaminyl-peptide cyclotransferase activity	glutaminyl-peptide cyclotransferase family	YES
Lysosomal acid lipase/cholesteryl ester hydrolase	1	lipase activity	Lipase family	YES
Trehalase	1	alpha-trehalase activity	glycosyl hydrolase 37 family	YES
Acetylcholinesterase	1	acetylcholinesterase activity	type-B carboxylesterase/lipase family	YES
Lysosomal acid phosphatase	1	acid phosphatase activity	histidine acid phosphatase family	NO
Reticulocalbin	1	calcium ion binding	CREC family	NO
Translationally-controlled tumor protein homolog	1	calcium ion binding	TCTP family	YES
Hyaluronidase-1	2	hyaluronan synthase activity	glycosyl hydrolase 56 family	YES

Table 3: The structure of toxin-related hub on contig 521. The arrows indicate the transcript direction. The green and yellow boxes indicate the identified and unidentified toxin-like genes respectively. Full gene names are provided in Appendix: Supplementary Table S10.

	Gene	ID	Description
↑	ENPP5	RE08134	nuclease activity
↑	ENPP5	RE08135	nuclease activity
↓	SLC35C2	RE08136	negative regulation of gene expression
↑	CNTNAP5	RE08137	cell adhesion
↑	TRPA1	RE08138	toxin activity
↓	ADAT1	RE08139	adenosine deaminase activity
↑	GABARAPL2	RE08140	autophagy
↑	OSP	RE08141	zinc ion binding
↓	EFCBP1	RE08142	calcium ion binding
↓	DIO1	RE08143	thyroxine 5'-deiodinase activity
↑	PLA2	RE08144	phospholipase A2 activity
↓	KIAA1468	RE08145	---
↓	YPT1	RE08146	GTPase activity
↓	GCSH	RE08147	shuttling the methylamine group of glycine
↑	SOXB2	RE08148	DNA binding
↑	C18ORF63	RE08149	---
↑	TBC1D20	RE08150	GTPase activator activity
↑	AARS	RE08151	alanine-tRNA ligase activity
↓	SAS10	RE08152	identical protein binding
↑	PLA2	RE08153	phospholipase A2 activity
↑	PLA2	RE08154	phospholipase A2 activity
↓	PLA2	RE08155	phospholipase A2 activity
↓	PDPR	RE08156	oxidoreductase activity
↓	PDPR	RE08157	oxidoreductase activity
↓	DHOD	RE08158	dihydroorotate dehydrogenase activity
↑	SLC47A1	RE08159	toxin extrusion

Figure Click here to download Figure Figures.docx

Figure 1: Picture of a jellyfish *R. esculentum* captured from Yingkou, Liaoning Province, China.

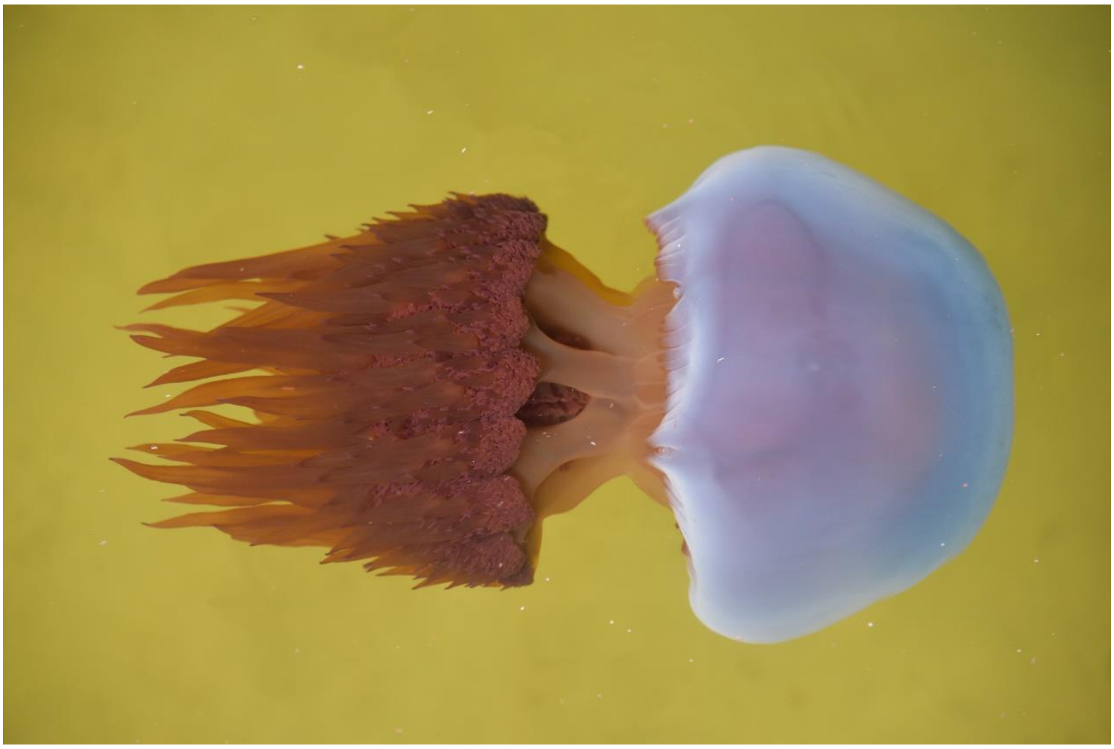

Figure 2: A schematic representation of the genomic characteristics of *R. esculentum*. Track A: 21 pseudochromosomes of the jellyfish genome. Track B: Protein-coding genes present in the scaffolds. Red represents genes on forward strand and green for genes on reverse strand. Track C: Distribution of gene density with sliding windows of 1 Mb. Higher density is shown in darker red color. Track D: Distribution of GC content in the genome. Track E: Distribution of repeat in the genome. Track F: Schematic presentation of major interchromosomal relationships.

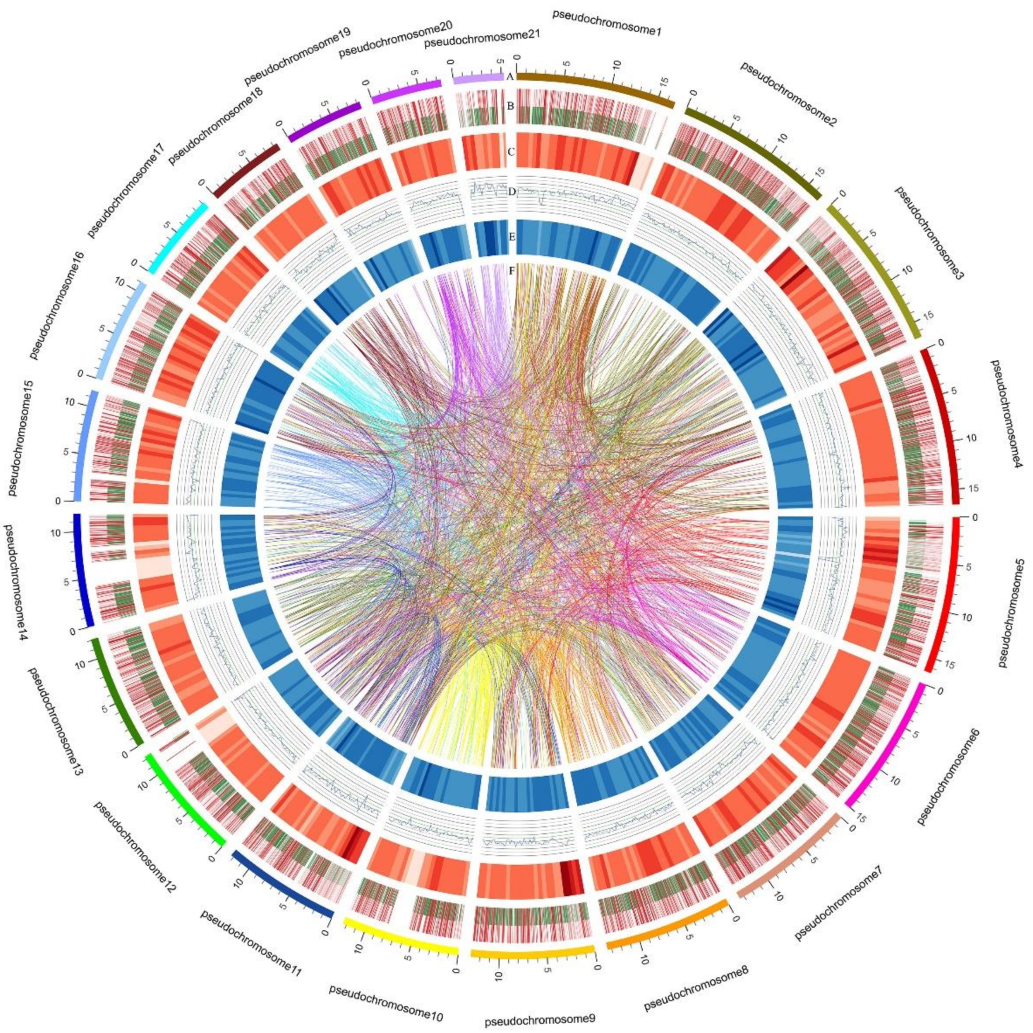

Figure 3: A Venn diagram of the orthologues shared among *R. esculentum*, *H. vulgaris*, *N. vectensis* and *A. aurita*.

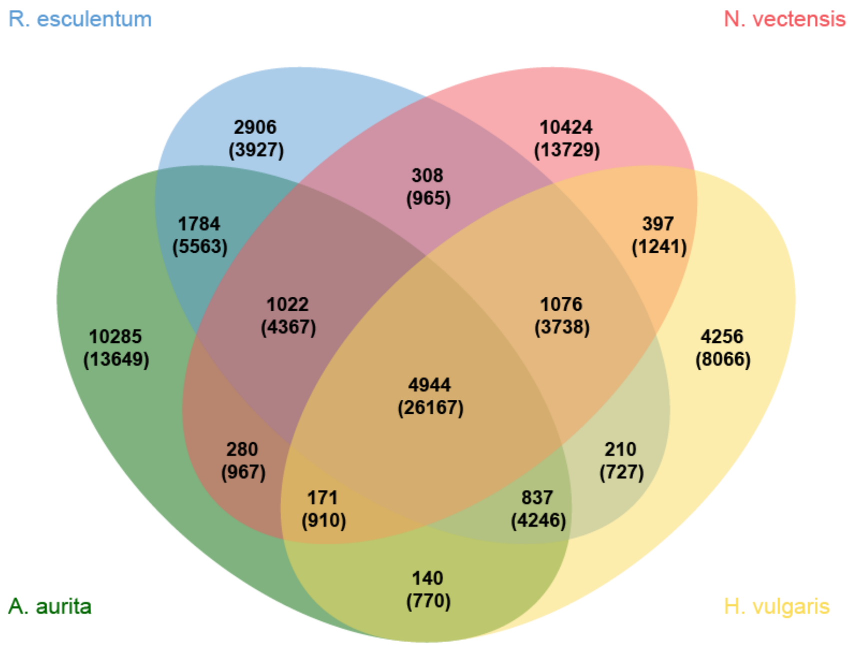

Figure 4: Phylogenetic analysis of *R. esculentum* with other metazoan species. The numbers of gene gains (+) and gene losses (−) are showed on the branches, which are also displayed as pie plots: the green part for gene gaining, the red part for gene losing and the blue part for gene remaining. The divergence times were dated and displayed below the phylogenetic tree.

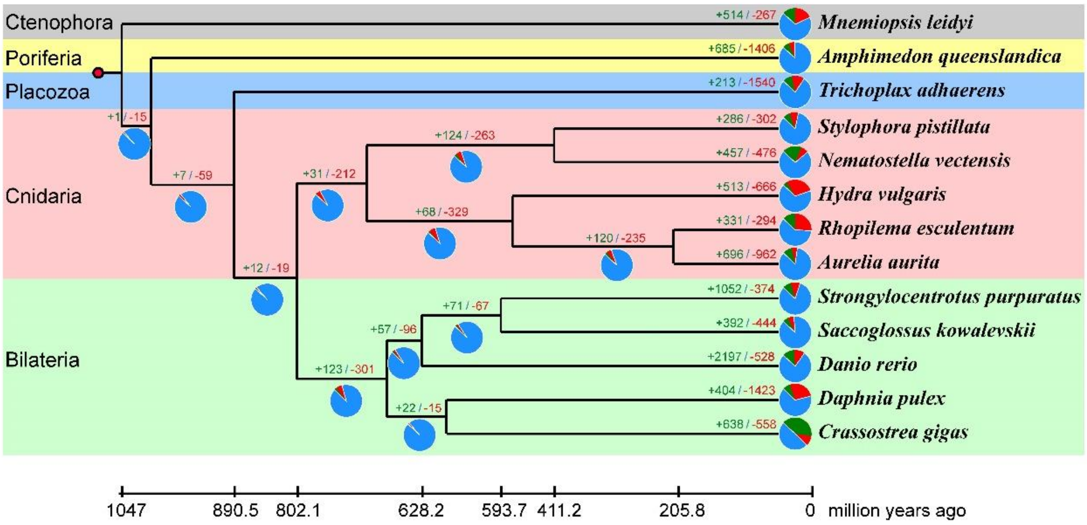

Click here to access/download
Supplementary Material
Supplemental tables and figures.docx
