# Supplementary material for: Chromosome-level reference genome of the jellyfish Rhopilema esculentum
Source: Gigascience. 2020 Apr 21;9(4):giaa036. doi: 10.1093/gigascience/giaa036 (PMC7172023; doi:10.1093/gigascience/giaa036)
Supplement: giaa036_GIGA-D-19-00354_Revision_1 [file giaa036_giga-d-19-00354_revision_1.pdf]

GigaScience

Chromosome-level reference genome of the jellyfish Rhopilema esculentum

--Manuscript Draft--

**Manuscript Number:**	GIGA-D-19-00354R1
**Full Title:**	Chromosome-level reference genome of the jellyfish Rhopilema esculentum
**Article Type:**	Data Note
**Funding Information:**	National Natural Science Foundation of China (31302173) — Mr. Yunfeng Li<br>National Natural Science Foundation of China (31602156) — Dr. Meilin Tian<br>National Natural Science Foundation of China (31602155) — Dr. Lei Gao<br>the Science and Technology Program of Liaoning Province, China (2013203001) — Dr. Zunchun Zhou<br>the Natural Science Foundation of Liaoning Province, China (20180551158) — Mr. Yunfeng Li<br>the Scientific Research Program of Ocean and Fisheries Administration of Liaoning Province, China (201827) — Mr. Yunfeng Li<br>Liaoning Science Public Welfare Research Fund Project (20180015) — Dr. Yulong Li

Funder	Recipient
National Natural Science Foundation of China (31302173)	Mr. Yunfeng Li
National Natural Science Foundation of China (31602156)	Dr. Meilin Tian
National Natural Science Foundation of China (31602155)	Dr. Lei Gao
the Science and Technology Program of Liaoning Province, China (2013203001)	Dr. Zunchun Zhou
the Natural Science Foundation of Liaoning Province, China (20180551158)	Mr. Yunfeng Li
the Scientific Research Program of Ocean and Fisheries Administration of Liaoning Province, China (201827)	Mr. Yunfeng Li
Liaoning Science Public Welfare Research Fund Project (20180015)	Dr. Yulong Li

Abstract:

Background

Jellyfish belong to the phylum Cnidaria, which occupies an important phylogenetic location in the early-branching Metazoa lineages. The jellyfish Rhopilema esculentum is an important fishery resource in China. However, the genome resource of R. esculentum has not been reported to date.

Findings

In this study, we constructed a chromosome-level genome assembly of R. esculentum using Pacific Biosciences, Illumina and Hi-C sequencing technologies. The final genome assembly was approximately 275.42 Mb, with a contig N50 length of 1.13 Mb. Using Hi-C technology to identify the contacts among contigs, 260.17 Mb (94.46%) of the assembled genome were anchored onto 21 pseudochromosomes with a scaffold N50 of 12.97 Mb. We identified 17,219 protein-coding genes, with an average CDS length of 1,575 bp. The genome-wide phylogenetic analysis indicated that R. esculentum might have evolved slower than the other scyphozoan species used in this study. In addition, 127 toxin-like genes were identified, and one toxin-related “hub” was found by a genomic survey.

Conclusions

We have generated a chromosome-level genome assembly of R. esculentum that could provide a valuable genomic background for studying the biology and pharmacology of jellyfish, as well as the evolutionary history of Cnidaria.

**Corresponding Author:**	Zunchun Zhou<br><br>CHINA
**Corresponding Author Secondary Information:**	
**Corresponding Author's Institution:**	

Powered by Editorial Manager® and ProduXion Manager® from Aries Systems Corporation

**Corresponding Author's Secondary Institution:**	
**First Author:**	Yunfeng Li
**First Author Secondary Information:**	
**Order of Authors:**	Yunfeng Li
	Lei Gao
	Yongjia Pan
	Meilin Tian
	Yulong Li
	Chongbo He
	Ying Dong
	Yamin Sun
	Zunchun Zhou
**Order of Authors Secondary Information:**	
**Response to Reviewers:**	Dear Dr. Hans Zauner and reviewers,<br><br>We have carefully read through the comments and made proper revisions according to the reviewers' valuable advice. We detailed our replies in the point-by-point response file. In addition, we also modified some other formatting, grammar and spelling mistakes. We greatly appreciate you and the reviewers for your time and efforts to improve our manuscript. We hope that the revised submission can now be accepted for publication in GigaScience.<br><br>Reviewer #1<br>This is a good paper describing a very exciting/important dataset. The assembly is of high quality and the annotations and analyses are thorough. These data will make a great contribution to several fields of biology, especially evolutionary biology, and venom-omics. The scope of the paper is appropriate for a data note. The figures look nice (especially Figure 1!). The venom analyses at the end of the paper provides a great example of how these data can be used to make impactful discoveries. There are grammar issues throughout the paper (not terrible but substantial). The paper would benefit greatly from being edited by an editor whose first language is English, with an eye towards grammar. The paper would be more clear if passive voice was converted to active voice throughout. In that way it would be clear that the authors performed the work being described (it is often ambiguous). For example on line 178: "A set of 32,138 gene families were eventually identified" would be more clear as "We identified a set of 32,138 gene families..."<br>Below are specific comments with line numbers. NOTE: I received access to supplemental data after my initial review. A few of these comments may be addressed by current supplemental data.<br><br>1. Methods and results are intermingled throughout the paper. This serves to make the methods difficult to repeat and the results hard to follow. I highly recommend separating them. Regarding the methods, it is very important that command lines and information associated with each analyses be made available in the supplemental data. The current 'readme.txt' file is minimal and it is not possible to reconstruct the analyses from this document. Transcriptome assembly should be made made available in the supporting data. The availability of assembled datasets should be mentioned in the section titled "Availability of supporting data."<br>Response: This was done according to the writer's instruction of Data Note in Giga Science. We have tried our best to make the expression clear in the revised manuscript. In addition, the transcriptome assembly has been uploaded and the file name is "trinity.fasta.gz" and the corresponding description has been added in Availability of supporting data section.<br><br>2. Line 17: "Jellyfish belongs" .

-- should be "Jellyfish belong"
Response: This has been corrected in the revised manuscript.

3. Line 17: "occupies an important phylogenetic location together with Porifera as one of the earliest branching Metazoa lineages after their divergence with Ctenophora."
-- This is confusing. Jellyfish did not diverge "with" Ctenophora. They also did not diverge "from" Ctenophora, which I assume was the intended meaning (the diverged from the last common ancestor of ctenophora and jellyfish). It feels strange to refer to "Porifera" especially if "Placozoa" is not also referred to. This first sentence has a lot of problems and could be deleted.
Response: This sentence has been rewritten as “which occupies an important phylogenetic location in the early-branching Metazoa lineages” in the revised manuscript.

4. Line 19: "The jellyfish Rhopilema esculentum is important fishery resource in China."
-- This sentence is not grammatically correct. The first two sentences suffer from grammar issues. I will skip most grammar issues moving forward, but the issues are throughout the manuscript and should be addressed.
Response: This sentence has been rewritten as “The jellyfish Rhopilema esculentum is an important fishery resource in China” in the revised manuscript. All other issues throughout the manuscript have been checked and addressed.

5. Line 29: "that R. esculentum was less evolved than other species in Scyphozoa."
-- It would be more appropriate to say "that there are fewer inferred mutations in the R. esculentum genome as compared to other available scyphozoan genomes and therefore that it might be evolving slower than those of other scyphozoans."
Response: We have revised the abstract and discussion according to your suggestion.

6. Line 39: "Jellyfish belongs..."
-- See comments on the first sentence (line 17) of the abstract above.
Response: Done

7. Line 43: "abundant fishery animals in China"
-- Not clear if this refers to the abundance of these animals in China seas or in China fish markets.
Response: This sentence means R. esculentum is one of the most abundant fishery animals in the seas. The corresponding correction has been made in the context.

8. Line 50: "122,800,000 dollars/year of the profit in China"
-- US dollars?
Response: We have reconfirmed the profit value of R. esculentum in reference [5] and expressed clearly.

9. Line 56: "mainly concerning the increasing jellyfish blooms throughout the world"
-- I assume this is referring to the motivation of these scyphozoan genome projects. As one of the authors of [9], I disagree with this being the motivation. This phrase could be deleted.
Response: Done. It has been deleted from the revised manuscript.

10. Line 73: "For PacBio library construction, genomic DNA of R. esculentum"
-- it should be specified whether this is a different sample or the same sample as the one used for Illumina gDNA sequencing.
Response: The sample used to conduct PacBio library construction was the same one used in next-generation sequencing. The corresponding description has been added in the revised manuscript.

11. Line 82: "We adapted a method using Jellyfish software based"
-- How does this estimation compare to the standard method implemented in GenomeScope (http://qb.cshl.edu/genomescope/)? The details of the estimating method used in this publicaiton should be provided as code or a series of command lines (in a supplement or public repository like GitHub). Or just report the results from GenomeScope, which have been reported in more than 20 other papers.
Response: The further estimation of genome size has been added in the revised

manuscript using GenomeScope. The command lines of Jellyfish were provided in the next question.

12. The methods appear thorough, however repeating these analyses in full would be impossible without further information. In order to make the work repeatable, please include ALL command lines (including infiles or URLs where infiles can be found) and software versions in a supplemental document. There is an excellent example in the supplement linked here:
https://academic.oup.com/mbe/article/35/2/486/4644721#113627427. All analyses including the following should be included in this document.
Response: All command lines used in this study are as follows. They have been also uploaded in GigaDB.
1) Genome estimation analysis
jellyfish count -m 17 -s 800M -t 48 -o kmer.hash -c 20 -C read1.fastq read2.fastq
jellyfish histo -l 1 -h 12000000000 -t 48 kmer.hash > kmer.hash.freq
jellyfish stats $3.hash > kmer.hash.stats
Rscript /gpfs/home/dongwei/biosoft/genomescope-master/genomescope.R kmer.hash.freq 17 150 kmer 1000

2) wtdbg2 analyses (Line 98)
wtdbg2 -i reads.fasta -fo haizhe -t 60 -x sq -g 300m
wtpoa-cns -t 60 -i haizhe.ctg.lay.gz -fo haizhe.raw.fa

3) Quiver analyses (Line 99)
pbalign --nproc 80 input_bam.fofn haizhe.raw.fa pbalign.bam
samtools faidx haizhe.raw.fasta
variantCaller -j8 --algorithm=arrow -r haizhe.raw.fa -o haizhe.cns.fasta pbalign.bam

4) Illumina clean genome data by in-house script (Line 101; This script should be made available -- for example in a software repo like Github)
bwa index haizhe.cns.fa -p haizhe
bwa mem -t 80 -B 1 -O 1 -L 0,0 -k 17 -U 7 haizhe 1.fq 2.fq 2>bwa.log | samtools view -S -@ 60 -b - -o out.bam
samtools sort -@ 60 out.bam out.sorted
samtools flagstat out.sorted.bam > mapsum
bcftools mpileup -Q 0 -e 10 -f haizhe.cns.fa out.sorted.bam 2>bcftools.log |grep -v '#' |awk '$5!="<*>"' > out.raw.vcf
python call_snp.py out.raw.vcf > snp_indel.xls
python changeSNP.py haizhe.cns.fa snp_indel.xls > haizhe.correct.fa

5) Genome assembly that led to 760 contigs and N50 of 1.13Mb (line 102)
wtdbg2 -i reads.fasta -fo haizhe -t 60 -x sq -g 300m
wtpoa-cns -t 60 -i haizhe.ctg.lay.gz -fo haizhe.raw.fa

6) RepeatModeler (Line 104)
/bins/RepeatModeler/BuildDatabase -engine wublast -name Rhopilema Rhopilema.fasta;
/bins/RepeatModeler/RepeatModeler -engine wublast -database Rhopilema -pa 20;

7) RepeatMasker (Line 104)
/bins/RepeatModeler/BuildDatabase -engine wublast -name Rhopilema Rhopilema.fasta;
/bins/RepeatModeler/RepeatModeler -engine wublast -database Rhopilema -pa 20;

8) Augustus (Line 113)
augustus --genemodel=complete --gff3=on --species=haizhe haizhe.fasta > augustus.gff

9) GlimmerHMM (Line 114)
GlimmerHMM/bin/glimmerhmm_linux fasta.file -d ./arabidopsis/

10) SNAP15 (Line 114)
SNAP_train.pl <trained_file> <fasta_out> <trainSet_out>

Powered by Editorial Manager® and ProduXion Manager® from Aries Systems Corporation

	11) Genewise (Line 119)<br>genewise protein.fasta dna.fasta -both -gff<br><br>12) TopHat (sidenote: spelled wrong on page 123)<br>tophat -m 1 -o map --solexa-quals -a 5 --segment-length 20 --num-threads 20 genome.fasta RNAseq_R1.fq RNAseq_R2.fq 2 >out.mapping.log<br><br>13) Cufflinks (Line 124)<br>cufflinks -o transcripts.gtf -p 30 out.sort.bam<br><br>14) EvidenceModeler (Line 126)<br>perl evm.pl haizhe.fasta evm.gene.predict.gff3 evm.transcript.alignment.gff3 evm.homology.predict.gff3 haizhe_20180720-weight.txt<br><br>15) bwa (line 137)<br>bwa index haizhe.correct.fa -p haizhe<br>bwa mem -B 3 -O 3,3 -L 0,0 -t 80 haizhe 1.fq 2.fq 2>bwa.log | samtools view -S -@ 60 -b - -o out.bam<br>samtools sort -@ 60 out.bam out.sorted<br>samtools depth out.sorted.bam > out.sorted.depth<br><br>16) BUSCO (Line 138)<br>pythonrun_BUSCO.py -i haizhe_20180723.evm_final.rechoose.all.faa -o haizhe_euk -l /bins/busco/dataset/euk/ -m proteins -c 40<br><br>17) HiCPlotter (Line 158)<br>HiCPlotter.py -f matrixfile -bed bedfile -r window -tri 1 -wg 1 -chr chromosomename -o outfileprefix -n figuretitle<br><br>18) OrthoMCL (Line 173)<br>orthomclFilterFasta my_orthomcl 10 20<br>orthomclInstallSchema config<br>orthomclBlastParser goodProteins.bsp my_orthomcl >> similarSequences.txt<br>orthomclLoadBlast config similarSequences.txt<br>orthomclPairs config orthomcl_pairs.log cleanup=yes<br>orthomclDumpPairsFiles config<br>orthomclMclToGroups Cluster 1000 < mclOutput > groups.txt<br><br>19) BLASTP (line175: I assume this is a step in OrthoMCL)<br>blastall -p blastp -d goodProteins.fasta -i goodProteins.fasta -F F -m8 -a 30 -e 1e-5 -o goodProteins.bsp<br><br>20) MCL (line177: assume this is a step in OrthoMCL)<br>mcl mclInput --abc -I 1.5 -o mclOutput<br><br>21) MUSCLE (line 181)<br>muscle -in gene.ffn -out gene.ffn.mus<br><br>22) RAxML (line 183)<br>perl Fasta2Phylip.pl only_snp.fasta phylip<br>raxmlHPC-PTHREADS -s phylip -n out.nwk -m PROTGAMMAJTT -p 12345 -T 20 &<br>raxmlHPC-PTHREADS -s phylip -n out_boot.nwk -m PROTGAMMAJTT -p 12345 -T 20 -b 12345 -# 100<br>raxmlHPC-PTHREADS -f b -s phylip -m PROTGAMMAJTT -n haizhe_20170801.nwk -z RAxML_bootstrap.out_boot.nwk -t RAxML_bestTree.out.nwk -T 3<br><br>23) CAFE (line 188)<br>perl cafe_pipe.pl geneFamily.tab time_tree.nwk --output cafe --filter<br><br>24) ParaAT and KaKs_Calculator (Line 211)<br>ParaAT.pl -homolog Orthogroups.tsv -aminoacid AA.fasta -nuc CDS.fasta -processor proc.txt -output kaks -format axt -kaks

Powered by Editorial Manager® and ProduXion Manager® from Aries Systems Corporation

13. Line 106: "the low complexity or simple repeats were not masked"
-- What WAS masked?
Response: The interspersed repeats (SINEs/LINEs/LTR elements/DNA elements/Unclassified) were masked and the detailed description has been added in the revised manuscript as follows "To ensure the integrity of the genes in subsequent analysis, all repeat sequences, except for the low complexity or simple repeats, were masked in this analysis, because some of the low complexity or simple repeats could be found in the genes".

14. Line 118: "aligned to the assembled genome"
-- how was it aligned? Provide software, version, and command lines.
Response: Genewise (version 2.2.0) was used to align and generate gene structures based on the homology alignments. The corresponding sentence has been corrected as "Genewise (version 2.2.0) [17, 18] was used to generate the gene structures based on the homology alignments".

15. Line 135: Quality assessment
-- It is impossible to judge the quality of the cufflinks transcriptome and the predicted gene models from this paper. Please provide BUSCO scores for both.
Response: Yes, we are pleased to provide BUSCO scores. Please see Supplementary Table S5.

16. Line 129: "All the gene sequences were mapped"
-- how were they mapped? Provide software, version, and command lines.
Response: Done. We have revised this sentence to "All the gene sequences were searched using BLASTP with an E-value of 1e-5 against several public databases, including NR [22], GO (Supplementary Fig. S3) [23], Swiss [24], KOG (Supplementary Fig. S4) [25] and KEGG [26], to obtain the functional annotation".

17. Line 132: "could be mapped to at least one database"
-- what was criteria used for successful mapping?
Response: The criteria used for mapping is E-value<1e-5 and this description has been added in the context.

18. Line 138: "The percentage of aligned reads was estimated to be 99.81%."
-- what was criteria used for successful mapping?
Response: The criteria used for mapping is "bwa mem -t 80 -B 1 -O 1 -L 0,0 -k 17 -U 7".

19. Line 141: "C: 97.0% [S: 92.1%, D: 5.0%], F: 1.7%, M: 1.3%, n: 303, wherein C, S, D, F,"
-- this would be more clear as a table with the important stats mentioned in the text.
Response: The BUSCO data was provided in the Supplementary Table S4.

20. Line 151: "essentially as previously described"
-- "essentially" implies that there were variations from the previously described method. Any deviations from the previously described study should be provided.
Response: The method used in this study is exactly the same as described in reference 28 and the word "essentially" has been removed from the context.

21. Line 152: "MboI was used as the restriction enzyme"
-- there needs to be more information here. For example. "The DNA was digested by MboI for X hours at X°C."
Response: The DNA was digested overnight (12h) by 200U of the restriction enzyme MboI at 37 oC with shaking. The information has been inserted in the revised manuscript.

22. Line 158: "Finally, 260.17 Mb (94.46%) of the assemblies were anchored onto 21 pseudochromosomes"
-- Is this the output of HiCPlotter or a different unspecified step. It is not clear.
Response: This is the output of 3D-DNA. To make it clearer, we have revised this sentence to "The 3D-DNA was used to assign the order and orientation of each group [30]. The contact maps were plotted using HiCPlotter software [31]. Finally, 260.17 Mb (94.46%) of the assembly was anchored onto 21 pseudochromosomes, which was in

agreement with the Karyotype (2n=42) of R. esculentum [32] (Fig. 2, Supplementary Fig. S6 and Supplementary Table S6). This chromosome-level assembly resulted in a scaffold N50 of 12.97Mb".

23. Line 172: "All data of the other 12 species were obtained from Ensembl or NCBI"
-- A supplemental table with sources of all data (including accessions or URLs) should be supplied.
Response: Done. Please see Supplementary Table S7.

24. Line 180: "selected for further alignment using MUSCLE"
-- This sounds like MUSCLE was used to select the orthogroups, which doesn't make sense. This should be clarified.
Response: To make it clearer, we have revised this sentence to "A total of 335 selected single-copy orthologous genes were aligned using MUSCLE (v3.6) [35]".

25. Line 182: "alignment by an in-house Perl script."
-- This script should be made publicly available either as a text file in the supplement or on a code repository like GitHub.
Response: Done. We have made publicly available as text files in the supplement on GigaDB.

26. Line 182: " maximum likelihood phylogeny was reconstructed using RAxML"
-- There is no information regarding model, partitioning, etc. If partitioning was used, the partition file should be supplied. The alignment should be supplied as a textfile. Output treefiles should be made available as text files in the supplement.
Response: We have made publicly available as text files in the supplement on GigaDB. It is as follows.

```
perl Fasta2Phylip.pl only_snp.fasta phylip
raxmlHPC-PTHREADS -s phylip -n out.nwk -m PROTGAMMAJTT -p 12345 -T 20 &
raxmlHPC-PTHREADS -s phylip -n out_boot.nwk -m PROTGAMMAJTT -p 12345 -T 20
-b 12345 -# 100
raxmlHPC-PTHREADS -f b -s phylip -m PROTGAMMAJTT -n haizhe_20170801.nwk -z
RAxML_bootstrap.out_boot.nwk -t RAxML_bestTree.out.nwk -T 3
```

27. Line 183: "The results supported the view that R. esculentum and H. vulgaris are sister groups."
-- This is wrong. Figure 4 has A. aurita as sister to R. esculentum
Response: The results supported the view that R. esculentum together with A. aurita and H. vulgaris are sister groups. The expression has been revised.

28. Line 189: "27 and 27 gene families"
-- typo?
Response: To avoid misleading, the sentence has been re-written as "Twenty-seven gene families were found to be significantly expanded and another 27 gene families were found to be significantly contracted in R. esculentum ($P < 0.05$)".

29. Line 200: "244 unique gene families that could be annotated in NR"
-- How could they be annotated? What software? What cutoff?
Response: Blastp was used for gene annotation with a cutoff E-value of 1e-5 and the corresponding description has been added in the context.

30. Line 202: "were annotated with the proteins of the species in Anthozoa"
-- How were they annotated?
Response: The sentence has been re-written as "It was surprising that more than half of those (136 unique gene families) were best annotated with Anthozoa species in NR database".

31. Line 203: "This was also supported by the results of phylogenetic analysis among the 13 species"
-- what was supported. I don't understand.
Response: Considering the problem you mentioned, this sentence has been corrected as "This result implied that some gene families that were possessed by the last common ancestor of Anthozoans and Scyphozoans were kept by the Anthozoan species and R. esculentum, but were lost in A. aurita, N. nomurai, C. xamachana and

H. vulgaris. This was also supported by the phylogenetic analysis of the 13 species, in which R. esculentum was found to exhibit fewer gene gains (331) and fewer gene losses (294) compared with H. vulgaris (513 gains and 666 losses) and A. aurita (696 gains and 962 losses) (Fig. 4)”.

32. Line 204: "Compared to H. vulgaris and Aurelia, R. esculentum had fewer gene gains (331) and fewer gene losses (294)."
-- Sentence should include the number of gains and losses in H. vulgaris and Aurelia.
Response: Combining the problem pointed out in line 203 of the original manuscript, this sentence has been corrected as “This result implied that some gene families, those were possessed by the last common ancestor of Anthozoa and Scyphozoa, were kept by Anthozoa species and R. esculentum, but were lost in A. aurita, N. nomurai, C. xamachana and H. vulgaris. This was also supported by the phylogenetic analysis of the 13 species, in which R. esculentum was found to exhibit fewer gene gains (331) and fewer gene losses (294) compared with H. vulgaris (513 gains and 666 losses) and A. aurita (696 gains and 962 losses) (Fig. 4)”.

33. Line 206: "were kept by Anthozoa species and R. esculentum, but were lost in other Scyphozoa species."
-- the entire quote above would be clearer and more accurate if replaced by "were lost in Aurelia and H. vulgaris."
Response: Thanks for your advice. The sentence has been revised as you suggested.

34. Line 208: "To further explore this hypothesis..."
-- What hypothesis? There has not been one stated.
Response: Please see the next respondence.

35. Line 208: "There were 7542, 7864, 7611 and 6141 orthogroups found in Re-Hv, Aa-Hv, Nn-Hv and Cx-Hv, 10213 respectively."
-- How were paralogs treated? How were sequences aligned? This very rough positive selection analysis is not convincing. I don't trust these results to tell anything (even though it is unclear what the results are supposed to say)
Response: The sequences were analyzed using OrthoMCL - ParaAT - KaKs_Calculator pipeline. The results are supposed to show that R. esculentum had less positive selection genes than other Scyphozoa species after the split of jellyfish and Hydrozoa. The distant phylogenetical species were selected because there were very few species that have genomic resources available. As you suggested, the distant phylogenetical species may lead to rough results. Thus, we deleted this analysis and the corresponding results.

36. Line 219: "Jellyfish, one of the main subgroups of Cnidaria, is one of the oldest extant lineages of venomous animals"
-- This sentence implies that Jellyfish are older than other cnidarian groups. I think the authors were trying to say that cnidarians are older than other venomous animals. However, even this is problematic since the lineage leading to snakes or cone snails (for example) are as old as the lineage leading to cnidarians assuming we are measuring the age from the ancestor (e.g. from the last common ancestor of all animals). This sentence could be deleted.
Response: The corresponding sentence has been revised.

37. Line 235: "Second, according to the gene annotations of NR, Uniprot and Tox-Prot, the genes annotated consistently were chosen as the toxin-like genes."
-- This sentence is not clear. Active voice may help. Also, if there is explicit criteria being used, it should be stated clearly.
Response: It has been revised as follows: “In step 2, according to the best hits of gene annotations of NR, Uniprot and Tox-Prot, the genes that were consistently annotated as toxin-like genes were then chosen”.

38. Line 237: "Third, to make the pool of venom-related genes more complete, we screened all the genes predicted in the genome manually by their annotation."
-- This too is unclear. Also, manual curation is difficult to repeat, but if it must be manual, than detailed criteria should be supplied. It would be much better to convert the manual curation criteria into a program and provide this program as supplemental

material. Either way, it needs to be repeatable.
Response: The manuscript has been revised as follows: “In step 3, to make the pool of venom-related genes more complete, we checked all the gene annotations of the jellyfish and picked out the genes where the annotations were consistent with the annotations in the database of Tox-Prot and were not identified in the first two steps. These genes were also considered as toxin-like genes”.

39. Line 253: "were observed in the tentacles of scyphozoan and cubozoan species "
-- Assuming these observations were not made as part of this study, it would be more clear to say "have been observed in the tentacles of scyphozoan and cubozoan species"
Response: This description is indeed not part of this study and the corresponding presentation has been corrected as you suggested.

40. Line 256" "suggesting their important roles during evolution."
-- It is not clear why this result suggests "important roles during evolution." This should be clarified or removed.
Response: The presentation has been deleted for the context.

41. Line 259: "were highly abundant in cubozoan venoms"
-- Assuming these observations were not made as part of this study, it would be more clear to say "have been observed in high abundance in cubozoan venoms"
Response: The sentence has been re-written as you suggested.

42. Line 259: "were also reported"
-- "have been reported"
Response: The mistake has been revised in the context.

43. Line 265: "Three new toxin-like genes that have not been previously reported in jellyfish were..."
-- Prothrombin was reported in Cassiopea in Ohdera et al. 2019 (Table S4 -- line 73).
Response: The corresponding result and description have been removed from the manuscript.

44. Line 274: "was mainly identified in the honeybee"
-- grammar issue makes this confusing.
Response: This sentence has been reformed as “It was mostly found in honeybee” for better understanding.

45. Line 275: "The new toxin-like genes identified in this study will provide insight into the complex composition of jellyfish venom."
-- as written this sentence is kind of a throwaway. It would be better to mention that: the discovery of these toxin-coding genes in R. esculenum adds to a growing understanding of the composition of jellyfish venoms.
Response: We have revised this sentence as you suggested.

46. Line 281: "The different composition of venom may account for the different symptoms after sting."
-- what are the different symptoms?
Response: According to the present analysis, stinging by Rhopilema hispidum would cause immediate strong burning sensation increasing for 3~4 hours and disappeared in the following few days. Stinging by R. esculentum would cause strong pruritus and instant mild pain lasting for about 30 minutes. When touched by Nemopilema nomurai tentacles, it would result in a very painful sting with a strong burning sensation. The reference for these has been cited in the revised manuscript: “Kawahara M, Uye S, Burnett J et al. Stings of edible jellyfish (Rhopilema hispidum, Rhopilema esculentum and Nemopilema nomurai) in Japanese waters. Toxicon 2006;48(6):713-6”. A brief difference illustration has been added in the context.

47. Line 291: "It was also reported that neighboring genes tend to be co-expressed rather than expressed by chance"
-- This is misleading. This is an Arabidopsis paper. At least find a paper that shows this in animals and mention explicitly that in animals sometimes proximity can relate to co-expression.

Response: The related studies have been cited in the manuscript.

48. Line 294: "Further studies are needed to clarify its specific functions."
-- This is not a great way to end the paper. Why not add a paragraph conclusion saying how these new data can be used in the future. etc. You could find many examples from other giganotes.
Response: A new ending paragraph has been added in the context.

49. Table 3: "The green and yellow boxes indicate the identified and unidentified toxin-like genes respectively"
-- "unidentified toxin-like genes" is confusing. It would be more clear to make yellow boxes white (or unfilled) and state that "green boxes represent potential toxin-coding genes."
Response: The boxes and the table illustration of Table 3 have been revised as you suggested.

50. Figure 1: "Picture of a jellyfish R. esculentum captured from Yingkou, Liaoning Province, China."
-- "Photograph of Rhopilema esculentum collected from Yingkou, Liaoning Province, China." Either include approximate size or a scale bar.
-- Also, Figure 1 is not (but should be) referenced in the main text.
Response: Figure 1 has been referenced in the revised manuscript.

Reviewer #2
This manuscript presents a chromosome-level genome assembly of the edible scyphozoan jellyfish Rhopilema esculentum, combining Illumina, PacBio and HiC technologies. As there is currently no published chromosome-level cnidarian genome assembly, this study represents certainly a very interesting resource for future studies.

Major comments:

1. Several relevant methodological details are missing, and would need to be provided: The origin of the biological material from which the DNA and RNA samples were prepared is not specified. Did they derive from wild animals or cultured animals? Which geographical origin and/or which strain(s)? How were the animals collected? Were the samples pooled, and if so, how many individuals/piece of tissues were used for each extraction?
Response: Some of the details were included in the NCBI database. The experimental individuals are all cultured animals. All the samples were collected from Yingkou Modern Fishery Technology Company, Yingkou city, Liaoning province, China. The specific geographical origin is N40°29′00.98″, E122°13′38.86″, with the altitude of -13 meter. One individual was used for genomic sample, and one another individual was used for Hi-C analysis. For transcriptomic analysis, a total of 60 individuals of four development periods were collected. Five individuals were pooled and three replicates were set for each development period analysis. This has been revised in the manuscript.

2. Which procedures were performed in order to avoid contamination? Were the organisms starved prior to extraction? Were they treated with antibiotics?
Response: The experimental individuals were starved for two days prior to extraction, and sterile water was used to treat the tissue sample to avoid contamination. Antibiotic was not used in this study. This has been revised in the manuscript.

3. For RNAseq experiments - please provide details about the sequencing. Was it pair-ended? How many replicates? This information should be provided in the main text.
Response: This study was carried out by PE-250 sequencing utilizing Illummina HiSeq2500 platform. Three replications were used for each group. The detailed information has been added in the manuscript.

4. The phylogenetic methods used to produce figure 4 should be provided. Which model was used for the RaXML analyses? How was done the fossil calibration? Which method was used to calibrate the tree?
Response: PROTGAMMAJTT model was used for RaXML analyses. The divergence

times of Mnemiopsis leidyi vs. Aurelia aurita, Strongylocentrotus purpuratus vs. Aurelia aurita, and Danio rerio vs. Nematostella vectensis were retrieved from the time tree (http://www.timetree.org) and used as the fossil calibration. R8s was used to calculate the divergence time of each node in phylogenetic tree. These have been added in the manuscript. The command lines are as follows.

RaXML command：
perl Fasta2Phylip.pl only_snp.fasta phylip
raxmlHPC-PTHREADS -s phylip -n out.nwk -m PROTGAMMAJTT -p 12345 -T 20 &
raxmlHPC-PTHREADS -s phylip -n out_boot.nwk -m PROTGAMMAJTT -p 12345 -T 20 -b 12345 -# 100
raxmlHPC-PTHREADS -f b -s phylip -m PROTGAMMAJTT -n haizhe_20170801.nwk -z RAxML_bootstrap.out_boot.nwk -t RAxML_bestTree.out.nwk -T 3
fossil calibration：
MRCA AA Mnemiopsis_leidyi Aurelia_aurita;
MRCA BB Strongylocentrotus_purpuratus Aurelia_aurita;
MRCA CC Danio_rerio Nematostella_vectensis;
CONSTRAIN taxon=AA min_age=698 max_age=1047;
CONSTRAIN taxon=BB min_age=617 max_age=902;
CONSTRAIN taxon=CC min_age=641.8 max_age=1250;
Café command：
perl cafe_pipe.pl geneFamily.tab time_tree.nwk --output cafe --filter

5. All the scripts mentioned in the main text should be provided as supplementary information (e.g. L102, L182).
Response: All scripts have been made available on GigaDB.

6. The statement that R. esculentum evolved less than other scyphozoan species is not very convincing. This would need to be studied in deeper detail with a greater number of compared species. I would suggest removing this statement from both main text and abstract. The lower number of gene gain and loss detected in R. esculentum, compared to other schyphozoans, relies on the comparison of too few species. I would also suggest entirely removing the part on positive selection in R. esculentum (L208-L216), since the species used for comparison (Aurelia aurita and Hydra vulgaris) are too distant phylogenetically for an accurate estimate of the selective forces.
Response: As you suggested, our analysis was only focus on several Scyphozoa species, not all Scyphozoa species. Thus, we revised our conclusion that R. esculentum might have evolved slower than the Scyphozoa species analyzed in our study. In addition, the distant phylogenetical species were selected because there were very few species that have genomic resources available. As you suggested, the distant phylogenetical species may lead to rough results. Thus, we deleted this analysis and the corresponding results.

Other comments:
7. I would suggest removing the parentheses in the title.
Response: The parentheses have been deleted from the title.

8. L17-18: The phylogenetic position of ctenophore is still highly debated. I would suggest removing "after their divergence with Ctenophora". Same comment for L40-41.
Response: The debatable description has been deleted from the context.

9. L19: add 'an': "is an important".
Response: Done.

10. L23-24. The sentence "A total… respectively." seems to me unnecessary for an abstract. I would suggest removing it.
Response: Done.

11. L32: replace "we first finished" with "we generated a"
Response: Done.

12. L41: add the year of species description "1891" after "Kishinouye".
Response: Done.

Powered by Editorial Manager® and ProduXion Manager® from Aries Systems Corporation

	13. L42: lowercase for "Class" - also L198.<br>Response: Done.<br><br>14. L47: rephrase "their harmfulness to industry and the community in blooms".<br>Response: The sentence has re-written as “In contrast to many other jellyfish species that have drawn public attention because of their harmful blooms [4], the population of R. esculentum has declined in recent years as a result of overfishing [2]” for better understanding.<br><br>15. L48: replace "for" with "because of" and provide a reference in support of that claim.<br>Response: The word has been revised in the main context and the reference has been added.<br><br>16. L54: replace "Aurelia and Aurelia aurita" with "Aurelia aurita".<br>Response: Done.<br><br>17. L53-56. Other recent releases of genome assembly of non-scyphozoan medusozoans - cubozoan (Morbakka virulenta, and Alatina alata), hydrozoan (Clytia hemisphaerica), staurozoan (Calvadosia cruxmelitensis) could also be mentioned here.<br>Response: New citation has been made in the manuscript and the detailed information has been added both in the context and references.<br><br>18. L56. I would delete "mainly concerning the increasing jellyfish blooms throughout the world" as the recent publications of scyphozoan genome were only marginally addressing this point.<br>Response: This inappropriate description has been removed from the manuscript.<br><br>19. Rhopilema esculentum is often referenced in the text simply as "jellyfish". To avoid confusion, I would replace "jellyfish" with "R. esculentum" when appropriate. e.g.: L148, L161, L164.<br>Response: Done.<br><br>20. L151. "were performed essentially as previously described [28]." Please detail the difference with the published protocol, if any.<br>Response: The protocol used in this study is exactly same as described in reference 28. To make it clear, the word “essentially” has been deleted from the sentence.<br><br>21. L198. Replace "Aurelia" with "A. aurita".<br>Response: Done.<br><br>22. L219. Replace "Jellyfish" with "Medusozoa".<br>Response: Considering of the suggestion from you and the other two reviewers, this sentence has been removed from the manuscript.<br><br>23. L236. "the genes annotated… toxin-like genes". This part of the sentence is unclear, please reformulate.<br>Response: The sentence has been re-written as “In step 2, according to the best hits of gene annotations of NR, Uniprot and Tox-Prot, the genes that were consistently annotated as toxin-like genes were then chosen”.<br><br>24. L258. "Two copies… found". This sentence is unclear, please reformulate.<br>Response: The sentence has been re-organized as “Two copies of “jellyfish toxin”, also called cubozoan-related porins, were also found. The “jellyfish toxins” have been observed in high abundance in cubozoan venoms [48] and they have also been reported in other medusozoans, such as Scyphozoan [47], Hydrozoan [55] and Anthozoan [56]”.<br><br>25. L281. "They are known to…. neurotoxins". A reference needs to be added here.<br>Response: The reference information has been added in the context.<br><br>26. L283. "Some" - how many?<br>Response: Eight toxin-like genes were located closely on contig 521 and the exact number has been added in the manuscript.

27. L285. The term "associated" is too imprecise and should be replaced. Do you mean: found on the same scaffold? If so, how distant?
Response: The sentence has been re-written as “The functions of toxin-like genes in the hub included phospholipase A2 activity, nuclease activity, toxin activity and toxin extrusion”.

28. L290. It is not true that "genes located closely in genome are always involved in related functions and expressed in similar patterns". Please correct or delete the sentence.
Response: The corresponding sentence has been deleted.

29. L292. I would avoid the passive mode and replace "It was suggested" with "We suggest".
Response: Done.

30. L294. Remove "the". "and function of jellyfish" is unclear - reformulate or delete. Please also replace "its" with "their".
Response: To make it clear, “jellyfish” has been replaced by “R. esculentum” and the other two grammar issues have been corrected.

31. Figure 2F: How were calculated those "major interchromosomal relationships"? How to interpret them? Please also provide scale bars for A,C,D,E.
Response: The major interchromosomal relationships was conducted utilizing lastZ with the following command line:
lastz chr1.2bit chr2.2bit --notransition --nogapped --step=40 --format=general:name1,start1,end1,length1,name2,start2,end2,length2,identity > chr1.chr2.lastz
Figure 2 has been replaced by a new version. The scale bars were provided at the left bottom of the figure.

32. Figure 3. Please describe in the figure legend the meaning of the numbers and numbers between brackets shown on the figure.
Response: The detailed description has been added in the figure illustration.

Reviewer #3
In this paper, Li et al. describe the genome of the jellyfish Rhopilema. This is the first chromosome-level assembly of a scyphozoan (true jellyfish), and the data looks of wonderful quality. This paper is worthy of publication in Gigascience, but there are a couple of additional analyses and minor edits that need to be addressed first:

1. A small (but important) point. In several places the authors discuss the Rhopilema genome as being "less evolved" than other scyphozoans (e.g. lines 29-30, 216). All organisms demonstrate lineage-specific patterns of change, and in that regard all species are equally "evolved". The term the authors should be using is "less derived".
Response: Thank you for your suggestion. Combining your and other reviewers’ opinion, “less evolved” has been replaced by “evolved slower than the other scyphozoan species used in this study” to make the description more rigorous.

2. The authors do not include any comparative macro- or microsynteny analyses, which is standard in a genome paper. Given the large number of cnidarian genomes and the quality of their assembly, it would be valuable to see how genomic structure in Rhopilema compares to other cnidarians. I would especially like to see whether synteny analysis supports the less derived nature of the Rhopilema genome, as gene gain/loss analysis appears to.
Response: Actually, we did synteny analyses with other Scyphozoans, and the results were disordered and not adequate for making part of the context. For instance, 6663 synteny loci were found between Rhopilema esculentum and Aurelia aurita, and they were distributed on many different scaffolds. This is mainly because only R. esculentum has chromosome-level genome assembly by now. Instead, we conducted synteny analysis within R. esculentum, and the results were showed in Fig. 2.

3. There is no description on how the authors dated their molecular clock for Figure 4, other than they used "the phylogeny and fossil records" (line 184). The dates near the

base of the tree are outside of the range of most molecular clock analyses (see refs. cited below for detail), which makes the subsequent CAFE analysis suspect. The authors need to provide more information on how their tree is dated and consider methodologies that bring their clock closer in line with more thorough analyses.
Response: PROTGAMMAJTT model was used for RaXML analyses. The divergence times of Mnemiopsis leidyi vs. Aurelia aurita, Strongylocentrotus purpuratus vs. Aurelia aurita, and Danio rerio vs. Nematostella vectensis were retrieved from the time tree (http://www.timetree.org) and used as the fossil calibration. R8s was used to calculate the divergence time of each node in phylogenetic tree. These have been added in the manuscript.

The command lines are as follows.

```
r8s -b -f r8s-contig.txt > r8s_out.txt
perl Fasta2Phylip.pl only_snp.fasta phylip
raxmlHPC-PTHREADS -s phylip -n out.nwk -m PROTGAMMAJTT -p 12345 -T 20 &
raxmlHPC-PTHREADS -s phylip -n out_boot.nwk -m PROTGAMMAJTT -p 12345 -T 20 -b 12345 -# 100
raxmlHPC-PTHREADS -f b -s phylip -m PROTGAMMAJTT -n haizhe_20170801.nwk -z RAxML_bootstrap.out_boot.nwk -t RAxML_bestTree.out.nwk -T 3
fossil calibration：
MRCA AA Mnemiopsis_leidyi Aurelia_aurita;
MRCA BB Strongylocentrotus_purpuratus Aurelia_aurita;
MRCA CC Danio_rerio Nematostella_vectensis;
CONSTRAIN taxon=AA min_age=698 max_age=1047;
CONSTRAIN taxon=BB min_age=617 max_age=902;
CONSTRAIN taxon=CC min_age=641.8 max_age=1250;
Café command：
perl cafe_pipe.pl geneFamily.tab time_tree.nwk --output cafe --filter
```

The content of the config file of r8s is as follows:

```
#NEXUS
Begin trees;
tree STATE_0 = [&R]
(((((((Aurelia_aurita:0.12013130,Rhopilema_esculentum:0.12586633)1.0000:0.17380521,Hydra_vulgaris:0.37848113)1.0000:0.14709140,(Nematostella_vectensis:0.20009030,Stylophora_pistillata:0.19766004)1.0000:0.11813562)1.0000:0.05194067,((Crassostrea_gigas:0.31746486,Daphnia_pulex:0.50980244)0.7200:0.06300857,(Danio_rerio:0.35321013,(Saccoglossus_kowalevskii:0.25047315,Strongylocentrotus_purpuratus:0.33882824)1.0000:0.06040144)0.7900:0.02507859)1.0000:0.08234104)1.0000:0.05214136,Trichoplax_adhaerens:0.54767250)0.9600:0.06896268,Amphimedon_queenslandica:0.56093307)0.0000:0.02270157,Mnemiopsis_leidyi:0.79895767);
End;
BEGIN r8s;
BLFORMAT lengths=persite nsites=52236 ultrametric=no;
COLLAPSE;
MRCA AA Mnemiopsis_leidyi Aurelia_aurita;
MRCA BB Strongylocentrotus_purpuratus Aurelia_aurita;
MRCA CC Danio_rerio Nematostella_vectensis;
CONSTRAIN taxon=AA min_age=698 max_age=1047;
CONSTRAIN taxon=BB min_age=617 max_age=902;
CONSTRAIN taxon=CC min_age=641.8 max_age=1250;
set checkgradient=yes smoothing=100;
divtime method=pl algorithm=tn;
showage;
set penalty=add checkgradient=no;
DIVTIME method=PL fossilconstrained=yes crossv=yes cvstart=0 cvinc=0.50 cvnum=6 algorithm=tn;
DIVTIME method=PL fossilfixed=yes crossv=yes cvstart=0 cvinc=0.50 cvnum=6 algorithm=tn;
describe plot=chrono_description;
END;
```

4. This last suggestion is optional. The authors note a set of venom genes lie close together in the genome and posit that they may be co-expressed. This seems readily

Powered by Editorial Manager® and ProduXion Manager® from Aries Systems Corporation

	testable using the RNA-Seq data that they collected from the different life stages (Table S3). I'm generally confused why the authors collected so much RNA-Seq data (which, as far as I can tell, looks to be of high quality) and only use it for gene modelling. The paper could be much more compelling if that data were leveraged to understand how gene expression changes through the life cycle. But perhaps the authors are saving that for a subsequent paper?<br>Response: The RNA-Seq data we collected will be analyzed together with more sequencing data in the future to address a bigger scientific question.<br><br>Overall this is a well-written manuscript with a quality dataset. I look forward to seeing this paper published once some additional, minor analyses are done.<br><br>References:<br>Dohrmann M, Wörheide G. 2017. Dating early animal evolution using phylogenomic data. Sci Rep 7:3599.<br><br>dos Reis M, Thawornwattana Y, Angelis K, Telford MJ, Donoghue PC, Yang Z. 2015. Uncertainty in the timing of origin of animals and the limits of precision in molecular timescales. Curr Biol 25:2939-2950.<br>Response: We have gone through the references you kindly provided and carefully revised the description of how to date the molecular clock. Please see our response to question 3.<br><br><br>Yours Sincerely,<br><br>Zunchun Zhou, Ph.D.<br>Professor and Director of<br>Liaoning Ocean and Fisheries Science Research Institute
**Additional Information:**	
**Question**	**Response**
Are you submitting this manuscript to a special series or article collection?	No
**Experimental design and statistics**<br><br>Full details of the experimental design and statistical methods used should be given in the Methods section, as detailed in our Minimum Standards Reporting Checklist. Information essential to interpreting the data presented should be made available in the figure legends.<br><br>Have you included all the information requested in your manuscript?	Yes
**Resources**<br><br>A description of all resources used, including antibodies, cell lines, animals and software tools, with enough information to allow them to be uniquely	Yes

identified, should be included in the Methods section. Authors are strongly encouraged to cite Research Resource Identifiers (RRIDs) for antibodies, model organisms and tools, where possible.<br><br>Have you included the information requested as detailed in our Minimum Standards Reporting Checklist?	
**Availability of data and materials**<br><br>All datasets and code on which the conclusions of the paper rely must be either included in your submission or deposited in publicly available repositories (where available and ethically appropriate), referencing such data using a unique identifier in the references and in the “Availability of Data and Materials” section of your manuscript.<br><br>Have you have met the above requirement as detailed in our Minimum Standards Reporting Checklist?	Yes

Manuscript Click here to access/download;Manuscript;Manuscript-0217.docx

Chromosome-level reference genome of the jellyfish *Rhopilema esculentum*

Yunfeng Li[1,†], Lei Gao[1,†], Yongjia Pan[1,†], Meilin Tian[1], Yulong Li[1], Chongbo He[1], Ying Dong[1], Yamin Sun[2,*], Zunchun Zhou[1,*]

[1]Liaoning Ocean and Fisheries Science Research Institute, 50 Heishijiao St., Dalian, Liaoning 116023, China, [2]Tianjin Biochip Corporation, 23 Hongda St., Tianjin 300457, China

[*]**Correspondence address.** Zunchun Zhou, Liaoning Ocean and Fisheries Science Research Institute, 50 Heishijiao St., Dalian, Liaoning 116023, China, E-mail: zunchunz@hotmail.com; Yamin Sun, Tianjin Biochip Corporation, 23 Hongda St., Tianjin 300457, China, E-mail: nksunyamin@aliyun.com.

[†]These authors contributed equally to this work.

Abstract

Background: Jellyfish belong to the phylum Cnidaria, which occupies an important phylogenetic location in the early-branching Metazoa lineages. The jellyfish *Rhopilema esculentum* is an important fishery resource in China. However, the genome resource of *R. esculentum* has not been reported to date. **Findings:** In this study, we constructed a chromosome-level genome assembly of *R. esculentum* using Pacific Biosciences, Illumina and Hi-C sequencing technologies. The final genome assembly was approximately 275.42 Mb, with a contig N50 length of 1.13 Mb. Using Hi-C technology to identify the contacts among contigs, 260.17 Mb (94.46%) of the assembled genome were anchored onto 21 pseudochromosomes with a scaffold N50 of 12.97 Mb. We identified 17,219 protein-coding genes, with an average CDS length of 1,575 bp. The genome-wide phylogenetic analysis indicated that *R. esculentum* might have evolved slower than the other scyphozoan species used in this study. In addition, 127 toxin-like genes were identified, and one toxin-related "hub" was found by a genomic survey. **Conclusions:** We have generated a chromosome-level genome assembly of *R. esculentum* that could provide a valuable genomic background for studying the biology and pharmacology of jellyfish, as well as the evolutionary history of Cnidaria.

Keywords: Jellyfish; *Rhopilema esculentum*; whole-genome sequencing; chromosome-level assembly; toxin-like genes

Data Description

Background

Jellyfish belong to the phylum Cnidaria, which occupies an important phylogenetic location and is one of the earliest branching Metazoa lineages [1]. The jellyfish *Rhopilema esculentum* (Kishinouye, 1891), an edible species in the class Scyphozoa (also named true jellyfish), is widely distributed in the seas around China, Japan and Korea [2], and it is one of the most abundant fishery animals in these locations. *R. esculentum* has been exploited as food for thousands of years and has been gaining more attention recently because of its pharmacological properties [3]. In contrast to many other jellyfish species that have drawn public attention because of their harmful blooms [4], the population of *R. esculentum* has declined in recent years as a result of overfishing [2]. The stock enhancement and aquaculture of *R. esculentum* have been initiated to meet the expanding market demand, which accounts for about 82,280 tons per year, generating US$ 122,800,000 worth of profit per year for the Chinese economy [5]. The lack of genomic resource has limited the phylogenetic study of jellyfish and the investigation of their many specific characteristics. Recently, several genome assemblies have been reported for the medusozoan species, including the moon jellyfish (*Aurelia aurita*) [6, 7], the giant Nomura's jellyfish (*Nemopilema nomurai*) [8], the upside-down jellyfish (*Cassiopea xamachana*) [9], the hydrozoan jellyfish *Clytia hemisphaerica* [10], *Morbakka virulenta* [7], *Alatina alata* [9] and *Calvadosia cruxmelitensis* [9]. However, no chromosome-level reference genome has been reported for the class Scyphozoa, and at present, there is very limited information of

the genome architecture of *R. esculentum*. In the present study, we sequenced the chromosome-level genome of *R. esculentum*, assembled and annotated it to improve our understanding of the evolutionary and pharmacology characteristics of jellyfish.

Sample and sequencing

One cultured *R. esculentum* individual was collected from Yingkou, Liaoning Province, China (Fig. 1). After starving for two days, the epidermis tissue was sampled, and Genomic DNA was extracted using a TIANamp Marine Animal DNA Kits (Tiangen, Beijing, China) and then directly used for the genomic DNA sequencing. The genomic DNA was sheared using a sonication device, and the resulting fragments were used for the construction of short-insert paired-end (PE) library. The short-insert libraries with a size of 500 bp were constructed according to the instruction described in the Illumina library preparation kit. All libraries were sequenced on an Illumina HiSeq2500 platform (Illumina, San Diego, CA, USA) with150-bp paired-ends. In total, approximately 22.6 Gb (80×) of raw data were generated, and 20.03 Gb (71×) of clean data were filtered by FastQC (v0.11.2) (Supplementary Table S1). The genomic DNA used for sequencing was also sheared to yield ~20 kb fragments for the construction of PacBio library. DNA fragments below 7 kb were filtered using BluePipin (Sage Science, MA, USA). The filtered DNA was then converted into the proprietary SMRTbell library using the PacBio DNA Template Preparation Kit. In total, 39.76 Gb (140×) of quality-filtered data with a mean length of 7,196 bp were obtained from the PacBio Sequel platform (Supplementary Table S1).

Genome size and heterozygosity estimation

The distribution of *k*-mer frequency, also known as the *k*-mer spectrum, is widely used for the estimation of genome size. We used a jellyfish software based on a *k*-mer distribution [11] to estimate the genome size with high quality reads above Q20 from short-insert size libraries (500 bp). We obtained a *k*-mer (K=17) depth distribution from the Jellyfish analysis and clearly observed the peak depth from the distribution data. We obtained a genome size estimation of 290 Mb and a heterozygosity of 1.68% by GenomeScope v1.0.0 (Supplementary Fig. S1) [12]. 54.4% of the genome was predicted to be non-repetitive sequences.

Genome assembly and annotation

In the present study, the long reads of PacBio sequencing data was used to solve the high level of heterozygosity, which is one of the main challenges in the assembly of marine invertebrate genomes [13, 14]. The genome assembly was performed using the software wtdbg2 with default parameters (https://github.com/ruanjue/wtdbg2). The assembly sequences were then polished using Quiver (SMRT Analysis v2.3.0) with default parameters. To achieve higher continuity and accuracy for the assembled genome, five rounds of iterative error correction were performed with the Illumina clean genome data using in-house script. Finally, a genome of 275.42 Mb was assembled, with 760 contigs and a contig N50 size of 1.13 Mb (Table 1 and Supplementary Fig. S2).

Both RepeatModeler and RepeatMasker (http://www.repeatmasker.org) were used to perform the *de novo* identification and masking of repeat sequences. To ensure the integrity of the genes in subsequent analysis, all repeat sequences, except for the low complexity or simple repeats, were masked in this analysis, because some of the low complexity or simple repeats could be found in the genes. Finally, 29.23% of the assembled bases (80,495,815 bp) were masked (Supplementary Table S2). Of these, 9.93% could be annotated with known repeat families, and 19.30% were unclassified repeats.

The identification of protein-coding regions and the prediction of genes were performed using a combination of *ab initio* prediction, homology-based prediction, and transcriptome-based prediction methods. The *ab initio* gene prediction was conducted with Augustus (version 2.5.5) [15], GlimmerHMM (version 3.0.1) [16] and SNAP15 [17] to predict the coding genes. For the homology-based prediction, homologous proteins of several Cnidarian species (myxosporean (*Thelohanellus kitauei*), coral (*Stylophora pistillata* and *Orbicella faveolata*), hydrozoan (*Hydra vulgaris*), sea anemone (*Exaiptasia pallida*) and the Cnidaria EST database) were downloaded from NCBI and aligned with our assembled genome. Then, Genewise (version 2.2.0) [18, 19] was used to generate the gene structures based on the homology alignments. For transcriptome-based prediction, 60 individuals of four development periods (scyphistoma, strobili, ephyra and juvenile medusa) were collected. Five individuals were pooled and three replicates were set for each development period analysis. The transcriptome of samples were sequenced using the Iillumina HiSeq2500 platform

(154.6 Gb clean reads, PE-250) (Supplementary Table S3) and mapped the resulting sequences to the genome assembly using TopHat (version 2.0.8) [20]. Cufflinks (version 2.1.1, http://cufflinks.cbcb.umd.edu/) [21] was then used to identify the spliced transcripts in the gene models. All the gene evidence predicted from the above three approaches were integrated by EvidenceModeler (EVM) [22] into a weighted and non-redundant consensus of the gene structures. A total of 17,219 genes, with an average CDS length of 1,575 bp, were finally predicted to be present in the genome of *R. esculentum* (Table 1). All the gene sequences were searched using BLASTP with an *E-value* of $1e^{-5}$ against several public databases, including NR [23], GO (Supplementary Fig. S3) [24], Swiss [25], KOG (Supplementary Fig. S4) [26] and KEGG [27], to obtain the functional annotation. A total of 16,713 genes (97.1%) were successfully mapped to at least one database, and 8,880 genes were annotated in all four databases (*E-value* $< 1e^{-5}$) (Supplementary Fig. S5).

Quality assessment

We first aligned all the Illumina genome reads against the *R. esculentum* assembled genome using the Burrows-Wheeler Aligner (BWA, version 0.7.17) to evaluate the coverage of the genome. The percentage of aligned reads was estimated to be 99.81%. BUSCO (version: 3.0.2) [28] was then used to evaluate the integrity of the genome (Supplementary Table S4). The values of core gene estimation were calculated as follows: C: 97.0% [S: 92.1%, D: 5.0%], F: 1.7%, M: 1.3%, n: 303, where C, S, D, F, M and n indicate complete BUSCOs, complete and single-copy BUSCOs, complete and

duplicated BUSCOs, fragmented BUSCOs, missing BUSCOs and total BUSCO groups searched, respectively (Supplementary Table S5). The results indicated that the assembly covered most of the genetic regions, further confirming the assembly quality of the *R. esculentum* genome.

Pseudochromosome construction

Hi-C experiments were used for the chromosome assembly of *R. esculentum*. The whole-body homogenate of one *R. esculentum* was fixed in 1% (vol/vol) formaldehyde and was then used to prepare the Hi-C libraries. Nuclei extraction and permeabilization, chromatin digestion and proximity-ligation treatments were performed as previously described [29]. The DNA was digested overnight (12 h) with 200 U of the restriction enzyme *Mbo*I at 37 ^{o}C with shaking. The libraries were sequenced on the Illumina X-TEN platform (San Diego, CA, USA) with 2×150 bp reads. They were independently analyzed in the HiC-Pro pipeline (default parameters and LIGATION_SITE = GATC) [30]. A total of 23.96 Gb of trimmed reads were obtained, accounting for around 82-fold coverage of the *R. esculentum* genome. The 3D-DNA was used to assign the order and orientation of each group [31]. The contact maps were plotted using HiCPlotter software [32]. Finally, 260.17 Mb (94.46%) of the assembly was anchored onto 21 pseudochromosomes, which was in agreement with the Karyotype (2n=42) of *R. esculentum* [33] (Fig. 2, Supplementary Fig. S6 and Supplementary Table S6). This chromosome-level assembly resulted in a scaffold N50 of 12.97Mb.

Phylogenetic analysis

To examine the evolutionary relationships among *R. esculentum* and other species, the whole protein sequences of *R. esculentum* and 12 other species (Supplementary Table S7) were analyzed, including species from Ctenophora (ctenophore (*Mnemiopsis leidyi*)), Porifera (demosponge (*Amphimedon queenslandica*)), Placozoa (*Trichoplax adhaerens*), Cnidaria (jellyfish (*R. esculentum* and *A. aurita*), Hydrozoa (*H. vulgaris*), coral (*S. pistillata*), sea anemone (*Nematostella vectensis*)), Protostomia (Lophotrochozoa (pacific oyster (*Crassostrea gigas*)), Ecdysozoa (cladoceran (*Daphnia pulex*))), and Deuterostomia (Echinodermata (sea urchin (*Strongylocentrotus purpuratus*)), Hemichordata (acorn worm (*Saccoglossus kowalevskii*)), Chordata (zebrafish (*Danio rerio*))). All protein models of the 12 other species were obtained from Ensembl or NCBI. Orthologous alignment analysis was performed using OrthoMCL [34]. In detail, the protein-coding genes from the above-sequenced genomes were aligned with each other using the BLASTP program [35]. Similarity in the pair-wise sequence alignments generated by BLASTP was used as distance parameters for gene family clustering by MCL with an inflation value of 1.5.

A set of 32,138 gene families were eventually identified among the other 12 species, of which 2,092 families were present in all 13 species (Fig. 3 and Supplementary Table S8). A total of 335 selected single-copy orthologous genes were aligned using MUSCLE (v3.6) [36] and then concatenated into a single multiple sequence alignment through an in-house Perl script. A maximum likelihood phylogeny was reconstructed using RAxML [37] (Fig. 4). The phylogenic results supported the view that *R.*

esculentum, A. aurita and *H. vulgaris* are sister groups. PROTGAMMAJTT model was used for RaXML analyses [37]. The divergence times of *M. leidyi vs. A. aurita, S. purpuratus vs. A. aurita*, and *D. rerio vs. N. vectensis* were retrieved from the time tree (http://www.timetree.org) and used as the fossil calibration. R8s was used to calculate the divergence time of each node in phylogenetic tree [38]. We dated the divergence time of *R. esculentum* and *H. vulgaris* to approximately 501.71 million years ago (mya), consistent with the previous studies [39]. To compare the jellyfish genomic traits with those of the other 12 species, we performed a comparative genomic analysis for all 13 species using CAFE software (Supplementary Table S9) [40]. Twenty-seven gene families were found to be significantly expanded and another 27 gene families were found to be significantly contracted in *R. esculentum* ($P < 0.05$) (Supplementary Table S10 and Supplementary Table S11). Interestingly, the gene families enriched in the GO category of transmembrane transport were significantly expanded, and the relative GO sub-categories included drug transmembrane transport, drug transmembrane transporter activity, ion transmembrane transporter activity and amino acid transmembrane transporter activity. The action of venom, an important characteristic of jellyfish species, may contribute to gene expansion in the transmembrane transport [41, 42].

A comparative genomic analysis was performed for the four jellyfish species in the class Scyphozoa (including *R. esculentum, A. aurita, N. nomurai* and *C. xamachana*) and *H. vulgaris* (used as outgroup, and to calculate the divergence time). A total of 244 unique gene families were identified in *R. esculentum* using BLASTP with an *E-value*

of 1e^{-5} in NR database. It was surprising that more than half of those (136 unique gene families) were best annotated with Anthozoa species in NR database. It was suggested that the 136 unique gene families were not from the split of *R. esculentum* but from the ancestor of Anthozoa and Scyphozoa. This result implied that some gene families that were possessed by the last common ancestor of Anthozoans and Scyphozoans were kept by the Anthozoan species and *R. esculentum*, but were lost in *A. aurita*, *N. nomurai*, *C. xamachana* and *H. vulgaris*. This was also supported by the phylogenetic analysis of the 13 species, in which *R. esculentum* was found to exhibit fewer gene gains (331) and fewer gene losses (294) compared with *H. vulgaris* (513 gains and 666 losses) and *A. aurita* (696 gains and 962 losses) (Fig. 4). This indicated that *R. esculentum* might have evolved slower than the other scyphozoan species used in this study.

Analysis of toxin-like genes in jellyfish

Jellyfish is one important lineage of extant venomous animals [43, 44]. The venom is injected into the victim or prey when triggered to discharge. Jellyfish stings are dangerous to swimmers and fishermen because they can cause local oedema, vesicular eruption, shock, and even death [45, 46]. The venom of jellyfish consists of polypeptides, enzymes and some non-protein bioactive components [44], such as neurotoxins, myotoxins, hemolytic toxins and cardiotoxins [47]. The venom constituents of jellyfish have been investigated by pharmacological studies in recent years. Omics analyses, especially transcriptomic and proteomic analyses, have been used to conduct large-scale identification of toxins and related genes from jellyfish, and

many putative toxins have been identified [43, 46-49]. However, due to the limitation of genome information and sampling [46], the overall understanding of toxin-like genes is limited, which may be responsible for the lack of consistency among the results obtained from previous studies [49]. Here, we conducted a genomic survey of toxin-like genes in the assembled *R. esculentum* genome.

In step 1, all the genes of *R. esculentum* were screened using BLASTP with a cutoff *E-value* of 1e^{-10} against the database of animal toxin annotation project (Tox-Prot) in UniProt. In step 2, according to the best hits of gene annotations of NR, Uniprot and Tox-Prot, the genes that were consistently annotated as toxin-like genes were then chosen. In step 3, to make the pool of venom-related genes more complete, we checked all the gene annotations of the jellyfish and picked out the genes where the annotations were consistent with the annotations in the database of Tox-Prot and were not identified in the first two steps. These genes were also considered as toxin-like genes.

There were 127 toxin-like genes identified, including 60 metalloproteinases, 18 phospholipases, 13 nucleases and nucleotidases, 13 peptidases and inhibitors, 12 genes with toxin activity and 11 other venom-related genes (Table 2). It is not surprising that metalloproteases were the most abundant group of toxins because they are widely considered to be a key toxic component in various venomous animals, such as spiders [50], snakes [51], scorpions [52] and jellyfish [46, 53]. Metalloprotease can interfere with blood coagulation and induce necrosis. Metalloprotease is always associated with the symptoms of stings, such as swelling, myonecrosis, inflammation and blister formation [44, 49].

Phospholipases comprise the second most abundant group of toxins. Various forms of phospholipases have been identified, such as phospholipase A2, acidic phospholipase A2 PA4, phospholipase A2 isozymes PA3A/PA3B/PA5 and putative phospholipase B-like 2. Phospholipases are ubiquitous in the venom of many poisonous animals and they exhibit various degrees of toxicity, among which hemolytic activity is the most striking one [47]. High levels of phospholipase A2 activity have been observed in the tentacles of scyphozoan and cubozoan species [47, 54] and are presumably involved in defence and in the capturing of prey [44]. In the present study, nine copies of phospholipase were found in a tandem fashion located on three loci of the genome.

Two copies of “jellyfish toxin”, also called cubozoan-related porins, were also found. The “jellyfish toxins” have been observed in high abundance in cubozoan venoms [48] and they have also been reported in other medusozoans, such as Scyphozoan [47], Hydrozoan [55] and Anthozoan [56]. They are potent and rapid-acting toxins, having both hemolytic and pore-forming activities [44, 47]. Compared with the high abundance in cubozoans, where as many as 15 isoforms of the “jellyfish toxin” were found in *Chironex fleckeri*, the relatively fewer copies found in scyphozoan species may be linked to the less severe stings inflicted by these species of jellyfish [48].

Two new toxins were identified, and these were reticulocalbin and lysosomal acid phosphatase. These toxins have not been reported in jellyfish. Reticulocalbin is known to have calcium ion-binding activity. Its role in venom is still unclear, though it was

speculated to play a potentially unknown role in prey incapacitation by binding with phospholipase A2 [57, 58]. Lysosomal acid phosphatase is an ortholog of venom acid phosphatase, which is an acidic heat-labile protein with carbohydrate IgE binding epitopes [59]. It is mostly found in honeybee and has been implicated in allergic reaction [59-61]. The discovery of these toxin-coding genes in *R. esculenum* would add to a growing understanding of the composition of jellyfish venoms. When compared with the venom composition of the jellyfish *N. nomurai* (also named *Stomolophus meleagris*), a species closely related to *R. esculentum*, it was noted that two types of main toxins were lost in *R. esculentum*, including a serine protease inhibitor (only one copy found) and a potassium channel inhibitor ShK [46]. They are known to block the activities of trypsin and plasmin and to function as neurotoxins [46]. The different compositions of the venom may account for the different symptoms after the sting. For instance, *R. esculentum* sting always causes strong pruritus compared with stings of other jellyfish species [62].

Interestingly, eight toxin-like genes were located closely on contig 521 as a “hub”, including four PLA2s, two ENPP5s, one TRPA1 and one SLC47A1 (Table 3). The functions of toxin-like genes in the hub included phospholipase A2 activity, nuclease activity, toxin activity, and toxin extrusion. In addition, according to the chromosome-level analysis, contig 747 and contig 751 were located on the two sides of contig 521 and contained five and three toxin-like genes, respectively. These three contigs were arranged in chromosome 7 (3,691,690~13,486,489 bp) as a head-to-tail tandem, forming a bigger “hub”. The neighboring genes have been shown to co-express rather

than express independently [63, 64]. Thus, we speculated that contig747-contig521-contig751 tandem on chromosome 7 may play important roles in the formation and function of venom in *R. esculentum*. Further studies are needed to clarify their specific functions.

In summary, we have sequenced and assembled the genome of *R. esculentum* at chromosome-level. The obtained genome data would provide a valuable resource for conducting further study on *R. esculentum* and other Cnidarian species.

Availability of supporting data

The raw genome sequencing data obtained by Illumina and PacBio platform are available via NCBI with accession numbers SRR8617500 and SRR8617499 respectively (BioProject accession number PRJNA523480). The raw sequencing data of the transcriptome are available via NCBI with accession numbers SRR8401786-SRR8401797 (BioProject accession number PRJNA512552). The transcriptome assembly is available in Gigadb.

Competing interests

The authors declare that they have no competing interests.

Abbreviations

CDS, Coding Domain Sequence; NCBI, National Center for Biotechnology Information; BLAST, Basic Local Alignment Search Tool; BUSCO, Benchmarking

Universal Single-Copy Orthologs; GO, Gene Ontology; NR, Non Redundant database; KOG, Eukaryotic Orthologous Groups; KEGG, Kyoto Encyclopedia of Genes and Genomes; CAFE, computational analysis of gene family evolution.

Ethics statement

This study was approved by the Animal Care and Use Committee of Liaoning Ocean and Fisheries Science Research Institute. This study did not involve endangered or protected species.

Author contributions

Z.Z. and Y.L. designed the project. M.T. and Y.L. collected the samples. Y.P., C.H. and Y.D. extracted the genomic DNA. L.G., Y.S. and Y.P. participated in data analyses. L.G. and Y.S. wrote the manuscript. All authors have read and approved the final manuscript.

Funding

This work was supported by the National Natural Science Foundation of China (31302173; 31602156; 31602155); the Science and Technology Program of Liaoning Province, China (2013203001); the Natural Science Foundation of Liaoning Province, China (20180551158); the Scientific Research Program of Ocean and Fisheries Administration of Liaoning Province, China (201827); Liaoning Science Public Welfare Research Fund Project (20180015).

References

1. Dunn CW, Hejnol A, Matus DQ et al. Broad phylogenomic sampling improves resolution of the animal tree of life. Nature 2008;**452**(7188):745.
2. Dong Z, Liu D and Keesing JK. Contrasting trends in populations of *Rhopilema esculentum* and *Aurelia aurita* in Chinese waters. Jellyfish blooms. Springer; 2014. p. 207-18.
3. Zhuang Y, Hou H, Zhao X et al. Effects of collagen and collagen hydrolysate from jellyfish (*Rhopilema esculentum*) on mice skin photoaging induced by UV irradiation. J Food Sci 2009;**74**(6):H183-H8.
4. Dong Z, Liu D and Keesing JK. Jellyfish blooms in China: dominant species, causes and consequences. Mar Pollut Bull 2010;**60**(7):954-63.
5. Fisheries MoABo. 2018 China Fisheries Statistical Yearbook. Beijing: China Agriculture Publishing Company; 2018.
6. Gold DA, Katsuki T, Li Y et al. The genome of the jellyfish *Aurelia* and the evolution of animal complexity. Nat Ecol Evol 2019;**3**(1):96.
7. Khalturin K, Shinzato C, Khalturina M et al. Medusozoan genomes inform the evolution of the jellyfish body plan. Nat Ecol Evol 2019;**3**(5):811.
8. Kim H-M, Weber JA, Lee N et al. The genome of the giant Nomura's jellyfish sheds light on the early evolution of active predation. BMC Biology 2019;**17**(1):28.
9. Ohdera A, Ames CL, Dikow RB et al. Box, stalked, and upside-down? Draft genomes from diverse jellyfish (Cnidaria, Acraspeda) lineages: *Alatina alata* (Cubozoa), *Calvadosia cruxmelitensis* (Staurozoa), and *Cassiopea xamachana* (Scyphozoa). GigaScience 2019;**8**(7):giz069.
10. Leclère L, Horin C, Chevalier S et al. The genome of the jellyfish *Clytia hemisphaerica* and the evolution of the cnidarian life-cycle. Nat Ecol Evol 2019;**3**(5):801.
11. Marçais G and Kingsford C. A fast, lock-free approach for efficient parallel counting of occurrences of *k*-mers. Bioinformatics 2011;**27**(6):764-70.
12. Vurture GW, Sedlazeck FJ, Nattestad M et al. GenomeScope: fast reference-free genome profiling from short reads. Bioinformatics 2017;**33**(14):2202-4.
13. Zhang X, Sun L, Yuan J et al. The sea cucumber genome provides insights into morphological evolution and visceral regeneration. PLoS Biol 2017;**15**(10):e2003790.
14. Zhang G, Fang X, Guo X et al. The oyster genome reveals stress adaptation and complexity of shell formation. Nature 2012;**490**(7418):49-54.
15. Stanke M, Diekhans M, Baertsch R et al. Using native and syntenically mapped cDNA alignments to improve *de novo* gene finding. Bioinformatics 2008;**24**(5):637-44.
16. Majoros WH, Pertea M and Salzberg SL. TigrScan and GlimmerHMM: two open source *ab initio* eukaryotic gene-finders. Bioinformatics 2004;**20**(16):2878-9.
17. Korf I. Gene finding in novel genomes. BMC Bioinformatics 2004;**5**(1):59.
18. Birney E and Durbin R. Using GeneWise in the *Drosophila* annotation experiment. Genome Res 2000;**10**(4):547-8.
19. Birney E, Clamp M and Durbin R. GeneWise and genomewise. Genome Res 2004;**14**(5):988-95.
20. Trapnell C, Pachter L and Salzberg SL. TopHat: discovering splice junctions with RNA-Seq. Bioinformatics 2009;**25**(9):1105-11.
21. Trapnell C, Roberts A, Goff L et al. Differential gene and transcript expression analysis of RNA-seq experiments with TopHat and Cufflinks. Nature protocols 2012;**7**(3):562.

22. Haas BJ, Salzberg SL, Zhu W et al. Automated eukaryotic gene structure annotation using EVidenceModeler and the Program to Assemble Spliced Alignments. Genome Biol 2008;**9**(1):1.
23. Benson DA, Karsch-Mizrachi I, Lipman DJ et al. GenBank. Nucleic Acids Res 2005;**33**(suppl_1):D34-D8.
24. Consortium GO. Gene Ontology annotations and resources. Nucleic Acids Res 2012;**41**(D1):D530-D5.
25. Bairoch A and Apweiler R. The SWISS-PROT protein sequence database and its supplement TrEMBL in 2000. Nucleic Acids Res 2000;**28**(1):45-8.
26. Tatusov RL, Fedorova ND, Jackson JD et al. The COG database: an updated version includes eukaryotes. BMC Bioinformatics 2003;**4**(1):41.
27. Kanehisa M and Goto S. KEGG: kyoto encyclopedia of genes and genomes. Nucleic Acids Res 2000;**28**(1):27-30.
28. Waterhouse RM, Seppey M, Simão FA et al. BUSCO applications from quality assessments to gene prediction and phylogenomics. Mol Biol Evol 2017;**35**(3):543-8.
29. Zhu W, Hu B, Becker C et al. Altered chromatin compaction and histone methylation drive non-additive gene expression in an interspecific *Arabidopsis* hybrid. Genome Biol 2017;**18**(1):157. doi:10.1186/s13059-017-1281-4.
30. Servant N, Varoquaux N, Lajoie BR et al. HiC-Pro: an optimized and flexible pipeline for Hi-C data processing. Genome Biol 2015;**16**(259. doi:10.1186/s13059-015-0831-x.
31. Dudchenko O, Batra SS, Omer AD et al. De novo assembly of the *Aedes aegypti* genome using Hi-C yields chromosome-length scaffolds. Science 2017;**356**(6333):92-5. doi:10.1126/science.aal3327.
32. Akdemir KC and Chin L. HiCPlotter integrates genomic data with interaction matrices. Genome Biol 2015;**16**(1):198. doi:10.1186/s13059-015-0767-1.
33. Guo P. The karyotype of *Rhopilcma esculenta*. Journal of Fisheries of China 1994;**18**(3):253-5.
34. Li L, Stoeckert CJ and Roos DS. OrthoMCL: identification of ortholog groups for eukaryotic genomes. Genome Res 2003;**13**(9):2178-89.
35. Altschul SF, Madden TL, Schäffer AA et al. Gapped BLAST and PSI-BLAST: a new generation of protein database search programs. Nucleic Acids Res 1997;**25**(17):3389-402.
36. Edgar RC. MUSCLE: multiple sequence alignment with high accuracy and high throughput. Nucleic Acids Res 2004;**32**(5):1792-7.
37. Stamatakis A. RAxML version 8: a tool for phylogenetic analysis and post-analysis of large phylogenies. Bioinformatics 2014;**30**(9):1312-3.
38. Sanderson MJ. r8s: inferring absolute rates of molecular evolution and divergence times in the absence of a molecular clock. Bioinformatics 2003;**19**(2):301-2.
39. Park E, Hwang D-S, Lee J-S et al. Estimation of divergence times in cnidarian evolution based on mitochondrial protein-coding genes and the fossil record. Mol Phylogenet Evol 2012;**62**(1):329-45.
40. De Bie T, Cristianini N, Demuth JP et al. CAFE: a computational tool for the study of gene family evolution. Bioinformatics 2006;**22**(10):1269-71.
41. Grishin EV. Neurotoxin from black widow spider venom structure and function. Natural Toxins 2. Springer; 1996. p. 231-6.
42. Meldolesi J, Scheer H, Madeddu L et al. Mechanism of action of α-latrotoxin: the presynaptic stimulatory toxin of the black widow spider venom. Trends Pharmacol Sci 1986;**7**(151-5.
43. Jaimes-Becerra A, Chung R, Morandini AC et al. Comparative proteomics reveals recruitment patterns of some protein families in the venoms of Cnidaria. Toxicon 2017;**137**(19-26.

44. Jouiaei M, Yanagihara A, Madio B et al. Ancient venom systems: a review on cnidaria toxins. Toxins 2015;**7**(6):2251-71.
45. Lee H, Jung E, Kang C et al. Scyphozoan jellyfish venom metalloproteinases and their role in the cytotoxicity. Toxicon 2011;**58**(3):277-84.
46. Li R, Yu H, Xue W et al. Jellyfish venomics and venom gland transcriptomics analysis of *Stomolophus meleagris* to reveal the toxins associated with sting. J Proteomics 2014;**106**(17-29.
47. Liu G, Zhou Y, Liu D et al. Global transcriptome analysis of the tentacle of the jellyfish *Cyanea capillata* using deep sequencing and expressed sequence tags: Insight into the toxin-and degenerative disease-related transcripts. PloS one 2015;**10**(11):e0142680.
48. Brinkman DL, Jia X, Potriquet J et al. Transcriptome and venom proteome of the box jellyfish *Chironex fleckeri*. BMC Genomics 2015;**16**(1):407.
49. Li R, Yu H, Yue Y et al. Combined proteomics and transcriptomics identifies sting-related toxins of jellyfish *Cyanea nozakii*. J Proteomics 2016;**148**(57-64.
50. Trevisan-Silva D, Gremski LH, Chaim OM et al. Astacin-like metalloproteases are a gene family of toxins present in the venom of different species of the brown spider (genus *Loxosceles*). Biochimie 2010;**92**(1):21-32.
51. Markland Jr FS and Swenson S. Snake venom metalloproteinases. Toxicon 2013;**62**(3-18.
52. Brazón J, Guerrero B, D'Suze G et al. Fibrin(ogen)olytic enzymes in scorpion (*Tityus discrepans*) venom. Comp Biochem Physiol B, Biochem Mol Biol 2014;**168**(62-9.
53. Jouiaei M, Casewell NR, Yanagihara AA et al. Firing the sting: chemically induced discharge of cnidae reveals novel proteins and peptides from box jellyfish (*Chironex fleckeri*) venom. Toxins 2015;**7**(3):936-50.
54. Nevalainen TJ, Peuravuori HJ, Quinn RJ et al. Phospholipase A2 in cnidaria. Comp Biochem Physiol B, Biochem Mol Biol 2004;**139**(4):731-5.
55. Brinkman DL, Konstantakopoulos N, McInerney BV et al. *Chironex fleckeri* (box jellyfish) venom proteins: expansion of a cnidarian toxin family that elicits variable cytolytic and cardiovascular effects. J Biol Chem 2014;jbc. M113. 534149.
56. Jouiaei M, Sunagar K, Federman Gross A et al. Evolution of an ancient venom: recognition of a novel family of cnidarian toxins and the common evolutionary origin of sodium and potassium neurotoxins in sea anemone. Mol Biol Evol 2015;**32**(6):1598-610.
57. Dodds DN, Schlimgen AK, Lu SY et al. Novel reticular calcium binding protein is purified on taipoxin columns. J Neurochem 1995;**64**(5):2339-44.
58. Margres MJ, McGivern JJ, Wray KP et al. Linking the transcriptome and proteome to characterize the venom of the eastern diamondback rattlesnake (*Crotalus adamanteus*). J Proteomics 2014;**96**(145-58.
59. Hoffman D, Weimer E, Sakell R et al. Sequence and characterization of honeybee venom acid phosphatase. J Allergy Clin Immun 2005;**115**(2):S107.
60. Grunwald T, Bockisch B, Spillner E et al. Molecular cloning and expression in insect cells of honeybee venom allergen acid phosphatase (Api m 3). J Allergy Clin Immun 2006;**117**(4):848-54.
61. Kim BY and Jin BR. Molecular characterization of a venom acid phosphatase Acph-1-like protein from the Asiatic honeybee *Apis cerana*. J Asia-Pac Entomol 2014;**17**(4):695-700.
62. Kawahara M, Uye S, Burnett J et al. Stings of edible jellyfish (*Rhopilema hispidum*, *Rhopilema esculentum* and *Nemopilema nomurai*) in Japanese waters. Toxicon 2006;**48**(6):713-6.
63. Michalak P. Coexpression, coregulation, and cofunctionality of neighboring genes in eukaryotic

genomes. Genomics 2008;**91**(3):243-8.
64. Lercher MJ, Blumenthal T and Hurst LD. Coexpression of neighboring genes in *Caenorhabditis elegans* is mostly due to operons and duplicate genes. Genome Res 2003;**13**(2):238-43.

 Click here to access/download;Table;Tables-0217.docx

Table 1: Statistics of the assembly and annotation of *R. esculentum* genome.

**Genome feature**	**Parameter**
**Genome assembly**	
Total length (Mb)	275.42
Contig N50 (Mb)	1.13
Longest contig (Mb)	6.59
Contig number	760
GC content (%)	36.25
Pseudochromosome number	21
Scaffold N50 (Mb)	12.97
**Genome annotation**	
Gene number	17,219
Gene density (per 100kb)	62.52
Average CDS length (bp)	1,575
Average exon length (bp)	198.8
Average intron length (bp)	987.2
Exon number per Gene	7.92
Exon GC content (%)	42.29

Table 2: Summary of all the identified toxin-like genes from the genome of the jellyfish *R. esculentum*.

Gene	Copy number	Description	Family	Reported in jellyfish
phospholipase A2	9	phospholipase A2 activity	phospholipase A2 family	YES
Acidic phospholipase A2 PA4	4	phospholipase A2 activity	phospholipase A2 family	YES
Phospholipase A2 isozymes PA3A/PA3B/PA5	4	phospholipase A2 activity	phospholipase A2 family	YES
Putative phospholipase B-like 2	1	hydrolase activity	phospholipase B-like family	YES
Zinc metalloproteinase nas	39	metalloendopeptidase activity		YES
Disintegrin and metalloproteinase	21	metalloendopeptidase activity		YES
Ectonucleotide pyrophosphatase/phosphodiesterase	8	nuclease activity	nucleotide pyrophosphatase/ phosphodiesterase family	YES
5'-nucleotidase	5	5'-nucleotidase activity	5'-nucleotidase family	YES
serine carboxypeptidase	1	serine-type carboxypeptidase activity	peptidase S10 family	YES
serine protease	7	serine-type endopeptidase activity	peptidase S1 family	YES
Prothrombin	2	serine-type endopeptidase activity	peptidase S1 family	YES
Dipeptidyl peptidase 9	1	serine-type peptidase activity	peptidase S9B family	YES
Kunitz-type_serine_protease_inhibitor	1	serine-type endopeptidase inhibitor activity	venom Kunitz-type family	YES
Cystatin	1	cysteine-type endopeptidase inhibitor activity	cystatin family	YES
Plancitoxin-1	3	toxin activity	DNase II family	YES
Ryncolin	6	toxin activity	ficolin lectin family	YES
Toxin TX	2	toxin activity	jellyfish toxin family	YES
Trpa1	1	toxin activity	(high similarity with Alpha-latrotoxin-Lt1a)	YES
Peroxiredoxin-4	2	protein homodimerization activity	peroxiredoxin family	YES
Glutaminyl-peptide cyclotransferase-like protein	1	glutaminyl-peptide cyclotransferase activity	glutaminyl-peptide cyclotransferase family	YES
Lysosomal acid lipase/cholesteryl ester hydrolase	1	lipase activity	Lipase family	YES
Trehalase	1	alpha-trehalase activity	glycosyl hydrolase 37 family	YES
Acetylcholinesterase	1	acetylcholinesterase activity	type-B carboxylesterase/lipase family	YES
Lysosomal acid phosphatase	1	acid phosphatase activity	histidine acid phosphatase family	NO
Reticulocalbin	1	calcium ion binding	CREC family	NO
Translationally-controlled tumor protein homolog	1	calcium ion binding	TCTP family	YES
Hyaluronidase-1	2	hyaluronan synthase activity	glycosyl hydrolase 56 family	YES

Note: Full gene names are provided in the Appendix: Supplementary Table S12.

Table 3: Structure of the toxin-related hub on contig 521.

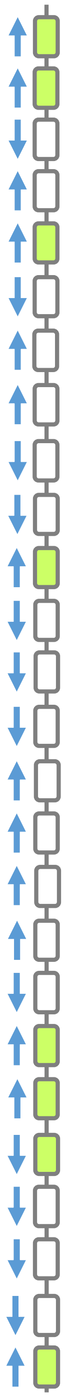

Gene	ID	Description
ENPP5	RE08134	nuclease activity
ENPP5	RE08135	nuclease activity
SLC35C2	RE08136	negative regulation of gene expression
CNTNAP5	RE08137	cell adhesion
TRPA1	RE08138	toxin activity
ADAT1	RE08139	adenosine deaminase activity
GABARAPL2	RE08140	autophagy
OSP	RE08141	zinc ion binding
EFCBP1	RE08142	calcium ion binding
DIO1	RE08143	thyroxine 5'-deiodinase activity
PLA2	RE08144	phospholipase A2 activity
KIAA1468	RE08145	---
YPT1	RE08146	GTPase activity
GCSH	RE08147	shuttling the methylamine group of glycine
SOXB2	RE08148	DNA binding
C18ORF63	RE08149	---
TBC1D20	RE08150	GTPase activator activity
AARS	RE08151	alanine-tRNA ligase activity
SAS10	RE08152	identical protein binding
PLA2	RE08153	phospholipase A2 activity
PLA2	RE08154	phospholipase A2 activity
PLA2	RE08155	phospholipase A2 activity
PDPR	RE08156	oxidoreductase activity
PDPR	RE08157	oxidoreductase activity
DHOD	RE08158	dihydroorotate dehydrogenase activity
SLC47A1	RE08159	toxin extrusion

Note: Arrows indicate the transcript direction. Green boxes represent potential toxin-like genes. Full gene names are provided in the Appendix: Supplementary Table S12.

Figure Click here to access/download;Figure;Figures-0217.docx

Figure 1: Picture of the jellyfish *R. esculentum* captured in Yingkou, Liaoning Province, China.

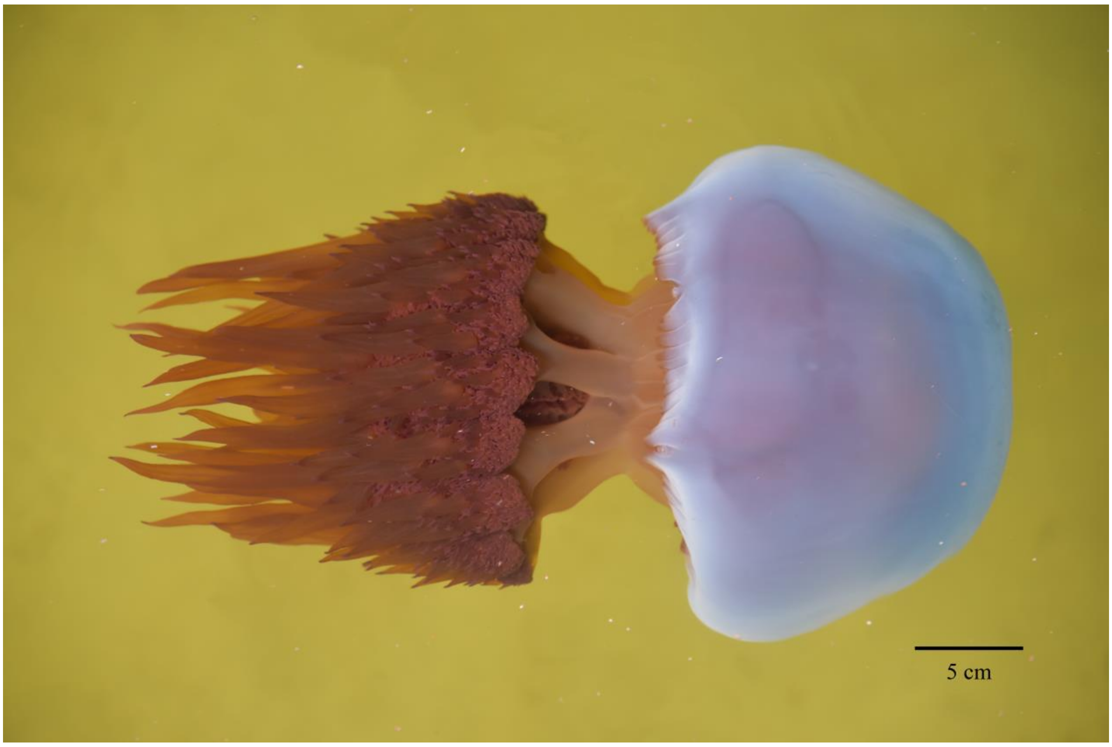

Figure 2: Schematic representation of the genomic characteristics of *R. esculentum*. Track A: 21 pseudochromosomes of *R. esculentum* genome (Mb). Track B: Protein-coding genes present in the scaffolds. Red represents genes on forward strand and green for genes on reverse strand. Track C: Distribution of gene density with sliding windows of 1 Mb. Higher density is shown in darker red color. Track D: Distribution of GC content in the genome. Track E: Distribution of repeat in the genome. Track F: Schematic presentation of major interchromosomal relationships.

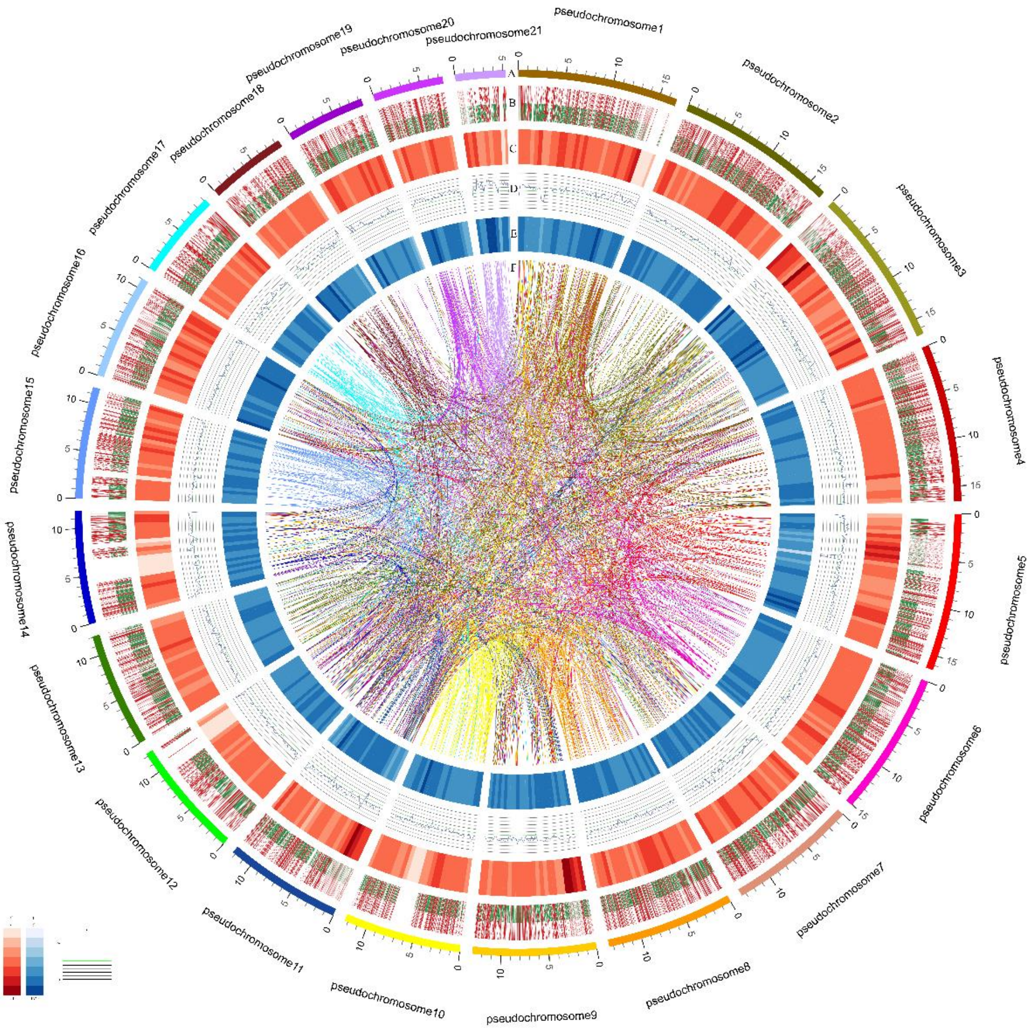

Figure 3: Venn diagram of the orthologues shared among *R. esculentum*, *H. vulgaris*, *N. vectensis* and *A. aurita*. The number of orthologous protein-coding gene clusters shared between or unique to *R. esculentum*, *H. vulgaris*, *N. vectensis* and *T. kitauei*. Each number represents the number of gene families, and the number in brackets is the number of genes.

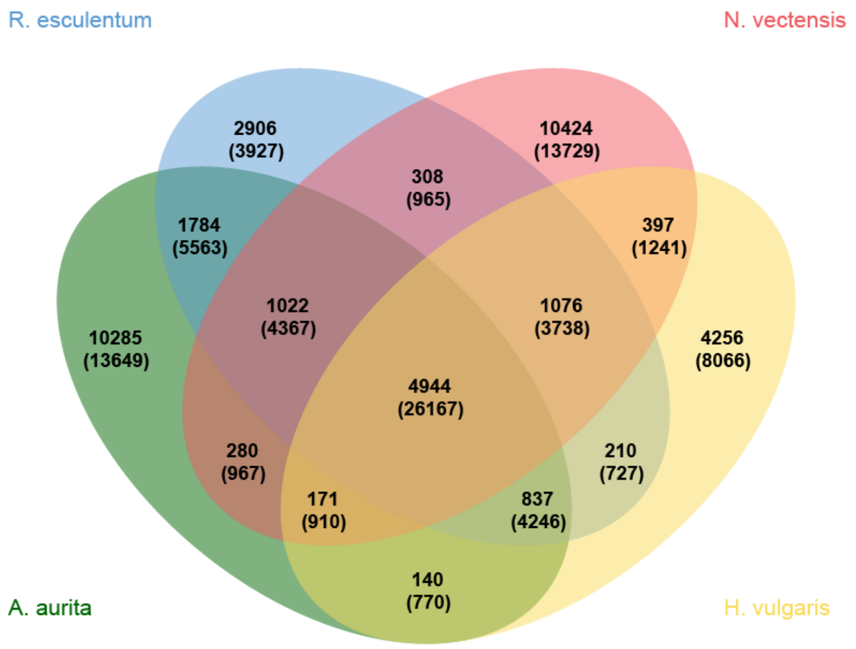

Figure 4: Phylogenetic analysis of *R. esculentum* and other metazoan species. The numbers of gene gains (+) and gene losses (−) are shown on the branches, which are also displayed as pie plots: the green part for gene gaining, the red part for gene losing and the blue part for gene remaining. The divergence times are dated and displayed below the phylogenetic tree.

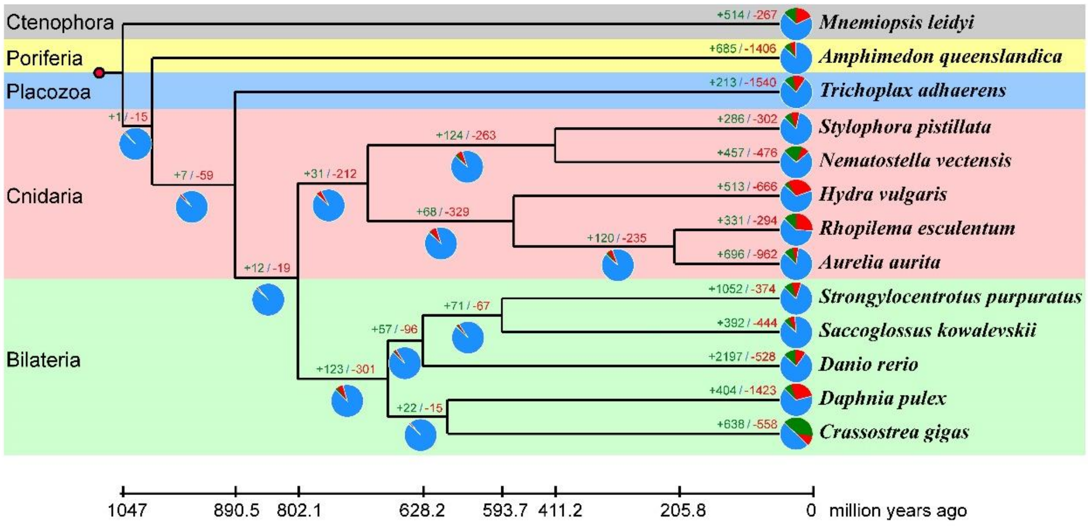

Supplementary tables and figures

Click here to access/download
Supplementary Material
Supplementary tables and figures-0217.docx
