# Supplementary material for: Chromosome-level reference genome of the jellyfish Rhopilema esculentum
Source: Gigascience. 2020 Apr 21;9(4):giaa036. doi: 10.1093/gigascience/giaa036 (PMC7172023; doi:10.1093/gigascience/giaa036)
Supplement: giaa036_GIGA-D-19-00354_Revision_2 [file giaa036_giga-d-19-00354_revision_2.pdf]

GigaScience

Chromosome-level reference genome of the jellyfish Rhopilema esculentum

--Manuscript Draft--

**Manuscript Number:**	GIGA-D-19-00354R2
**Full Title:**	Chromosome-level reference genome of the jellyfish Rhopilema esculentum
**Article Type:**	Data Note
**Funding Information:**	National Natural Science Foundation of China (31302173) — Mr. Yunfeng Li<br>National Natural Science Foundation of China (31602156) — Dr. Meilin Tian<br>National Natural Science Foundation of China (31602155) — Dr. Lei Gao<br>the Science and Technology Program of Liaoning Province, China (2013203001) — Dr. Zunchun Zhou<br>the Natural Science Foundation of Liaoning Province, China (20180551158) — Mr. Yunfeng Li<br>the Scientific Research Program of Ocean and Fisheries Administration of Liaoning Province, China (201827) — Mr. Yunfeng Li<br>Liaoning Science Public Welfare Research Fund Project (20180015) — Dr. Yulong Li

Abstract:

Background

Jellyfish belong to the phylum Cnidaria, which occupies an important phylogenetic location in the early-branching Metazoa lineages. The jellyfish Rhopilema esculentum is an important fishery resource in China. However, the genome resource of R. esculentum has not been reported to date.

Findings

In this study, we constructed a chromosome-level genome assembly of R. esculentum using Pacific Biosciences, Illumina and Hi-C sequencing technologies. The final genome assembly was approximately 275.42 Mb, with a contig N50 length of 1.13 Mb. Using Hi-C technology to identify the contacts among contigs, 260.17 Mb (94.46%) of the assembled genome were anchored onto 21 pseudochromosomes with a scaffold N50 of 12.97 Mb. We identified 17,219 protein-coding genes, with an average CDS length of 1,575 bp. The genome-wide phylogenetic analysis indicated that R. esculentum might have evolved slower than the other scyphozoan species used in this study. In addition, 127 toxin-like genes were identified, and one toxin-related “hub” was found by a genomic survey.

Conclusions

We have generated a chromosome-level genome assembly of R. esculentum that could provide a valuable genomic background for studying the biology and pharmacology of jellyfish, as well as the evolutionary history of Cnidaria.

**Corresponding Author:**	Zunchun Zhou<br><br>CHINA
**Corresponding Author Secondary Information:**	
**Corresponding Author's Institution:**	

Powered by Editorial Manager® and ProduXion Manager® from Aries Systems Corporation

**Corresponding Author's Secondary Institution:**	
**First Author:**	Yunfeng Li
**First Author Secondary Information:**	
**Order of Authors:**	Yunfeng Li
	Lei Gao
	Yongjia Pan
	Meilin Tian
	Yulong Li
	Chongbo He
	Ying Dong
	Yamin Sun
	Zunchun Zhou
**Order of Authors Secondary Information:**	
**Response to Reviewers:**	We greatly appreciate the editor and the reviewers for your time and efforts to improve our manuscript. Please see below for our point-by-point replies to the comments.<br><br>Editor:<br>1. When you mention the species sampling in the methods section, please also include the NCBI taxon ID for the species (I believe it is NCBI:txid499914, but please double check).<br>Authors' respondence: The NCBI taxon ID has been added.<br><br>2. Please include a citation to your upcoming GigaDB dataset (including the DOI link) to your reference list, and cite this in the data availability section.<br>Authors' respondence: The citation has been added and was cited in the data availability section.<br><br>Reviewer #2:<br>1. The sentence "The phylogenic results supported the view that R. esculentum, A. aurita and H. vulgaris are sister groups" is however still misleading. It could be replaced by "The phylogenetic results supported the monophyly of R. esculentum, A. aurita and H. vulgaris".<br>Authors' respondence: The manuscript has been revised as the reviewer suggested.
**Additional Information:**	
**Question**	**Response**
Are you submitting this manuscript to a special series or article collection?	No
**Experimental design and statistics**<br><br>Full details of the experimental design and statistical methods used should be given in the Methods section, as detailed in our Minimum Standards Reporting Checklist. Information essential to interpreting the data presented should be made available in the figure legends.	Yes

Powered by Editorial Manager® and ProduXion Manager® from Aries Systems Corporation

Have you included all the information requested in your manuscript?	
**Resources**<br><br>A description of all resources used, including antibodies, cell lines, animals and software tools, with enough information to allow them to be uniquely identified, should be included in the Methods section. Authors are strongly encouraged to cite Research Resource Identifiers (RRIDs) for antibodies, model organisms and tools, where possible.<br><br>Have you included the information requested as detailed in our Minimum Standards Reporting Checklist?	Yes
**Availability of data and materials**<br><br>All datasets and code on which the conclusions of the paper rely must be either included in your submission or deposited in publicly available repositories (where available and ethically appropriate), referencing such data using a unique identifier in the references and in the "Availability of Data and Materials" section of your manuscript.<br><br>Have you have met the above requirement as detailed in our Minimum Standards Reporting Checklist?	Yes

Manuscript

Click here to access/download;Manuscript;JellyfishManuscript-0322.docx

Chromosome-level reference genome of the jellyfish *Rhopilema esculentum*

Yunfeng Li[1,†], Lei Gao[1,†], Yongjia Pan[1,†], Meilin Tian[1], Yulong Li[1], Chongbo He[1], Ying Dong[1], Yamin Sun[2,*], Zunchun Zhou[1,*]

[1]Liaoning Ocean and Fisheries Science Research Institute, 50 Heishijiao St., Dalian, Liaoning 116023, China, [2]Tianjin Biochip Corporation, 23 Hongda St., Tianjin 300457, China

[*]**Correspondence address.** Zunchun Zhou, Liaoning Ocean and Fisheries Science Research Institute, 50 Heishijiao St., Dalian, Liaoning 116023, China, E-mail: zunchunz@hotmail.com; Yamin Sun, Tianjin Biochip Corporation, 23 Hongda St., Tianjin 300457, China, E-mail: nksunyamin@aliyun.com.

[†]These authors contributed equally to this work.

// Abstract

Background: Jellyfish belong to the phylum Cnidaria, which occupies an important phylogenetic location in the early-branching Metazoa lineages. The jellyfish *Rhopilema esculentum* is an important fishery resource in China. However, the genome resource of *R. esculentum* has not been reported to date. **Findings:** In this study, we constructed a chromosome-level genome assembly of *R. esculentum* using Pacific Biosciences, Illumina and Hi-C sequencing technologies. The final genome assembly was approximately 275.42 Mb, with a contig N50 length of 1.13 Mb. Using Hi-C technology to identify the contacts among contigs, 260.17 Mb (94.46%) of the assembled genome were anchored onto 21 pseudochromosomes with a scaffold N50 of 12.97 Mb. We identified 17,219 protein-coding genes, with an average CDS length of 1,575 bp. The genome-wide phylogenetic analysis indicated that *R. esculentum* might have evolved slower than the other scyphozoan species used in this study. In addition, 127 toxin-like genes were identified, and one toxin-related "hub" was found by a genomic survey. **Conclusions:** We have generated a chromosome-level genome assembly of *R. esculentum* that could provide a valuable genomic background for studying the biology and pharmacology of jellyfish, as well as the evolutionary history of Cnidaria.

Keywords: Jellyfish; *Rhopilema esculentum*; whole-genome sequencing; chromosome-level assembly; toxin-like genes

Data Description

Background

Jellyfish belong to the phylum Cnidaria, which occupies an important phylogenetic location and is one of the earliest branching Metazoa lineages [1]. The jellyfish *Rhopilema esculentum* (Kishinouye, 1891), an edible species in the class Scyphozoa (also named true jellyfish), is widely distributed in the seas around China, Japan and Korea [2], and it is one of the most abundant fishery animals in these locations. *R. esculentum* has been exploited as food for thousands of years and has been gaining more attention recently because of its pharmacological properties [3]. In contrast to many other jellyfish species that have drawn public attention because of their harmful blooms [4], the population of *R. esculentum* has declined in recent years as a result of overfishing [2]. The stock enhancement and aquaculture of *R. esculentum* have been initiated to meet the expanding market demand, which accounts for about 82,280 tons per year, generating US$ 122,800,000 worth of profit per year for the Chinese economy [5]. The lack of genomic resource has limited the phylogenetic study of jellyfish and the investigation of their many specific characteristics. Recently, several genome assemblies have been reported for the medusozoan species, including the moon jellyfish (*Aurelia aurita*) [6, 7], the giant Nomura's jellyfish (*Nemopilema nomurai*) [8], the upside-down jellyfish (*Cassiopea xamachana*) [9], the hydrozoan jellyfish *Clytia hemisphaerica* [10], *Morbakka virulenta* [7], *Alatina alata* [9] and *Calvadosia cruxmelitensis* [9]. However, no chromosome-level reference genome has been reported for the class Scyphozoa, and at present, there is very limited information of

the genome architecture of *R. esculentum*. In the present study, we sequenced the chromosome-level genome of *R. esculentum*, assembled and annotated it to improve our understanding of the evolutionary and pharmacology characteristics of jellyfish.

Sample and sequencing

One cultured *R. esculentum* (NCBI:txid499914) individual was collected from Yingkou, Liaoning Province, China (Fig. 1). After starving for two days, the epidermis tissue was sampled, and Genomic DNA was extracted using a TIANamp Marine Animal DNA Kits (Tiangen, Beijing, China) and then directly used for the genomic DNA sequencing. The genomic DNA was sheared using a sonication device, and the resulting fragments were used for the construction of short-insert paired-end (PE) library. The short-insert libraries with a size of 500 bp were constructed according to the instruction described in the Illumina library preparation kit. All libraries were sequenced on an Illumina HiSeq2500 platform (Illumina, San Diego, CA, USA) with150-bp paired-ends. In total, approximately 22.6 Gb (80×) of raw data were generated, and 20.03 Gb (71×) of clean data were filtered by FastQC (FastQC, RRID:SCR_014583) v0.11.2 (Supplementary Table S1). The genomic DNA used for sequencing was also sheared to yield ~20 kb fragments for the construction of PacBio library. DNA fragments below 7 kb were filtered using BluePipin (Sage Science, MA, USA). The filtered DNA was then converted into the proprietary SMRTbell library using the PacBio DNA Template Preparation Kit. In total, 39.76 Gb (140×) of quality-filtered data with a mean length of 7,196 bp were obtained from the PacBio Sequel platform (Supplementary Table S1).

Genome size and heterozygosity estimation

The distribution of *k*-mer frequency, also known as the *k*-mer spectrum, is widely used for the estimation of genome size. We used a jellyfish software based on a *k*-mer distribution [11] to estimate the genome size with high quality reads above Q20 from short-insert size libraries (500 bp). We obtained a *k*-mer (K=17) depth distribution from the Jellyfish analysis and clearly observed the peak depth from the distribution data. We obtained a genome size estimation of 290 Mb and a heterozygosity of 1.68% by GenomeScope v1.0.0 (Supplementary Fig. S1) [12]. 54.4% of the genome was predicted to be non-repetitive sequences.

Genome assembly and annotation

In the present study, the long reads of PacBio sequencing data was used to solve the high level of heterozygosity, which is one of the main challenges in the assembly of marine invertebrate genomes [13, 14]. The genome assembly was performed using the software wtdbg2 with default parameters (https://github.com/ruanjue/wtdbg2). The assembly sequences were then polished using Quiver (SMRT Analysis v2.3.0) with default parameters. To achieve higher continuity and accuracy for the assembled genome, five rounds of iterative error correction were performed with the Illumina clean genome data using in-house script. Finally, a genome of 275.42 Mb was assembled, with 760 contigs and a contig N50 size of 1.13 Mb (Table 1 and Supplementary Fig. S2).

Both RepeatModeler (RepeatModeler, RRID:SCR_015027) and RepeatMasker (RepeatMasker, RRID:SCR_012954) (http://www.repeatmasker.org) were used to perform the *de novo* identification and masking of repeat sequences. To ensure the integrity of the genes in subsequent analysis, all repeat sequences, except for the low complexity or simple repeats, were masked in this analysis, because some of the low complexity or simple repeats could be found in the genes. Finally, 29.23% of the assembled bases (80,495,815 bp) were masked (Supplementary Table S2). Of these, 9.93% could be annotated with known repeat families, and 19.30% were unclassified repeats.

The identification of protein-coding regions and the prediction of genes were performed using a combination of *ab initio* prediction, homology-based prediction, and transcriptome-based prediction methods. The *ab initio* gene prediction was conducted with Augustus (Augustus: Gene Prediction, RRID:SCR_008417) version 2.5.5 [15], GlimmerHMM (GlimmerHMM, RRID:SCR_002654) version 3.0.1 [16] and SNAP15 [17] to predict the coding genes. For the homology-based prediction, homologous proteins of several Cnidarian species (myxosporean (*Thelohanellus kitauei*), coral (*Stylophora pistillata* and *Orbicella faveolata*), hydrozoan (*Hydra vulgaris*), sea anemone (*Exaiptasia pallida*) and the Cnidaria EST database) were downloaded from NCBI and aligned with our assembled genome. Then, GeneWise (GeneWise, RRID:SCR_015054) version 2.2.0 [18, 19] was used to generate the gene structures based on the homology alignments. For transcriptome-based prediction, 60 individuals of four development periods (scyphistoma, strobili, ephyra and juvenile medusa) were

collected. Five individuals were pooled and three replicates were set for each development period analysis. The transcriptome of samples were sequenced using the Iillumina HiSeq2500 platform (154.6 Gb clean reads, PE-250) (Supplementary Table S3) and mapped the resulting sequences to the genome assembly using TopHat (TopHat, RRID:SCR_013035) version 2.0.8 [20]. Cufflinks (Cufflinks, RRID:SCR_014597) version 2.1.1, http://cufflinks.cbcb.umd.edu/ [21] was then used to identify the spliced transcripts in the gene models. All the gene evidence predicted from the above three approaches were integrated by EvidenceModeler (EVM) [22] into a weighted and non-redundant consensus of the gene structures. A total of 17,219 genes, with an average CDS length of 1,575 bp, were finally predicted to be present in the genome of *R. esculentum* (Table 1). All the gene sequences were searched using BLASTP with an *E-value* of $1e^{-5}$ against several public databases, including NR [23], GO (Supplementary Fig. S3) [24], Swiss [25], KOG (Supplementary Fig. S4) [26] and KEGG [27], to obtain the functional annotation. A total of 16,713 genes (97.1%) were successfully mapped to at least one database, and 8,880 genes were annotated in all four databases (*E-value* $< 1e^{-5}$) (Supplementary Fig. S5).

Quality assessment

We first aligned all the Illumina genome reads against the *R. esculentum* assembled genome using the Burrows-Wheeler Aligner (BWA, RRID:SCR_010910), version 0.7.17 to evaluate the coverage of the genome. The percentage of aligned reads was estimated to be 99.81%. BUSCO (BUSCO, RRID:SCR_015008) version: 3.0.2 [28]

was then used to evaluate the integrity of the genome (Supplementary Table S4). The values of core gene estimation were calculated as follows: C: 97.0% [S: 92.1%, D: 5.0%], F: 1.7%, M: 1.3%, n: 303, where C, S, D, F, M and n indicate complete BUSCOs, complete and single-copy BUSCOs, complete and duplicated BUSCOs, fragmented BUSCOs, missing BUSCOs and total BUSCO groups searched, respectively (Supplementary Table S5). The results indicated that the assembly covered most of the genetic regions, further confirming the assembly quality of the *R. esculentum* genome.

Pseudochromosome construction

Hi-C experiments were used for the chromosome assembly of *R. esculentum*. The whole-body homogenate of one *R. esculentum* was fixed in 1% (vol/vol) formaldehyde and was then used to prepare the Hi-C libraries. Nuclei extraction and permeabilization, chromatin digestion and proximity-ligation treatments were performed as previously described [29]. The DNA was digested overnight (12 h) with 200 U of the restriction enzyme *Mbo*I at 37 ^{o}C with shaking. The libraries were sequenced on the Illumina X-TEN platform (San Diego, CA, USA) with 2×150 bp reads. They were independently analyzed in the HiC-Pro pipeline (default parameters and LIGATION_SITE = GATC) [30]. A total of 23.96 Gb of trimmed reads were obtained, accounting for around 82-fold coverage of the *R. esculentum* genome. The 3D-DNA was used to assign the order and orientation of each group [31]. The contact maps were plotted using HiCPlotter software [32]. Finally, 260.17 Mb (94.46%) of the assembly was anchored onto 21 pseudochromosomes, which was in agreement with the Karyotype (2n=42) of *R.*

esculentum [33] (Fig. 2, Supplementary Fig. S6 and Supplementary Table S6). This chromosome-level assembly resulted in a scaffold N50 of 12.97Mb.

Phylogenetic analysis

To examine the evolutionary relationships among *R. esculentum* and other species, the whole protein sequences of *R. esculentum* and 12 other species (Supplementary Table S7) were analyzed, including species from Ctenophora (ctenophore (*Mnemiopsis leidyi*)), Porifera (demosponge (*Amphimedon queenslandica*)), Placozoa (*Trichoplax adhaerens*), Cnidaria (jellyfish (*R. esculentum* and *A. aurita*), Hydrozoa (*H. vulgaris*), coral (*S. pistillata*), sea anemone (*Nematostella vectensis*)), Protostomia (Lophotrochozoa (pacific oyster (*Crassostrea gigas*)), Ecdysozoa (cladoceran (*Daphnia pulex*))), and Deuterostomia (Echinodermata (sea urchin (*Strongylocentrotus purpuratus*)), Hemichordata (acorn worm (*Saccoglossus kowalevskii*)), Chordata (zebrafish (*Danio rerio*))). All protein models of the 12 other species were obtained from Ensembl or NCBI. Orthologous alignment analysis was performed using OrthoMCL (OrthoMCL DB: Ortholog Groups of Protein Sequences, RRID:SCR_007839) [34]. In detail, the protein-coding genes from the above-sequenced genomes were aligned with each other using the BLASTP program [35]. Similarity in the pair-wise sequence alignments generated by BLASTP was used as distance parameters for gene family clustering by MCL with an inflation value of 1.5.

A set of 32,138 gene families were eventually identified among the other 12 species, of which 2,092 families were present in all 13 species (Fig. 3 and Supplementary Table

S8). A total of 335 selected single-copy orthologous genes were aligned using MUSCLE (MUSCLE, RRID:SCR_011812) v3.6 [36] and then concatenated into a single multiple sequence alignment through an in-house Perl script. A maximum likelihood phylogeny was reconstructed using RAxML (RAxML, RRID:SCR_006086) [37] (Fig. 4). The phylogenetic results supported the monophyly of *R. esculentum*, *A. aurita* and *H. vulgaris*. PROTGAMMAJTT model was used for RaXML analyses [37]. The divergence times of *M. leidyi vs. A. aurita*, *S. purpuratus vs. A. aurita*, and *D. rerio vs. N. vectensis* were retrieved from the time tree (http://www.timetree.org) and used as the fossil calibration. R8s was used to calculate the divergence time of each node in phylogenetic tree [38]. We dated the divergence time of *R. esculentum* and *H. vulgaris* to approximately 501.71 million years ago (mya), consistent with the previous studies [39]. To compare the jellyfish genomic traits with those of the other 12 species, we performed a comparative genomic analysis for all 13 species using CAFE software (Supplementary Table S9) [40]. Twenty-seven gene families were found to be significantly expanded and another 27 gene families were found to be significantly contracted in *R. esculentum* ($P < 0.05$) (Supplementary Table S10 and Supplementary Table S11). Interestingly, the gene families enriched in the GO category of transmembrane transport were significantly expanded, and the relative GO sub-categories included drug transmembrane transport, drug transmembrane transporter activity, ion transmembrane transporter activity and amino acid transmembrane transporter activity. The action of venom, an important characteristic of jellyfish species, may contribute to gene expansion in the transmembrane transport [41, 42].

A comparative genomic analysis was performed for the four jellyfish species in the class Scyphozoa (including *R. esculentum, A. aurita, N. nomurai* and *C. xamachana*) and *H. vulgaris* (used as outgroup, and to calculate the divergence time). A total of 244 unique gene families were identified in *R. esculentum* using BLASTP with an *E-value* of 1e^{-5} in NR database. It was surprising that more than half of those (136 unique gene families) were best annotated with Anthozoa species in NR database. It was suggested that the 136 unique gene families were not from the split of *R. esculentum* but from the ancestor of Anthozoa and Scyphozoa. This result implied that some gene families that were possessed by the last common ancestor of Anthozoans and Scyphozoans were kept by the Anthozoan species and *R. esculentum*, but were lost in *A. aurita*, *N. nomurai, C. xamachana* and *H. vulgaris*. This was also supported by the phylogenetic analysis of the 13 species, in which *R. esculentum* was found to exhibit fewer gene gains (331) and fewer gene losses (294) compared with *H. vulgaris* (513 gains and 666 losses) and *A. aurita* (696 gains and 962 losses) (Fig. 4). This indicated that *R. esculentum* might have evolved slower than the other scyphozoan species used in this study.

Analysis of toxin-like genes in jellyfish

Jellyfish is one important lineage of extant venomous animals [43, 44]. The venom is injected into the victim or prey when triggered to discharge. Jellyfish stings are dangerous to swimmers and fishermen because they can cause local oedema, vesicular eruption, shock, and even death [45, 46]. The venom of jellyfish consists of polypeptides, enzymes and some non-protein bioactive components [44], such as

neurotoxins, myotoxins, hemolytic toxins and cardiotoxins [47]. The venom constituents of jellyfish have been investigated by pharmacological studies in recent years. Omics analyses, especially transcriptomic and proteomic analyses, have been used to conduct large-scale identification of toxins and related genes from jellyfish, and many putative toxins have been identified [43, 46-49]. However, due to the limitation of genome information and sampling [46], the overall understanding of toxin-like genes is limited, which may be responsible for the lack of consistency among the results obtained from previous studies [49]. Here, we conducted a genomic survey of toxin-like genes in the assembled *R. esculentum* genome.

In step 1, all the genes of *R. esculentum* were screened using BLASTP with a cutoff *E-value* of $1e^{-10}$ against the database of animal toxin annotation project (Tox-Prot) in UniProt. In step 2, according to the best hits of gene annotations of NR, Uniprot and Tox-Prot, the genes that were consistently annotated as toxin-like genes were then chosen. In step 3, to make the pool of venom-related genes more complete, we checked all the gene annotations of the jellyfish and picked out the genes where the annotations were consistent with the annotations in the database of Tox-Prot and were not identified in the first two steps. These genes were also considered as toxin-like genes.

There were 127 toxin-like genes identified, including 60 metalloproteinases, 18 phospholipases, 13 nucleases and nucleotidases, 13 peptidases and inhibitors, 12 genes with toxin activity and 11 other venom-related genes (Table 2). It is not surprising that metalloproteases were the most abundant group of toxins because they are widely considered to be a key toxic component in various venomous animals, such as spiders

[50], snakes [51], scorpions [52] and jellyfish [46, 53]. Metalloprotease can interfere with blood coagulation and induce necrosis. Metalloprotease is always associated with the symptoms of stings, such as swelling, myonecrosis, inflammation and blister formation [44, 49].

Phospholipases comprise the second most abundant group of toxins. Various forms of phospholipases have been identified, such as phospholipase A2, acidic phospholipase A2 PA4, phospholipase A2 isozymes PA3A/PA3B/PA5 and putative phospholipase B-like 2. Phospholipases are ubiquitous in the venom of many poisonous animals and they exhibit various degrees of toxicity, among which hemolytic activity is the most striking one [47]. High levels of phospholipase A2 activity have been observed in the tentacles of scyphozoan and cubozoan species [47, 54] and are presumably involved in defence and in the capturing of prey [44]. In the present study, nine copies of phospholipase were found in a tandem fashion located on three loci of the genome.

Two copies of “jellyfish toxin”, also called cubozoan-related porins, were also found. The “jellyfish toxins” have been observed in high abundance in cubozoan venoms [48] and they have also been reported in other medusozoans, such as Scyphozoan [47], Hydrozoan [55] and Anthozoan [56]. They are potent and rapid-acting toxins, having both hemolytic and pore-forming activities [44, 47]. Compared with the high abundance in cubozoans, where as many as 15 isoforms of the “jellyfish toxin” were found in *Chironex fleckeri*, the relatively fewer copies found in scyphozoan species may be linked to the less severe stings inflicted by these species of jellyfish

[48].

Two new toxins were identified, and these were reticulocalbin and lysosomal acid phosphatase. These toxins have not been reported in jellyfish. Reticulocalbin is known to have calcium ion-binding activity. Its role in venom is still unclear, though it was speculated to play a potentially unknown role in prey incapacitation by binding with phospholipase A2 [57, 58]. Lysosomal acid phosphatase is an ortholog of venom acid phosphatase, which is an acidic heat-labile protein with carbohydrate IgE binding epitopes [59]. It is mostly found in honeybee and has been implicated in allergic reaction [59-61]. The discovery of these toxin-coding genes in *R. esculenum* would add to a growing understanding of the composition of jellyfish venoms. When compared with the venom composition of the jellyfish *N. nomurai* (also named *Stomolophus meleagris*), a species closely related to *R. esculentum*, it was noted that two types of main toxins were lost in *R. esculentum*, including a serine protease inhibitor (only one copy found) and a potassium channel inhibitor ShK [46]. They are known to block the activities of trypsin and plasmin and to function as neurotoxins [46]. The different compositions of the venom may account for the different symptoms after the sting. For instance, *R. esculentum* sting always causes strong pruritus compared with stings of other jellyfish species [62].

Interestingly, eight toxin-like genes were located closely on contig 521 as a “hub”, including four PLA2s, two ENPP5s, one TRPA1 and one SLC47A1 (Table 3). The functions of toxin-like genes in the hub included phospholipase A2 activity, nuclease activity, toxin activity, and toxin extrusion. In addition, according to the chromosome-

level analysis, contig 747 and contig 751 were located on the two sides of contig 521 and contained five and three toxin-like genes, respectively. These three contigs were arranged in chromosome 7 (3,691,690~13,486,489 bp) as a head-to-tail tandem, forming a bigger “hub”. The neighboring genes have been shown to co-express rather than express independently [63, 64]. Thus, we speculated that contig747-contig521-contig751 tandem on chromosome 7 may play important roles in the formation and function of venom in *R. esculentum*. Further studies are needed to clarify their specific functions.

In summary, we have sequenced and assembled the genome of *R. esculentum* at chromosome-level. The obtained genome data would provide a valuable resource for conducting further study on *R. esculentum* and other Cnidarian species.

Availability of supporting data

The raw genome sequencing data obtained by Illumina and PacBio platform are available via NCBI with accession numbers SRR8617500 and SRR8617499 respectively (BioProject accession number PRJNA523480). The raw sequencing data of the transcriptome are available via NCBI with accession numbers SRR8401786-SRR8401797 (BioProject accession number PRJNA512552). Supporting data are available via the *GigaScience* GigaDB repository [65].

Additional files

Supplementary Fig. S1: *k*-mer estimation of the genome size of *R. esculentum.*

Supplementary Fig. S2: Contig length distribution of the assembled genome of *R. esculentum.*

Supplementary Fig. S3: GO analysis and functional classification of the protein coding genes in *R. esculentum.*

Supplementary Fig. S4: KOG analysis and functional classification of the protein coding genes in *R. esculentum*.

Supplementary Fig. S5: Venn diagram of the statistics of the functional annotation.

Supplementary Fig. S6: Interaction frequency distribution of Hi-C links among chromosomes of *R. esculentum.*

Supplementary Table S1: Statistics of the clean data of Illumina and PacBio sequencing for *R. esculentum*.

Supplementary Table S2: Statistics of the repeat elements of *R. esculentum* genome assembly indicated by both RepeatModeler and RepeatMasker software.

Supplementary Table S3: Summary of the transcriptome sequenced data of *R. esculentum*.

Supplementary Table S4: Core gene estimation for the *R. esculentum* genome assembly obtained using BUSCO.

Supplementary Table S5: BUSCO scores of gene model and trinity assembly of *R. esculentum.*

Supplementary Table S6: Quantity of the contigs anchored with Hi-C.

Supplementary Table S7: Information of the 12 representative species that used in the analysis of evolutionary relationships.

Supplementary Table S8: Summary of the orthologous gene clusters analyzed in 13 species that used in the analysis of evolutionary relationships.

Supplementary Table S9: Gene family analysis performed with CAFE.

Supplementary Table S10: Annotations of the significantly expanded gene families of *R. esculentum.*

Supplementary Table S11: Annotations of the significantly contracted gene families of *R. esculentum.*

Supplementary Table S12: Abbreviations and full names of the genes used in this study.

Competing interests

The authors declare that they have no competing interests.

Abbreviations

CDS, Coding Domain Sequence; NCBI, National Center for Biotechnology Information; BLAST, Basic Local Alignment Search Tool; BUSCO, Benchmarking Universal Single-Copy Orthologs; GO, Gene Ontology; NR, Non Redundant database; KOG, Eukaryotic Orthologous Groups; KEGG, Kyoto Encyclopedia of Genes and Genomes; CAFE, computational analysis of gene family evolution.

Ethics statement

This study was approved by the Animal Care and Use Committee of Liaoning

Ocean and Fisheries Science Research Institute. This study did not involve endangered or protected species.

Author contributions

Z.Z. and Y.L. designed the project. M.T. and Y.L. collected the samples. Y.P., C.H. and Y.D. extracted the genomic DNA. L.G., Y.S. and Y.P. participated in data analyses. L.G. and Y.S. wrote the manuscript. All authors have read and approved the final manuscript.

Funding

This work was supported by the National Natural Science Foundation of China (31302173; 31602156; 31602155); the Science and Technology Program of Liaoning Province, China (2013203001); the Natural Science Foundation of Liaoning Province, China (20180551158); the Scientific Research Program of Ocean and Fisheries Administration of Liaoning Province, China (201827); Liaoning Science Public Welfare Research Fund Project (20180015).

References

1. Dunn CW, Hejnol A, Matus DQ et al. Broad phylogenomic sampling improves resolution of the animal tree of life. Nature 2008;**452**(7188):745.
2. Dong Z, Liu D, Keesing JK. Contrasting trends in populations of *Rhopilema esculentum* and *Aurelia aurita* in Chinese waters. Jellyfish blooms. Springer; 2014. p. 207-18.
3. Zhuang Y, Hou H, Zhao X et al. Effects of collagen and collagen hydrolysate from jellyfish (*Rhopilema esculentum*) on mice skin photoaging induced by UV irradiation. J Food Sci 2009;**74**(6):H183-H8.
4. Dong Z, Liu D, Keesing JK. Jellyfish blooms in China: dominant species, causes and consequences. Mar Pollut Bull 2010;**60**(7):954-63.
5. Fisheries MoABo. 2018 China Fisheries Statistical Yearbook. Beijing: China Agriculture Publishing Company; 2018.

6. Gold DA, Katsuki T, Li Y et al. The genome of the jellyfish *Aurelia* and the evolution of animal complexity. Nat Ecol Evol 2019;**3**(1):96.
7. Khalturin K, Shinzato C, Khalturina M et al. Medusozoan genomes inform the evolution of the jellyfish body plan. Nat Ecol Evol 2019;**3**(5):811.
8. Kim H-M, Weber JA, Lee N et al. The genome of the giant Nomura's jellyfish sheds light on the early evolution of active predation. BMC Biology 2019;**17**(1):28.
9. Ohdera A, Ames CL, Dikow RB et al. Box, stalked, and upside-down? Draft genomes from diverse jellyfish (Cnidaria, Acraspeda) lineages: *Alatina alata* (Cubozoa), *Calvadosia cruxmelitensis* (Staurozoa), and *Cassiopea xamachana* (Scyphozoa). GigaScience 2019;**8**(7):giz069.
10. Leclère L, Horin C, Chevalier S et al. The genome of the jellyfish *Clytia hemisphaerica* and the evolution of the cnidarian life-cycle. Nat Ecol Evol 2019;**3**(5):801.
11. Marçais G, Kingsford C. A fast, lock-free approach for efficient parallel counting of occurrences of *k*-mers. Bioinformatics 2011;**27**(6):764-70.
12. Vurture GW, Sedlazeck FJ, Nattestad M et al. GenomeScope: fast reference-free genome profiling from short reads. Bioinformatics 2017;**33**(14):2202-4.
13. Zhang X, Sun L, Yuan J et al. The sea cucumber genome provides insights into morphological evolution and visceral regeneration. PLoS Biol 2017;**15**(10):e2003790.
14. Zhang G, Fang X, Guo X et al. The oyster genome reveals stress adaptation and complexity of shell formation. Nature 2012;**490**(7418):49-54.
15. Stanke M, Diekhans M, Baertsch R et al. Using native and syntenically mapped cDNA alignments to improve *de novo* gene finding. Bioinformatics 2008;**24**(5):637-44.
16. Majoros WH, Pertea M, Salzberg SL. TigrScan and GlimmerHMM: two open source *ab initio* eukaryotic gene-finders. Bioinformatics 2004;**20**(16):2878-9.
17. Korf I. Gene finding in novel genomes. BMC Bioinformatics 2004;**5**(1):59.
18. Birney E, Durbin R. Using GeneWise in the *Drosophila* annotation experiment. Genome Res 2000;**10**(4):547-8.
19. Birney E, Clamp M, Durbin R. GeneWise and genomewise. Genome Res 2004;**14**(5):988-95.
20. Trapnell C, Pachter L, Salzberg SL. TopHat: discovering splice junctions with RNA-Seq. Bioinformatics 2009;**25**(9):1105-11.
21. Trapnell C, Roberts A, Goff L et al. Differential gene and transcript expression analysis of RNA-seq experiments with TopHat and Cufflinks. Nature protocols 2012;**7**(3):562.
22. Haas BJ, Salzberg SL, Zhu W et al. Automated eukaryotic gene structure annotation using EVidenceModeler and the Program to Assemble Spliced Alignments. Genome Biol 2008;**9**(1):1.
23. Benson DA, Karsch-Mizrachi I, Lipman DJ et al. GenBank. Nucleic Acids Res 2005;**33**(suppl_1):D34-D8.
24. Consortium GO. Gene Ontology annotations and resources. Nucleic Acids Res 2012;**41**(D1):D530-D5.
25. Bairoch A, Apweiler R. The SWISS-PROT protein sequence database and its supplement TrEMBL in 2000. Nucleic Acids Res 2000;**28**(1):45-8.
26. Tatusov RL, Fedorova ND, Jackson JD et al. The COG database: an updated version includes eukaryotes. BMC Bioinformatics 2003;**4**(1):41.
27. Kanehisa M, Goto S. KEGG: kyoto encyclopedia of genes and genomes. Nucleic Acids Res 2000;**28**(1):27-30.
28. Waterhouse RM, Seppey M, Simão FA et al. BUSCO applications from quality assessments to gene

prediction and phylogenomics. Mol Biol Evol 2017;**35**(3):543-8.

29. Zhu W, Hu B, Becker C et al. Altered chromatin compaction and histone methylation drive non-additive gene expression in an interspecific *Arabidopsis* hybrid. Genome Biol 2017;**18**(1):157.
30. Servant N, Varoquaux N, Lajoie BR et al. HiC-Pro: an optimized and flexible pipeline for Hi-C data processing. Genome Biol 2015;**16**:259.
31. Dudchenko O, Batra SS, Omer AD et al. De novo assembly of the *Aedes aegypti* genome using Hi-C yields chromosome-length scaffolds. Science 2017;**356**(6333):92-5.
32. Akdemir KC, Chin L. HiCPlotter integrates genomic data with interaction matrices. Genome Biol 2015;**16**(1):198.
33. Guo P. The karyotype of *Rhopilcma esculenta*. Journal of Fisheries of China 1994;**18**(3):253-5.
34. Li L, Stoeckert CJ, Roos DS. OrthoMCL: identification of ortholog groups for eukaryotic genomes. Genome Res 2003;**13**(9):2178-89.
35. Altschul SF, Madden TL, Schäffer AA et al. Gapped BLAST and PSI-BLAST: a new generation of protein database search programs. Nucleic Acids Res 1997;**25**(17):3389-402.
36. Edgar RC. MUSCLE: multiple sequence alignment with high accuracy and high throughput. Nucleic Acids Res 2004;**32**(5):1792-7.
37. Stamatakis A. RAxML version 8: a tool for phylogenetic analysis and post-analysis of large phylogenies. Bioinformatics 2014;**30**(9):1312-3.
38. Sanderson MJ. r8s: inferring absolute rates of molecular evolution and divergence times in the absence of a molecular clock. Bioinformatics 2003;**19**(2):301-2.
39. Park E, Hwang D-S, Lee J-S et al. Estimation of divergence times in cnidarian evolution based on mitochondrial protein-coding genes and the fossil record. Mol Phylogenet Evol 2012;**62**(1):329-45.
40. De Bie T, Cristianini N, Demuth JP et al. CAFE: a computational tool for the study of gene family evolution. Bioinformatics 2006;**22**(10):1269-71.
41. Grishin EV. Neurotoxin from black widow spider venom structure and function. Natural Toxins 2. Springer; 1996. p. 231-6.
42. Meldolesi J, Scheer H, Madeddu L et al. Mechanism of action of α-latrotoxin: the presynaptic stimulatory toxin of the black widow spider venom. Trends Pharmacol Sci 1986;**7**:151-5.
43. Jaimes-Becerra A, Chung R, Morandini AC et al. Comparative proteomics reveals recruitment patterns of some protein families in the venoms of Cnidaria. Toxicon 2017;**137**:19-26.
44. Jouiaei M, Yanagihara A, Madio B et al. Ancient venom systems: a review on cnidaria toxins. Toxins 2015;**7**(6):2251-71.
45. Lee H, Jung E, Kang C et al. Scyphozoan jellyfish venom metalloproteinases and their role in the cytotoxicity. Toxicon 2011;**58**(3):277-84.
46. Li R, Yu H, Xue W et al. Jellyfish venomics and venom gland transcriptomics analysis of *Stomolophus meleagris* to reveal the toxins associated with sting. J Proteomics 2014;**106**:17-29.
47. Liu G, Zhou Y, Liu D et al. Global transcriptome analysis of the tentacle of the jellyfish *Cyanea capillata* using deep sequencing and expressed sequence tags: Insight into the toxin-and degenerative disease-related transcripts. PloS one 2015;**10**(11):e0142680.
48. Brinkman DL, Jia X, Potriquet J et al. Transcriptome and venom proteome of the box jellyfish *Chironex fleckeri*. BMC Genomics 2015;**16**(1):407.
49. Li R, Yu H, Yue Y et al. Combined proteomics and transcriptomics identifies sting-related toxins of jellyfish *Cyanea nozakii*. J Proteomics 2016;**148**:57-64.
50. Trevisan-Silva D, Gremski LH, Chaim OM et al. Astacin-like metalloproteases are a gene family

of toxins present in the venom of different species of the brown spider (genus *Loxosceles*). Biochimie 2010;**92**(1):21-32.

51. Markland Jr FS, Swenson S. Snake venom metalloproteinases. Toxicon 2013;**62**:3-18.
52. Brazón J, Guerrero B, D'Suze G et al. Fibrin(ogen)olytic enzymes in scorpion (*Tityus discrepans*) venom. Comp Biochem Physiol B, Biochem Mol Biol 2014;**168**:62-9.
53. Jouiaei M, Casewell NR, Yanagihara AA et al. Firing the sting: chemically induced discharge of cnidae reveals novel proteins and peptides from box jellyfish (*Chironex fleckeri*) venom. Toxins 2015;**7**(3):936-50.
54. Nevalainen TJ, Peuravuori HJ, Quinn RJ et al. Phospholipase A2 in cnidaria. Comp Biochem Physiol B, Biochem Mol Biol 2004;**139**(4):731-5.
55. Brinkman DL, Konstantakopoulos N, McInerney BV et al. *Chironex fleckeri* (box jellyfish) venom proteins: expansion of a cnidarian toxin family that elicits variable cytolytic and cardiovascular effects. J Biol Chem 2014:jbc. M113. 534149.
56. Jouiaei M, Sunagar K, Federman Gross A et al. Evolution of an ancient venom: recognition of a novel family of cnidarian toxins and the common evolutionary origin of sodium and potassium neurotoxins in sea anemone. Mol Biol Evol 2015;**32**(6):1598-610.
57. Dodds DN, Schlimgen AK, Lu SY et al. Novel reticular calcium binding protein is purified on taipoxin columns. J Neurochem 1995;**64**(5):2339-44.
58. Margres MJ, McGivern JJ, Wray KP et al. Linking the transcriptome and proteome to characterize the venom of the eastern diamondback rattlesnake (*Crotalus adamanteus*). J Proteomics 2014;**96**:145-58.
59. Hoffman D, Weimer E, Sakell R et al. Sequence and characterization of honeybee venom acid phosphatase. J Allergy Clin Immun 2005;**115**(2):S107.
60. Grunwald T, Bockisch B, Spillner E et al. Molecular cloning and expression in insect cells of honeybee venom allergen acid phosphatase (Api m 3). J Allergy Clin Immun 2006;**117**(4):848-54.
61. Kim BY, Jin BR. Molecular characterization of a venom acid phosphatase Acph-1-like protein from the Asiatic honeybee *Apis cerana*. J Asia-Pac Entomol 2014;**17**(4):695-700.
62. Kawahara M, Uye S, Burnett J et al. Stings of edible jellyfish (*Rhopilema hispidum*, *Rhopilema esculentum* and *Nemopilema nomurai*) in Japanese waters. Toxicon 2006;**48**(6):713-6.
63. Michalak P. Coexpression, coregulation, and cofunctionality of neighboring genes in eukaryotic genomes. Genomics 2008;**91**(3):243-8.
64. Lercher MJ, Blumenthal T, Hurst LD. Coexpression of neighboring genes in *Caenorhabditis elegans* is mostly due to operons and duplicate genes. Genome Res 2003;**13**(2):238-43.
65. Li Y, Gao L, Pan Y et al. Supporting data for "Chromosome-level reference genome of the jellyfish *Rhopilema esculentum*." GigaScience Database 2020. http://dx.doi.org/10.5524/100720.

 Click here to access/download;Table;Tables-0217.docx

Table 1: Statistics of the assembly and annotation of *R. esculentum* genome.

**Genome feature**	**Parameter**
**Genome assembly**	
Total length (Mb)	275.42
Contig N50 (Mb)	1.13
Longest contig (Mb)	6.59
Contig number	760
GC content (%)	36.25
Pseudochromosome number	21
Scaffold N50 (Mb)	12.97
**Genome annotation**	
Gene number	17,219
Gene density (per 100kb)	62.52
Average CDS length (bp)	1,575
Average exon length (bp)	198.8
Average intron length (bp)	987.2
Exon number per Gene	7.92
Exon GC content (%)	42.29

Table 2: Summary of all the identified toxin-like genes from the genome of the jellyfish *R. esculentum*.

Gene	Copy number	Description	Family	Reported in jellyfish
phospholipase A2	9	phospholipase A2 activity	phospholipase A2 family	YES
Acidic phospholipase A2 PA4	4	phospholipase A2 activity	phospholipase A2 family	YES
Phospholipase A2 isozymes PA3A/PA3B/PA5	4	phospholipase A2 activity	phospholipase A2 family	YES
Putative phospholipase B-like 2	1	hydrolase activity	phospholipase B-like family	YES
Zinc metalloproteinase nas	39	metalloendopeptidase activity		YES
Disintegrin and metalloproteinase	21	metalloendopeptidase activity		YES
Ectonucleotide pyrophosphatase/phosphodiesterase	8	nuclease activity	nucleotide pyrophosphatase/ phosphodiesterase family	YES
5'-nucleotidase	5	5'-nucleotidase activity	5'-nucleotidase family	YES
serine carboxypeptidase	1	serine-type carboxypeptidase activity	peptidase S10 family	YES
serine protease	7	serine-type endopeptidase activity	peptidase S1 family	YES
Prothrombin	2	serine-type endopeptidase activity	peptidase S1 family	YES
Dipeptidyl peptidase 9	1	serine-type peptidase activity	peptidase S9B family	YES
Kunitz-type_serine_protease_inhibitor	1	serine-type endopeptidase inhibitor activity	venom Kunitz-type family	YES
Cystatin	1	cysteine-type endopeptidase inhibitor activity	cystatin family	YES
Plancitoxin-1	3	toxin activity	DNase II family	YES
Ryncolin	6	toxin activity	ficolin lectin family	YES
Toxin TX	2	toxin activity	jellyfish toxin family	YES
Trpa1	1	toxin activity	(high similarity with Alpha-latrotoxin-Lt1a)	YES
Peroxiredoxin-4	2	protein homodimerization activity	peroxiredoxin family	YES
Glutaminyl-peptide cyclotransferase-like protein	1	glutaminyl-peptide cyclotransferase activity	glutaminyl-peptide cyclotransferase family	YES
Lysosomal acid lipase/cholesteryl ester hydrolase	1	lipase activity	Lipase family	YES
Trehalase	1	alpha-trehalase activity	glycosyl hydrolase 37 family	YES
Acetylcholinesterase	1	acetylcholinesterase activity	type-B carboxylesterase/lipase family	YES
Lysosomal acid phosphatase	1	acid phosphatase activity	histidine acid phosphatase family	NO
Reticulocalbin	1	calcium ion binding	CREC family	NO
Translationally-controlled tumor protein homolog	1	calcium ion binding	TCTP family	YES
Hyaluronidase-1	2	hyaluronan synthase activity	glycosyl hydrolase 56 family	YES

Note: Full gene names are provided in the Appendix: Supplementary Table S12.

Table 3: Structure of the toxin-related hub on contig 521.

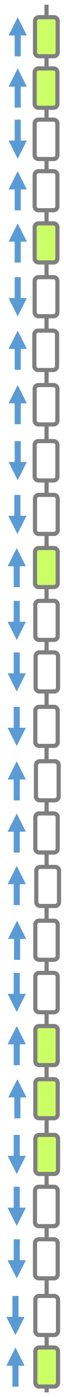

Gene	ID	Description
ENPP5	RE08134	nuclease activity
ENPP5	RE08135	nuclease activity
SLC35C2	RE08136	negative regulation of gene expression
CNTNAP5	RE08137	cell adhesion
TRPA1	RE08138	toxin activity
ADAT1	RE08139	adenosine deaminase activity
GABARAPL2	RE08140	autophagy
OSP	RE08141	zinc ion binding
EFCBP1	RE08142	calcium ion binding
DIO1	RE08143	thyroxine 5'-deiodinase activity
PLA2	RE08144	phospholipase A2 activity
KIAA1468	RE08145	---
YPT1	RE08146	GTPase activity
GCSH	RE08147	shuttling the methylamine group of glycine
SOXB2	RE08148	DNA binding
C18ORF63	RE08149	---
TBC1D20	RE08150	GTPase activator activity
AARS	RE08151	alanine-tRNA ligase activity
SAS10	RE08152	identical protein binding
PLA2	RE08153	phospholipase A2 activity
PLA2	RE08154	phospholipase A2 activity
PLA2	RE08155	phospholipase A2 activity
PDPR	RE08156	oxidoreductase activity
PDPR	RE08157	oxidoreductase activity
DHOD	RE08158	dihydroorotate dehydrogenase activity
SLC47A1	RE08159	toxin extrusion

Note: Arrows indicate the transcript direction. Green boxes represent potential toxin-like genes. Full gene names are provided in the Appendix: Supplementary Table S12.

Figure 1: Picture of the jellyfish *R. esculentum* captured in Yingkou, Liaoning Province, China.

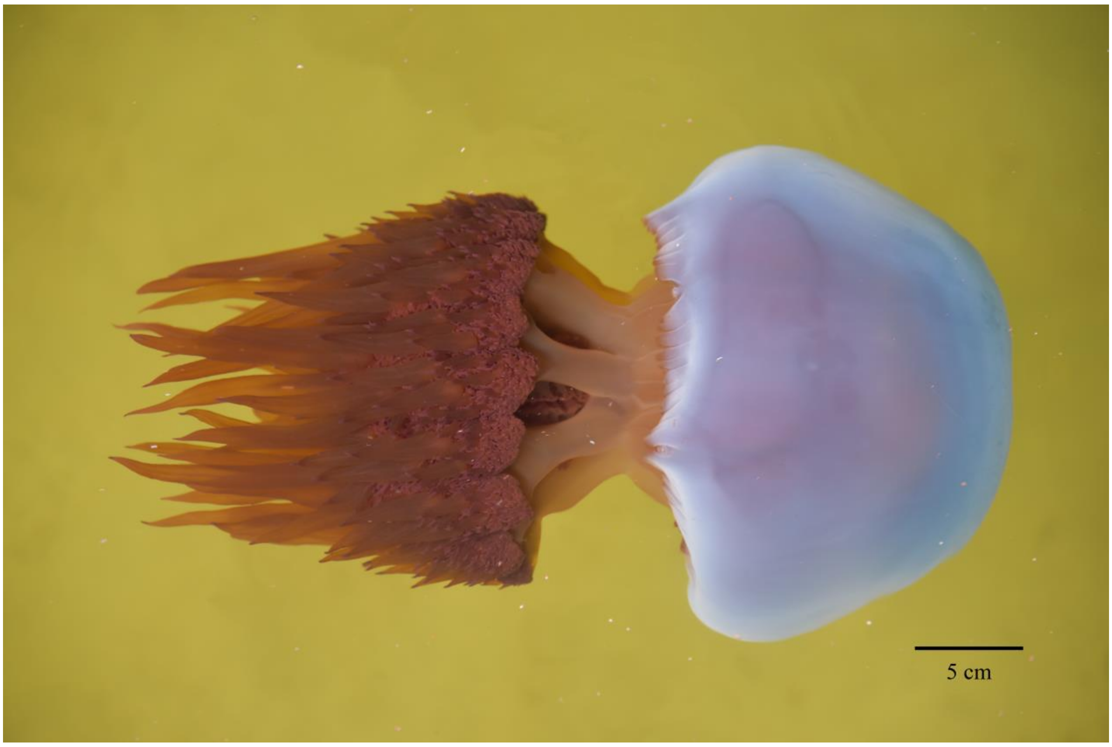

Figure 2: Schematic representation of the genomic characteristics of *R. esculentum*. Track A: 21 pseudochromosomes of *R. esculentum* genome (Mb). Track B: Protein-coding genes present in the scaffolds. Red represents genes on forward strand and green for genes on reverse strand. Track C: Distribution of gene density with sliding windows of 1 Mb. Higher density is shown in darker red color. Track D: Distribution of GC content in the genome. Track E: Distribution of repeat in the genome. Track F: Schematic presentation of major interchromosomal relationships.

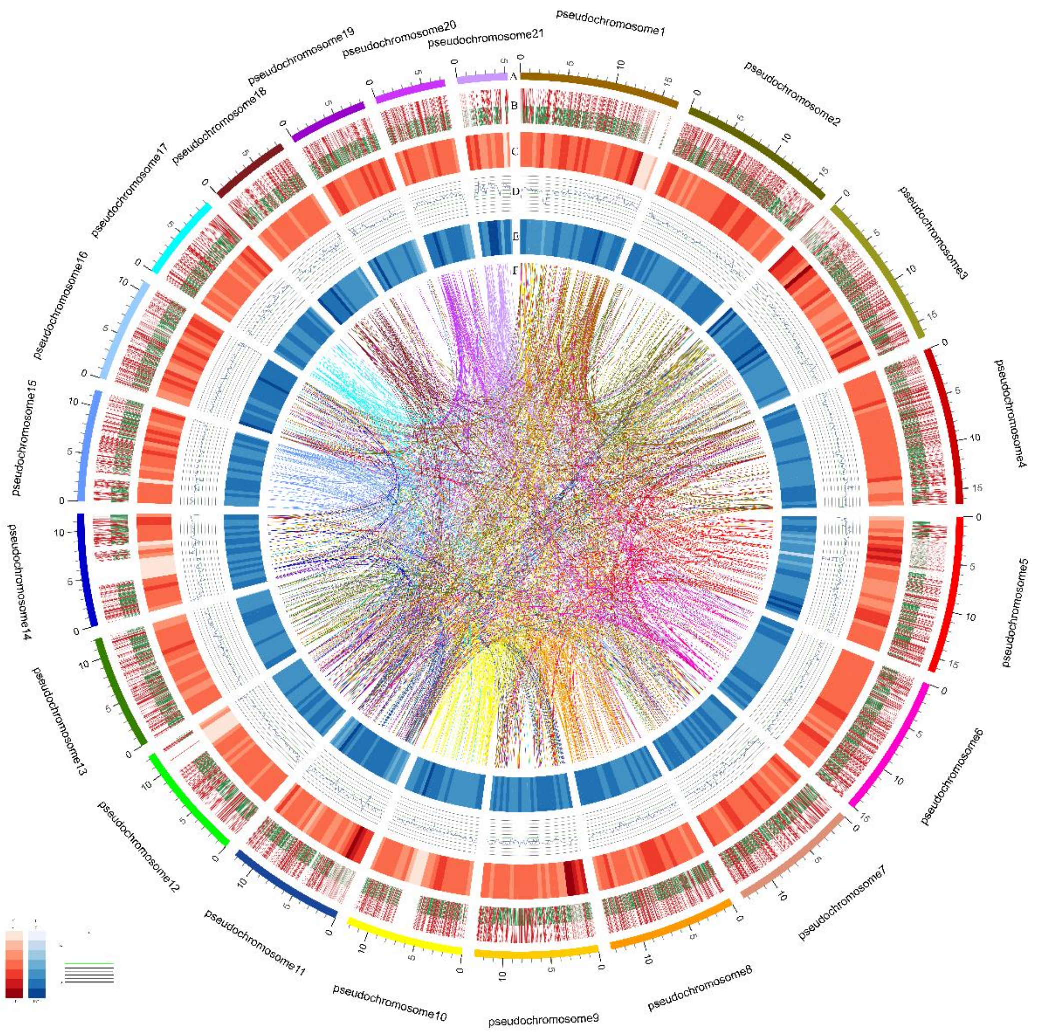

Figure 3: Venn diagram of the orthologues shared among *R. esculentum*, *H. vulgaris*, *N. vectensis* and *A. aurita*. The number of orthologous protein-coding gene clusters shared between or unique to *R. esculentum*, *H. vulgaris*, *N. vectensis* and *T. kitauei*. Each number represents the number of gene families, and the number in brackets is the number of genes.

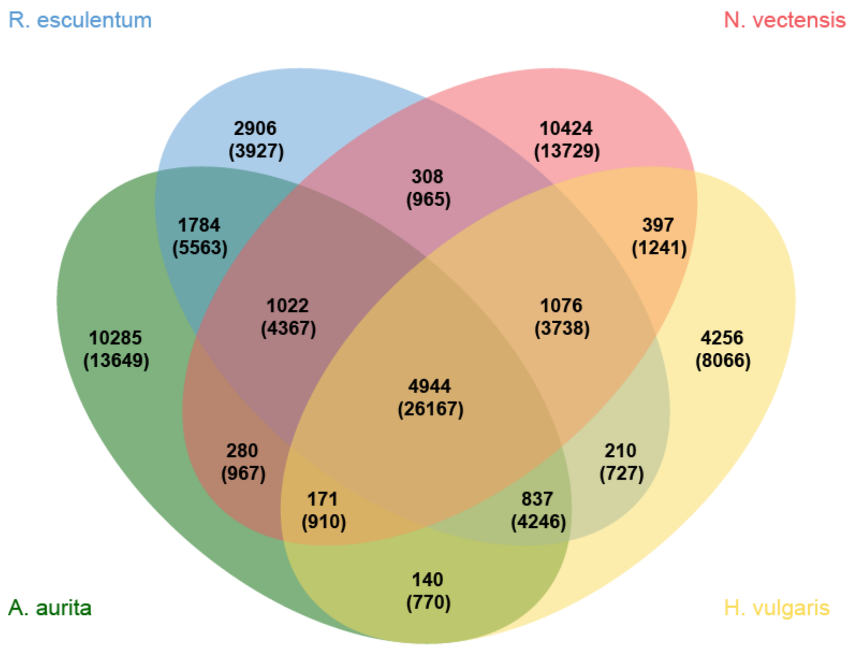

Figure 4: Phylogenetic analysis of *R. esculentum* and other metazoan species. The numbers of gene gains (+) and gene losses (−) are shown on the branches, which are also displayed as pie plots: the green part for gene gaining, the red part for gene losing and the blue part for gene remaining. The divergence times are dated and displayed below the phylogenetic tree.

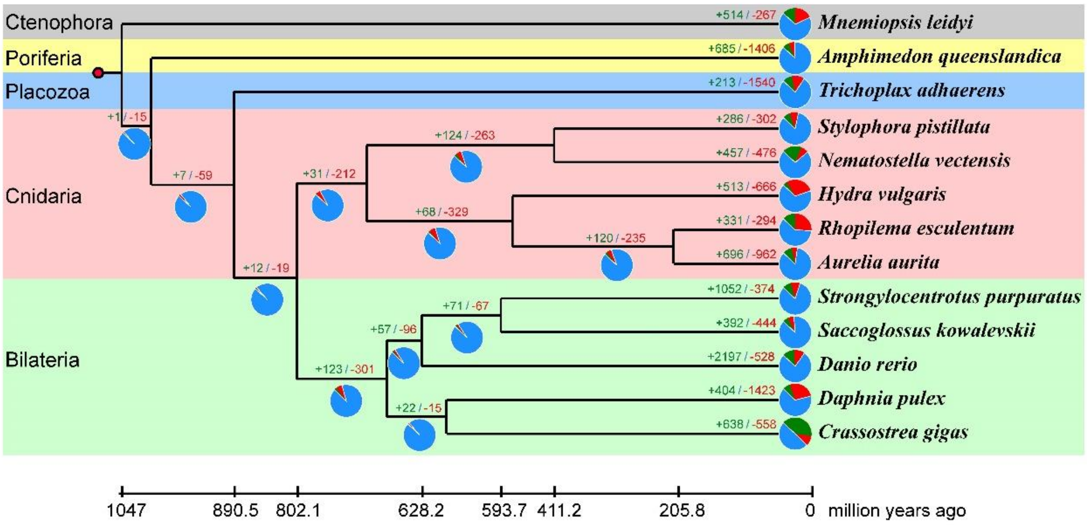

Click here to access/download
Supplementary Material
Supplementary tables and figures-0217.docx
