# Supplementary material for: Chromosome-level reference genome of the jellyfish Rhopilema esculentum
Source: Gigascience. 2020 Apr 21;9(4):giaa036. doi: 10.1093/gigascience/giaa036 (PMC7172023; doi:10.1093/gigascience/giaa036)

**Table S1. Statistics of the clean data of Illumina and PacBio sequencing for *R. esculentum*.**

| **Clean reads** | **Illumina sequencing** | **PacBio sequencing** |
| --- | --- | --- |
| **Size of library** | 500 bp | 20 kb |
| **Number of reads** | 72,870,003 *2 | 5,932,565 |
| **Average length of reads** | 147 bp | 7,196 bp |
| **Total bases** | 21,508,020,494 bp | 42,694,853,998 bp |
| **Sequencing depth** | ~71× | ~140× |

**Table S2. Statistics of the repeat elements of *R. esculentum* genome assembly indicated by both RepeatModeler and RepeatMasker software.**

|  | **Number of elements** | **Length occupied** | **Percentage of sequence** |
| --- | --- | --- | --- |
| **SINEs:** | 302 | 34,467 bp | 0.01% |
| **MIRs** | 59 | 8,294 bp | 0.00% |
|  |  |  |  |
| **LINEs:** | 27,334 | 9,288,367 bp | 3.37% |
| **LINE1** | 87 | 7,305 bp | 0.00% |
| **LINE2** | 13,032 | 4,189,046 bp | 1.52% |
| **L3/CR1** | 503 | 132,667 bp | 0.05% |
|  |  |  |  |
| **LTR elements:** | 7,293 | 4,116,160 bp | 1.49% |
| **ERV_classI** | 190 | 50,584 bp | 0.02% |
|  |  |  |  |
| **DNA elements:** | 57,611 | 13,591,053 bp | 4.93% |
| **hAT-Charlie** | 790 | 160,172 bp | 0.06% |
|  |  |  |  |
| **Unclassified:** | 259,308 | 53,153,624 bp | 19.30% |
|  |  |  |  |
| **Total interspersed repeats:** |  | 80,183,671 bp | 29.11% |
|  |  |  |  |
| **Satellites:** | 461 | 156,956 bp | 0.06% |
| **Simple repeats:** | 1,051 | 288,104 bp | 0.10% |

**Table S3. Summary of the transcriptome sequenced data of *R. esculentum***

| SRR ID | Sample | No. of raw reads | No. of clean reads | Clean bases (Gb) | Error rate (%) | Q20 (%) | Q30 (%) | GC content (%) |
| --- | --- | --- | --- | --- | --- | --- | --- | --- |
| SRR8401791 | Scyphistoma-A | 103,297,516 | 96,894,464 | 14.5 | 0.01 | 98.26 | 95.34 | 39.46 |
| SRR8401788 | Scyphistoma-B | 102,207,952 | 96,675,784 | 14.5 | 0.01 | 98.31 | 95.47 | 39.34 |
| SRR8401789 | Scyphistoma-C | 87,199,870 | 82,895,294 | 12.3 | 0.01 | 98.33 | 95.52 | 39.16 |
| SRR8401792 | Strobili-A | 96,275,930 | 81,941,336 | 13.6 | 0.01 | 97.8 | 94.41 | 40.41 |
| SRR8401793 | Strobili-B | 105,042,006 | 88,541,750 | 12.6 | 0.01 | 97.82 | 94.44 | 40.45 |
| SRR8401790 | Strobili-C | 99,248,360 | 80,988,074 | 12.4 | 0.01 | 97.81 | 94.46 | 39.86 |
| SRR8401787 | Ephyra-A | 101,753,352 | 88,464,562 | 13.6 | 0.01 | 97.09 | 93.03 | 39.69 |
| SRR8401794 | Ephyra-B | 118,485,344 | 86,673,232 | 13.1 | 0.01 | 98.04 | 94.96 | 40.81 |
| SRR8401795 | Ephyra-C | 110,317,460 | 90,876,188 | 13.9 | 0.02 | 97.05 | 92.9 | 39.83 |
| SRR8401796 | Juvenile-A | 94,582,492 | 92,374,340 | 11.2 | 0.01 | 98.28 | 95.47 | 40.7 |
| SRR8401797 | Juvenile-B | 82,452,998 | 79,242,434 | 13 | 0.01 | 98.03 | 94.95 | 40.76 |
| SRR8401786 | Juvenile-C | 96,516,182 | 93,389,182 | 13.4 | 0.01 | 98.2 | 95.29 | 40.9 |

**Table S4. Core gene estimation for the *R. esculentum* genome assembly obtained using BUSCO.**

|  | **Number** | **Percentage (%)** |
| --- | --- | --- |
| **Complete BUSCOs** | 294 | 97.03 |
| **Complete and single-copy BUSCOs** | 279 | 92.08 |
| **Complete and duplicated BUSCOs** | 15 | 4.95 |
| **Fragmented BUSCOs** | 5 | 1.65 |
| **Missing BUSCOs** | 4 | 1.32 |
| **Total BUSCO groups searched** | 303 | 100 |

**Table S5. BUSCO scores of gene model and trinity assembly of *R. esculentum***

|  | **Gene model** | | **Trinity fasta** | |
| --- | --- | --- | --- | --- |
|  | **Number** | **Percentage (%)** | **Number** | **Percentage (%)** |
| **Complete BUSCOs** | 294 | 97.1 | 277 | 91.4 |
| **Complete and single-copy BUSCOs** | 279 | 92.1 | 88 | 29 |
| **Complete and duplicated BUSCOs** | 15 | 5 | 189 | 62.4 |
| **Fragmented BUSCOs** | 5 | 1.7 | 26 | 8.6 |
| **Missing BUSCOs** | 4 | 1.2 | 0 | 0 |
| **Total BUSCO groups searched** | 303 | 100 | 303 | 100 |

**Table S6. Quantity of the contigs anchored with Hi-C.**

| **Pseudochromosome** | **Number of anchored contigs** | **Sequence Length(bp)** | **Number of genes** |
| --- | --- | --- | --- |
| pseudochromosome1 | 37 | 16,781,749 | 1,112 |
| pseudochromosome2 | 40 | 16,756,308 | 1,086 |
| pseudochromosome3 | 40 | 16,633,062 | 1,123 |
| pseudochromosome4 | 31 | 16,592,029 | 1,009 |
| pseudochromosome5 | 52 | 16,467,034 | 990 |
| pseudochromosome6 | 11 | 15,054,541 | 920 |
| pseudochromosome7 | 15 | 13,472,489 | 871 |
| pseudochromosome8 | 18 | 13,427,041 | 837 |
| pseudochromosome9 | 28 | 12,938,783 | 890 |
| pseudochromosome10 | 30 | 12,345,913 | 746 |
| pseudochromosome11 | 42 | 12,260,558 | 773 |
| pseudochromosome12 | 19 | 12,268,905 | 731 |
| pseudochromosome13 | 15 | 12,092,242 | 692 |
| pseudochromosome14 | 29 | 11,859,686 | 713 |
| pseudochromosome15 | 26 | 11,661,835 | 730 |
| pseudochromosome16 | 31 | 11,024,662 | 676 |
| pseudochromosome17 | 8 | 9,170,809 | 535 |
| pseudochromosome18 | 25 | 8,407,877 | 477 |
| pseudochromosome19 | 16 | 8,267,005 | 542 |
| pseudochromosome20 | 14 | 7,415,839 | 441 |
| pseudochromosome21 | 22 | 5,273,498 | 302 |
| **Total (Ratio)** | **549 (71.76%)** | **260,171,865 (94.46%)** | **16,196 (94.07%)** |

**Table S7. Information of the 12 representative species that used in the analysis of evolutionary relationships**

| **Species** | **Data source** |
| --- | --- |
| *Amphimedon queenslandica* | https://www.ncbi.nlm.nih.gov/genome/2698?genome_assembly_id=34059 |
| *Aurelia aurita* | www.DavidAdlerGold.com/jellyfish |
| *Crassostrea gigas* | <https://www.ncbi.nlm.nih.gov/genome/10758?genome_assembly_id=39669> |
| *Danio rerio* | <https://www.ncbi.nlm.nih.gov/genome/50?genome_assembly_id=322293> |
| *Daphnia pulex* | <https://www.ncbi.nlm.nih.gov/genome/288?genome_assembly_id=700281> |
| *Hydra vulgaris* | <https://www.ncbi.nlm.nih.gov/genome/12836?genome_assembly_id=28720> |
| *Mnemiopsis leidyi* | https://research.nhgri.nih.gov/mnemiopsis/download/download.cgi?dl=proteome |
| *Nematostella vectensis* | <https://www.ncbi.nlm.nih.gov/genome/230?genome_assembly_id=28590> |
| *Saccoglossus kowalevskii* | <https://www.ncbi.nlm.nih.gov/genome/359?genome_assembly_id=53063> |
| *Strongylocentrotus purpuratus* | <https://www.ncbi.nlm.nih.gov/genome/86?genome_assembly_id=695522> |
| *Stylophora pistillata* | <https://www.ncbi.nlm.nih.gov/genome/12040?genome_assembly_id=343043> |
| *Trichoplax adhaerens* | <https://www.ncbi.nlm.nih.gov/genome/354?genome_assembly_id=28718> |

**Table S8. Summary of the orthologous gene clusters analyzed in 13 species that used in the analysis of evolutionary relationships.**

| **Species name** | **Reference** | **No. of**  **coding genes** | **No. of gene families** | **No. of genes in gene families** | **Average No. of genes in gene families** |
| --- | --- | --- | --- | --- | --- |
| *R. esculentum* | This study | 17,219 | 10,896 | 17,219 | 1.58 |
| *A. queenslandica* | \| GCA_000090795.1 \| \| --- \| | 20,635 | 7,869 | 20,635 | 2.62 |
| *T. adhaerens* | GCA_000150275.1 | 11,517 | 6,852 | 11,517 | 1.68 |
| *H. vulgaris* | GCA_000004095.1 | 19,997 | 9,150 | 19,997 | 2.19 |
| *A. aurita* | GCA_004194395.1 | 27,044 | 11,303 | 27,044 | 2.39 |
| *S. pistillata* | GCA_002571385.1 | 24,682 | 11,922 | 24,682 | 2.07 |
| *N. vectensis* | GCA_000209225.1 | 24,772 | 11,999 | 24,772 | 2.06 |
| *C. gigas* | \| [GCA_000297895.1](https://www.ncbi.nlm.nih.gov/assembly/426118) \| \| --- \| | 28,107 | 10,968 | 28,107 | 2.56 |
| *D. pulex* | \| [GCA_000187875.1](https://www.ncbi.nlm.nih.gov/assembly/244278) \| \| --- \| | 30,595 | 8,688 | 30,595 | 3.52 |
| *S. purpuratus* | \| [GCA_000002235.3](https://www.ncbi.nlm.nih.gov/assembly/308821) \| \| --- \| | 27,593 | 10,599 | 27,593 | 2.6 |
| *S. kowalevskii* | \| [GCA_000003605.1](https://www.ncbi.nlm.nih.gov/assembly/95611) \| \| --- \| | 20,929 | 10,422 | 20,929 | 2.01 |
| *D. rerio* | \| [GCA_000002035.4](https://www.ncbi.nlm.nih.gov/assembly/1104621) \| \| --- \| | 32,385 | 10,291 | 32,385 | 3.15 |
| *M. leidyi* | GCA_000226015.1 | 16,548 | 6,214 | 16,548 | 2.66 |

**Table S9. Gene family analysis performed with CAFE**

| **Species** | **Expanded families** | **No change families** | **Contracted families** |
| --- | --- | --- | --- |
| *R. esculentum* | 331 | 4,918 | 294 |
| *A. queenslandica* | 685 | 3,452 | 1,406 |
| *T. adhaerens* | 213 | 3,790 | 1,540 |
| *H. vulgaris* | 513 | 4,364 | 666 |
| *M. leidyi* | 514 | 4,762 | 267 |
| *S. pistillata* | 286 | 4,955 | 302 |
| *N. vectensis* | 457 | 4,610 | 476 |
| *C. gigas* | 638 | 4,347 | 558 |
| *D. pulex* | 404 | 3,716 | 1,423 |
| *S. purpuratus* | 1,052 | 4,117 | 374 |
| *S. kowalevskii* | 392 | 4,707 | 444 |
| *D. rerio* | 2,197 | 2,818 | 528 |
| *A. aurita* | 696 | 3,885 | 962 |

**Table S10. Annotations of the significantly expanded gene families of *R. esculentum***

| **Cluster_ID** | **Nr product** |
| --- | --- |
| Cluster1021 | UDP-glucuronosyltransferase 2B9-like |
| Cluster1093 | solute carrier family 22 member 15-like |
| Cluster1142 | uncharacterized protein LOC107336737 |
| Cluster1281 | Skeletal organic matrix MAM and LDL-receptor 1 |
| Cluster1325 | Integrin alpha-4 |
| Cluster1327 | uncharacterized protein LOC107396698 |
| Cluster1381 | putative G-protein coupled receptor 157 |
| Cluster1476 | high-affinity choline transporter 1-like |
| Cluster1589 | G-protein coupled receptor |
| Cluster1592 | hypothetical protein AC249_AIPGENE25310 |
| Cluster1700 | tyrosine-protein kinase STK |
| Cluster1716 | cystatin-A |
| Cluster1735 | uncharacterized protein LOC107350583 |
| Cluster1739 | HMG domain-containing protein 3 |
| Cluster1807 | hypothetical protein AC249_AIPGENE26409 |
| Cluster1820 | uncharacterized protein LOC107352686 |
| Cluster2104 | multidrug and toxin extrusion protein 2-like |
| Cluster2174 | b(0,+)-type amino acid transporter 1-like |
| Cluster2237 | uncharacterized protein LOC107679731 |
| Cluster2293 | NADPH-dependent aldehyde reductase ARI1-like isoform X1 |
| Cluster2348 | Glutamate receptor 4 |
| Cluster2662 | uncharacterized protein LOC111105063 |
| Cluster5130 | P2X purinoceptor 7-like |
| Cluster5143 | hypothetical protein ASPCAL10255 |
| Cluster8777 | reverse transcriptase |
| Cluster9102 | Flavin-containing monooxygenase FMO GS-OX3 |
| Cluster9167 | uncharacterized protein LOC107350940 |

**Table S11. Annotations of the significantly contracted gene families of *R. esculentum***

| **Cluster_ID** | **Nr product** |
| --- | --- |
| Cluster1005 | ATP-dependent DNA helicase pif1-like |
| Cluster1006 | Dynein heavy chain 1, axonemal |
| Cluster1018 | Myotubularin-related protein 14 |
| Cluster1026 | zinc finger protein 208-like |
| Cluster1034 | RNA-directed DNA polymerase from transposon BS |
| Cluster1041 | sulfotransferase 1C2A-like |
| Cluster1053 | Uncharacterized protein K02A2.6 |
| Cluster1058 | E3 ubiquitin-protein ligase rnf213-alpha-like isoform X2 |
| Cluster1060 | histone H2A-like |
| Cluster1062 | uncharacterized protein LOC110252067 |
| Cluster1077 | acid-sensing ion channel 4-like |
| Cluster1102 | uncharacterized protein LOC102802199, partial |
| Cluster1112 | antagonist of like heterochromatin protein 1-like |
| Cluster1120 | RNA-directed DNA polymerase from mobile element jockey-like |
| Cluster1155 | NFX1-type zinc finger-containing protein 1 |
| Cluster1185 | matrix metalloproteinase-19-like |
| Cluster1213 | cysteine-rich secretory protein LCCL domain-containing 2-like |
| Cluster1222 | uncharacterized protein LOC110042058 isoform X3 |
| Cluster1293 | hamnose-binding lectin-like |
| Cluster1375 | Fibrocystin-L |
| Cluster1507 | disintegrin and metalloproteinase domain-containing protein 28-like |
| Cluster1576 | uncharacterized protein F54H12.2-like |
| Cluster1635 | hypothetical protein LOTGIDRAFT_205401 |
| Cluster1683 | piggyBac transposable element-derived protein 3-like |
| Cluster1724 | major royal jelly protein 5-like |
| Cluster1871 | hypothetical protein AC249_AIPGENE6507 |
| Cluster2043 | creatine kinase B-type-like isoform X2 |

**Table S12.** Abbreviations and full names of the genes used in this study.

| Gene abbreviation | Full name |
| --- | --- |
| ENPP5 | ectonucleotide pyrophosphatase/phosphodiesterase family member 5 |
| SLC35C2 | solute carrier family 35 member C2 |
| CNTNAP5 | contactin-associated protein 5 |
| TRPA1 | transient receptor potential cation channel subfamily A member 1 |
| ADAT1 | tRNA-specific adenosine deaminase 1 |
| GABARAPL2 | gamma-aminobutyric acid receptor-associated protein-like 2 |
| OSP | oxidative stress protein |
| EFCBP1 | N-terminal EF-hand calcium-binding protein 1 |
| DIO1 | type I iodothyronine deiodinase |
| PLA2 | phospholipase A2 |
| KIAA1468 | LisH domain and HEAT repeat-containing protein |
| YPT1 | putative GTP-binding protein ypt1 |
| GCSH | glycine cleavage system H protein |
| C18ORF63 | uncharacterized protein C18orf63 |
| TBC1D20 | TBC1 domain family member 20 |
| AARS | alanyl-tRNA synthetase |
| SAS10 | something about silencing protein 10 |
| PDPR | pyruvate dehydrogenase phosphatase regulatory subunit |
| DHOD | dihydroorotate dehydrogenase (quinone) |
| SLC47A1 | multidrug and toxin extrusion protein |
| Trpa1 | transient receptor potential cation channel subfamily A member 1 |

**Figure S1. *k*-mer estimation of the genome size of *R. esculentum***


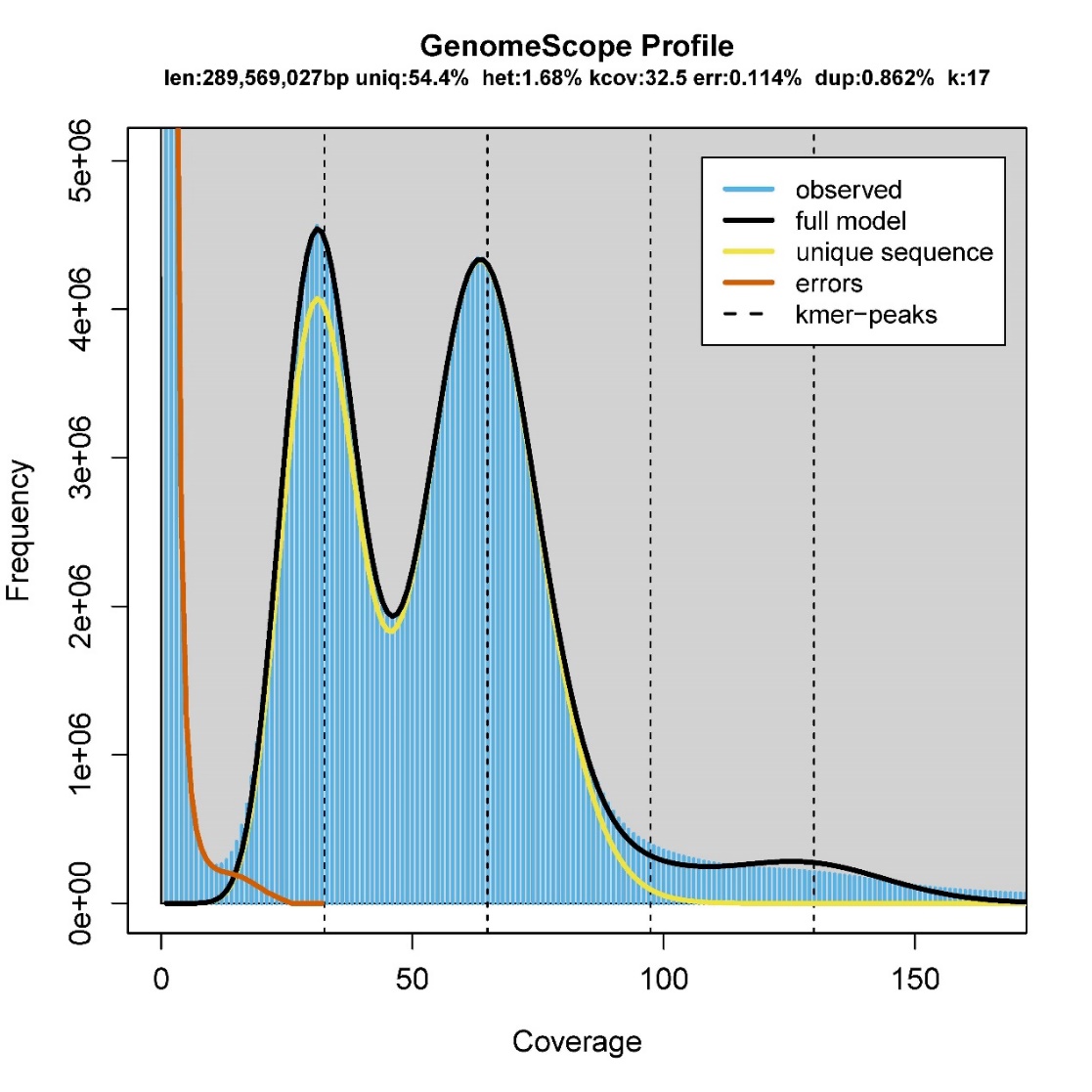


**Figure S2. Contig length distribution of the assembled genome of *R. esculentum***


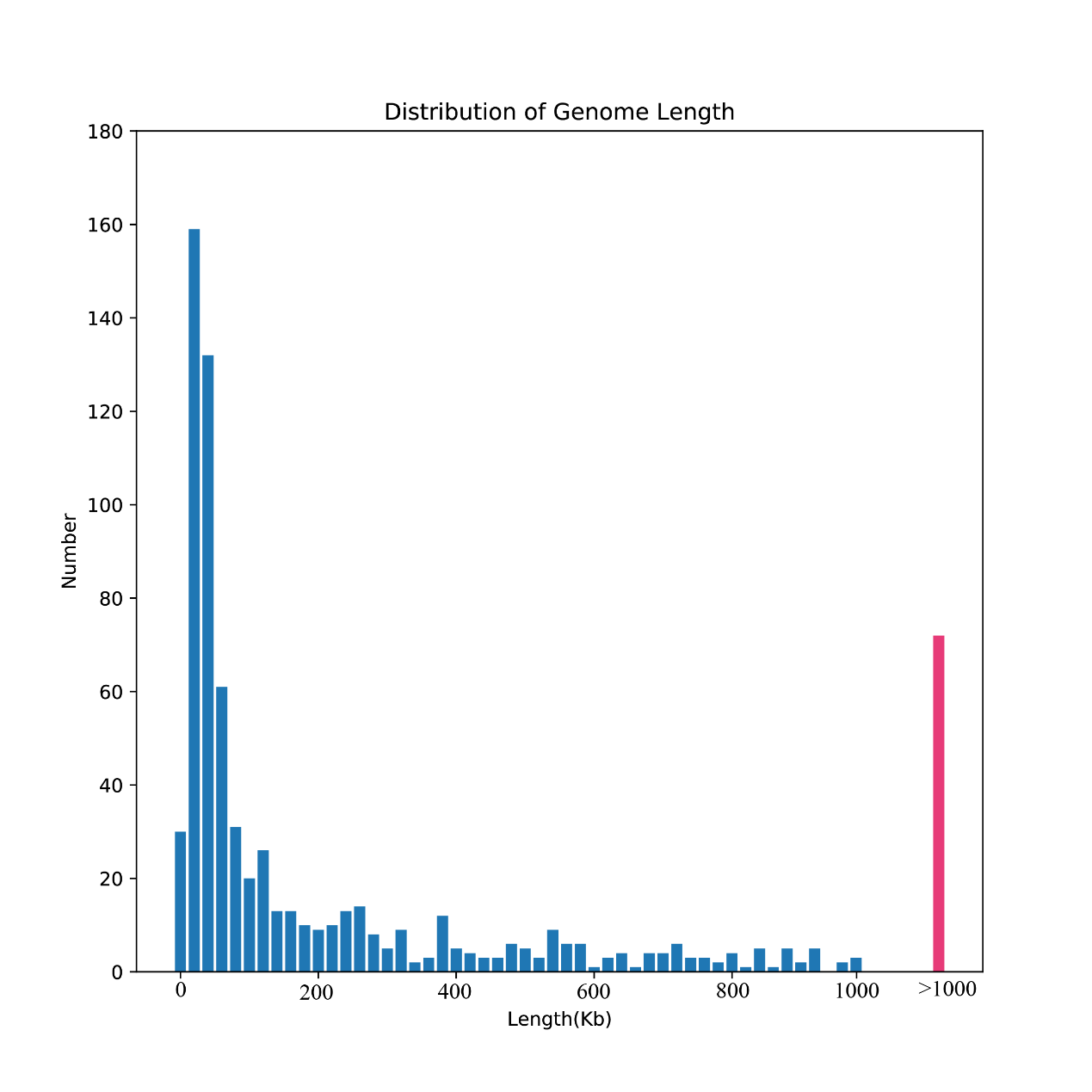


**Figure S3. GO analysis and functional classification of the protein coding genes in *R. esculentum*.**


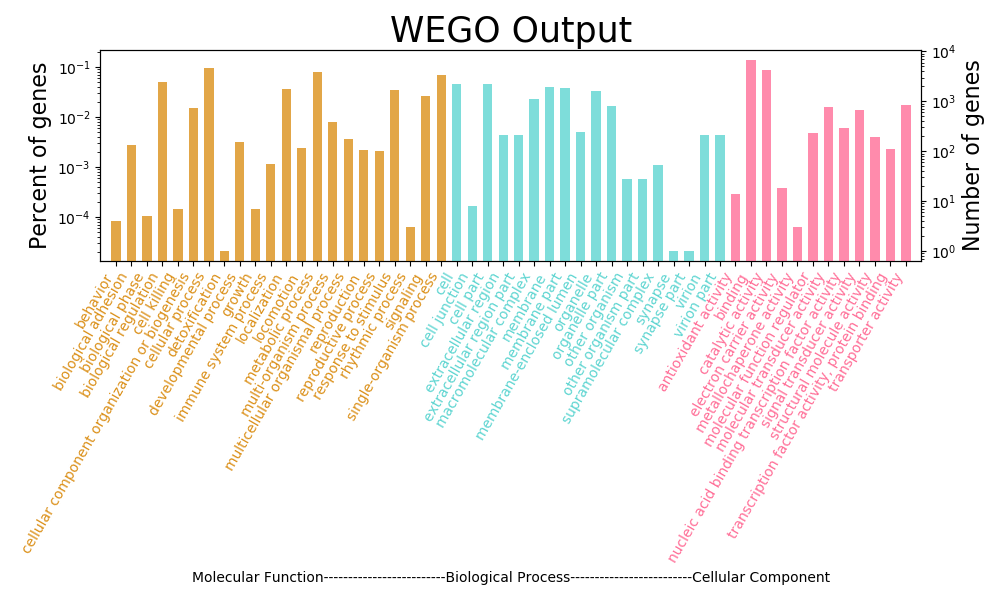


**Figure S4. KOG analysis and functional classification of the protein coding genes in *R. esculentum*.**


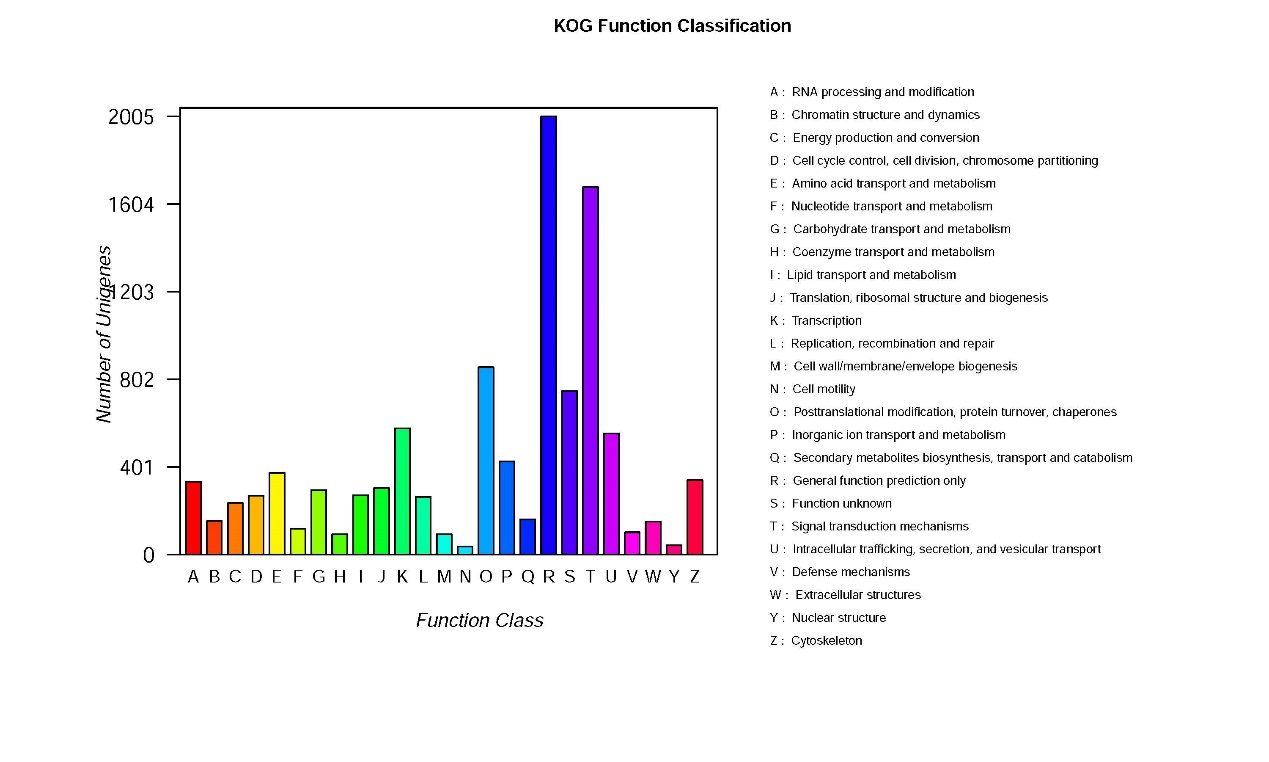


**Figure S5. Venn diagram of the statistics of the functional annotation.**


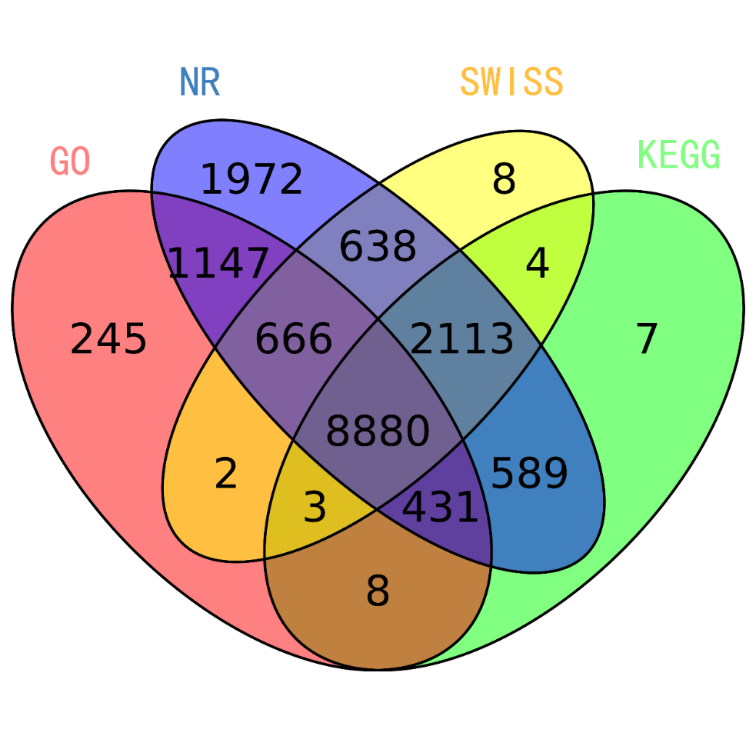


**Figure S6: Interaction frequency distribution of Hi-C links among chromosomes of *R. esculentum***


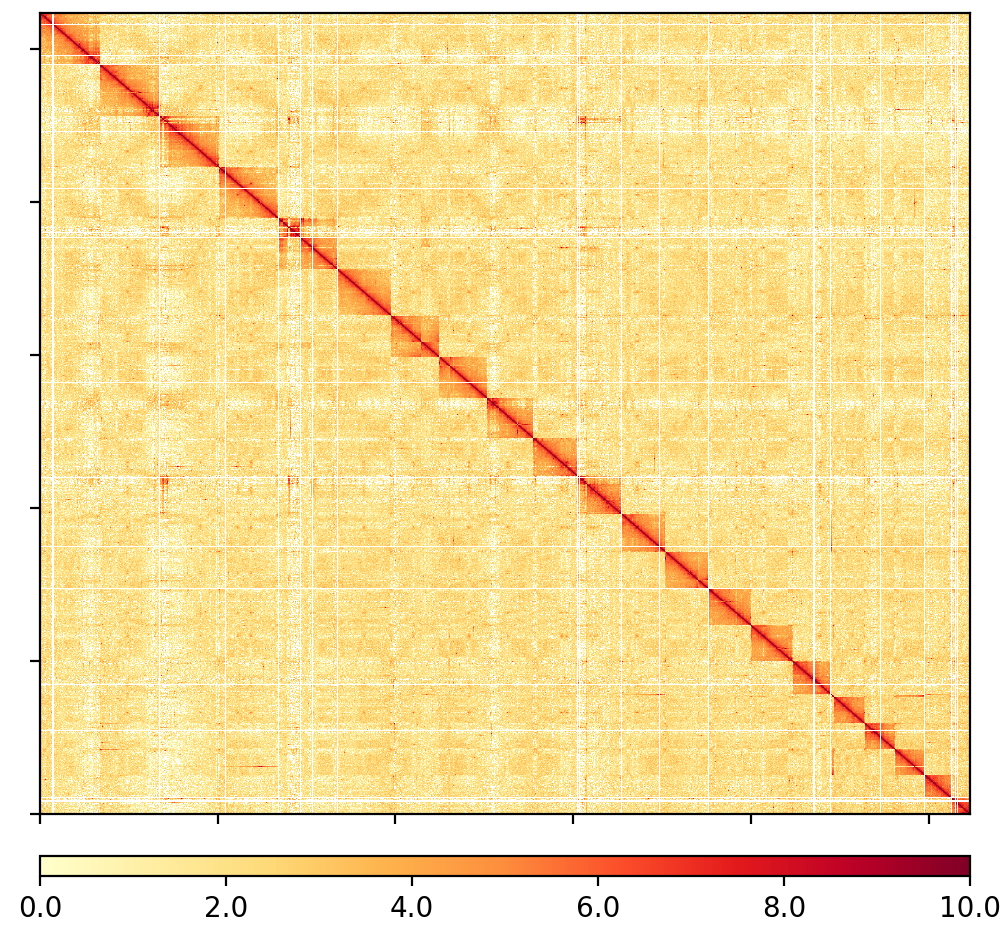

Supplement: giaa036_Supplemental_File [file giaa036_supplemental_file.zip › Supplementary tables and figures-0217.docx]
