# Supplementary material for: Chromosome-level reference genome of the jellyfish Rhopilema esculentum
Source: Gigascience. 2020 Apr 21;9(4):giaa036. doi: 10.1093/gigascience/giaa036 (PMC7172023; doi:10.1093/gigascience/giaa036)
Supplement: giaa036_Response_to_Reviewer_Comments_Original_Submission [file giaa036_response_to_reviewer_comments_original_submission.pdf]

Author's Response To Reviewer Comments

Close

Dear Dr. Hans Zauner and reviewers,

We have carefully read through the comments and made proper revisions according to the reviewers' valuable advice. We
hope that the revised submission can now be accepted for publication in GigaScience.

Reviewer #1
This is a good paper describing a very exciting/important dataset. The assembly is of high quality and the annotations anc
The venom analyses at the end of the paper provides a great example of how these data can be used to make impactful d
clear if passive voice was converted to active voice throughout. In that way it would be clear that the authors performed t
Below are specific comments with line numbers. NOTE: I received access to supplemental data after my initial review. A fe

1. Methods and results are intermingled throughout the paper. This serves to make the methods difficult to repeat and the
file is minimal and it is not possible to reconstruct the analyses from this document. Transcriptome assembly should be m
Response: This was done according to the writer's instruction of Data Note in Giga Science. We have tried our best to mak

2. Line 17: "Jellyfish belongs" .
-- should be "Jellyfish belong"
Response: This has been corrected in the revised manuscript.

3. Line 17: "occupies an important phylogenetic location together with Porifera as one of the earliest branching Metazoa li
-- This is confusing. Jellyfish did not diverge "with" Ctenophora. They also did not diverge "from" Ctenophora, which I assu
deleted.
Response: This sentence has been rewritten as "which occupies an important phylogenetic location in the early-branching

4. Line 19: "The jellyfish Rhopilema esculentum is important fishery resource in China."
-- This sentence is not grammatically correct. The first two sentences suffer from grammar issues. I will skip most gramm
Response: This sentence has been rewritten as "The jellyfish Rhopilema esculentum is an important fishery resource in Ch

5. Line 29: "that R. esculentum was less evolved than other species in Scyphozoa."
-- It would be more appropriate to say "that there are fewer inferred mutations in the R. esculentum genome as comparec
Response: We have revised the abstract and discussion according to your suggestion.

6. Line 39: "Jellyfish belongs..."
-- See comments on the first sentence (line 17) of the abstract above.
Response: Done

7. Line 43: "abundant fishery animals in China"
-- Not clear if this refers to the abundance of these animals in China seas or in China fish markets.
Response: This sentence means R. esculentum is one of the most abundant fishery animals in the seas. The corresponding

8. Line 50: "122,800,000 dollars/year of the profit in China"
-- US dollars?
Response: We have reconfirmed the profit value of R. esculentum in reference [5] and expressed clearly.

9. Line 56: "mainly concerning the increasing jellyfish blooms throughout the world"
-- I assume this is referring to the motivation of these scyphozoan genome projects. As one of the authors of [9], I disagr
Response: Done. It has been deleted from the revised manuscript.

10. Line 73: "For PacBio library construction, genomic DNA of R. esculentum"
-- it should be specified whether this is a different sample or the same sample as the one used for Illumina gDNA sequenc
Response: The sample used to conduct PacBio library construction was the same one used in next-generation sequencing.

11. Line 82: "We adapted a method using Jellyfish software based"
-- How does this estimation compare to the standard method implemented in GenomeScope (http://qb.cshl.edu/genomes
reported in more than 20 other papers.
Response: The further estimation of genome size has been added in the revised manuscript using GenomeScope. The com

12. The methods appear thorough, however repeating these analyses in full would be impossible without further informati
https://academic.oup.com/mbe/article/35/2/486/4644721#113627427. All analyses including the following should be incl
Response: All command lines used in this study are as follows. They have been also uploaded in GigaDB.

1) Genome estimation analysis

```
jellyfish count -m 17 -s 800M -t 48 -o kmer.hash -c 20 -C read1.fastq read2.fastq
jellyfish histo -l 1 -h 12000000000 -t 48 kmer.hash > kmer.hash.freq
jellyfish stats $3.hash > kmer.hash.stats
Rscript /gpfs/home/dongwei/biosoft/genomescope-master/genomescope.R kmer.hash.freq 17 150 kmer 1000
```

2) wtdbg2 analyses (Line 98)

```
wtdbg2 -i reads.fasta -fo haizhe -t 60 -x sq -g 300m
wtpoa-cns -t 60 -i haizhe.ctg.lay.gz -fo haizhe.raw.fa
```

3) Quiver analyses (Line 99)

```
pbalign --nproc 80 input_bam.fofn haizhe.raw.fa pbalign.bam
samtools faidx haizhe.raw.fasta
variantCaller -j8 --algorithm=arrow -r haizhe.raw.fa -o haizhe.cns.fasta pbalign.bam
```

4) Illumina clean genome data by in-house script (Line 101; This script should be made available -- for example in a softw

```
bwa index haizhe.cns.fa -p haizhe
bwa mem -t 80 -B 1 -O 1 -L 0,0 -k 17 -U 7 haizhe 1.fq 2.fq 2>bwa.log | samtools view -S -@ 60 -b - -o out.bam
samtools sort -@ 60 out.bam out.sorted
samtools flagstat out.sorted.bam > mapsum
bcftools mpileup -Q 0 -e 10 -f haizhe.cns.fa out.sorted.bam 2>bcftools.log |grep -v '#' |awk '$5!="<*>"' > out.raw.vcf
python call_snp.py out.raw.vcf > snp_indel.xls
python changeSNP.py haizhe.cns.fa snp_indel.xls > haizhe.correct.fa
```

5) Genome assembly that led to 760 contigs and N50 of 1.13Mb (line 102)

```
wtdbg2 -i reads.fasta -fo haizhe -t 60 -x sq -g 300m
wtpoa-cns -t 60 -i haizhe.ctg.lay.gz -fo haizhe.raw.fa
```

6) RepeatModeler (Line 104)

```
/bins/RepeatModeler/BuildDatabase -engine wublast -name Rhopilema Rhopilema.fasta;
/bins/RepeatModeler/RepeatModeler -engine wublast -database Rhopilema -pa 20;
```

7) RepeatMasker (Line 104)

```
/bins/RepeatModeler/BuildDatabase -engine wublast -name Rhopilema Rhopilema.fasta;
/bins/RepeatModeler/RepeatModeler -engine wublast -database Rhopilema -pa 20;
```

8) Augustus (Line 113)

```
augustus --genemodel=complete --gff3=on --species=haizhe haizhe.fasta > augustus.gff
```

9) GlimmerHMM (Line 114)

```
GlimmerHMM/bin/glimmerhmm_linux fasta.file -d ./arabidopsis/
```

10) SNAP15 (Line 114)

```
SNAP_train.pl
```

11) Genewise (Line 119)

```
genewise protein.fasta dna.fasta -both -gff
```

12) TopHat (sidenote: spelled wrong on page 123)

```
tophat -m 1 -o map --solexa-quals -a 5 --segment-length 20 --num-threads 20 genome.fasta RNAseq_R1.fq RNAseq_R2.f
```

13) Cufflinks (Line 124)

```
cufflinks -o transcripts.gtf -p 30 out.sort.bam
```

14) EvidenceModeler (Line 126)
perl evm.pl haizhe.fasta evm.gene.predict.gff3 evm.transcript.alignment.gff3 evm.homology.predict.gff3 haizhe_2018072

15) bwa (line 137)
bwa index haizhe.correct.fa -p haizhe
bwa mem -B 3 -O 3,3 -L 0,0 -t 80 haizhe 1.fq 2.fq 2>bwa.log | samtools view -S -@ 60 -b - -o out.bam
samtools sort -@ 60 out.bam out.sorted
samtools depth out.sorted.bam > out.sorted.depth

16) BUSCO (Line 138)
pythonrun_BUSCO.py -i haizhe_20180723.evm_final.rechoose.all.faa -o haizhe_euk
-l /bins/busco/dataset/euk/ -m proteins -c 40

17) HiCPlotter (Line 158)
HiCPlotter.py -f matrixfile -bed bedfile -r window -tri 1 -wg 1 -chr chromosomename -o outfileprefix -n figuretitle

18) OrthoMCL (Line 173)
orthomclFilterFasta my_orthomcl 10 20
orthomclInstallSchema config
orthomclBlastParser goodProteins.bsp my_orthomcl >> similarSequences.txt
orthomclLoadBlast config similarSequences.txt
orthomclPairs config orthomcl_pairs.log cleanup=yes
orthomclDumpPairsFiles config
orthomclMclToGroups Cluster 1000 < mclOutput > groups.txt

19) BLASTP (line175: I assume this is a step in OrthoMCL)
blastall -p blastp -d goodProteins.fasta -i goodProteins.fasta -F F -m8 -a 30 -e 1e-5 -o goodProteins.bsp

20) MCL (line177: assume this is a step in OrthoMCL)
mcl mclInput --abc -I 1.5 -o mclOutput

21) MUSCLE (line 181)
muscle -in gene.ffn -out gene.ffn.mus

22) RAxML (line 183)
perl Fasta2Phylip.pl only_snp.fasta phylip
raxmlHPC-PTHREADS -s phylip -n out.nwk -m PROTGAMMAJTT -p 12345 -T 20 &
raxmlHPC-PTHREADS -s phylip -n out_boot.nwk -m PROTGAMMAJTT -p 12345 -T 20 -b 12345 -# 100
raxmlHPC-PTHREADS -f b -s phylip -m PROTGAMMAJTT -n haizhe_20170801.nwk -z RAxML_bootstrap.out_boot.nwk -t RA

23) CAFE (line 188)
perl cafe_pipe.pl geneFamily.tab time_tree.nwk --output cafe --filter

24) ParaAT and KaKs_Calculator (Line 211)
ParaAT.pl -homolog Orthogroups.tsv -aminoacid AA.fasta -nuc CDS.fasta -processor proc.txt -output kaks -format axt -ka

13. Line 106: "the low complexity or simple repeats were not masked"
-- What WAS masked?
Response: The interspersed repeats (SINEs/LINEs/LTR elements/DNA elements/Unclassified) were masked and the detaile
of the low complexity or simple repeats could be found in the genes”.

14. Line 118: "aligned to the assembled genome"
-- how was it aligned? Provide software, version, and command lines.
Response: Genewise (version 2.2.0) was used to align and generate gene structures based on the homology alignments. T

15. Line 135: Quality assessment
-- It is impossible to judge the quality of the cufflinks transcriptome and the predicted gene models from this paper. Pleas
Response: Yes, we are pleased to provide BUSCO scores. Please see Supplementary Table S5.

16. Line 129: "All the gene sequences were mapped"
-- how were they mapped? Provide software, version, and command lines.

Response: Done. We have revised this sentence to “All the gene sequences were searched using BLASTP with an E-value c

17. Line 132: "could be mapped to at least one database"
-- what was criteria used for successful mapping?
Response: The criteria used for mapping is E-value<1e-5 and this description has been added in the context.

18. Line 138: "The percentage of aligned reads was estimated to be 99.81%."
-- what was criteria used for successful mapping?
Response: The criteria used for mapping is “bwa mem -t 80 -B 1 -O 1 -L 0,0 -k 17 -U 7”.

19. Line 141: "C: 97.0% [S: 92.1%, D: 5.0%], F: 1.7%, M: 1.3%, n: 303, wherein C, S, D, F,"
-- this would be more clear as a table with the important stats mentioned in the text.
Response: The BUSCO data was provided in the Supplementary Table S4.

20. Line 151: "essentially as previously described"
-- "essentially" implies that there were variations from the previously described method. Any deviations from the previous
Response: The method used in this study is exactly the same as described in reference 28 and the word “essentially” has

21. Line 152: "MboI was used as the restriction enzyme"
-- there needs to be more information here. For example. "The DNA was digested by MboI for X hours at X°C."
Response: The DNA was digested overnight (12h) by 200U of the restriction enzyme MboI at 37 oC with shaking. The info

22. Line 158: "Finally, 260.17 Mb (94.46%) of the assemblies were anchored onto 21 pseudochromosomes"
-- Is this the output of HiCPlotter or a different unspecified step. It is not clear.
Response: This is the output of 3D-DNA. To make it clearer, we have revised this sentence to “The 3D-DNA was used to a
Karyotype (2n=42) of R. esculentum [32] (Fig. 2, Supplementary Fig. S6 and Supplementary Table S6). This chromosome

23. Line 172: "All data of the other 12 species were obtained from Ensembl or NCBI"
-- A supplemental table with sources of all data (including accessions or URLs) should be supplied.
Response: Done. Please see Supplementary Table S7.

24. Line 180: "selected for further alignment using MUSCLE"
-- This sounds like MUSCLE was used to select the orthogroups, which doesn't make sense. This should be clarified.
Response: To make it clearer, we have revised this sentence to “A total of 335 selected single-copy orthologous genes we

25. Line 182: "alignment by an in-house Perl script."
-- This script should be made publicly available either as a text file in the supplement or on a code repository like GitHub.
Response: Done. We have made publicly available as text files in the supplement on GigaDB.

26. Line 182: " maximum likelihood phylogeny was reconstructed using RAxML"
-- There is no information regarding model, partitioning, etc. If partitioning was used, the partition file should be supplied.
Response: We have made publicly available as text files in the supplement on GigaDB. It is as follows.

```
perl Fasta2Phylip.pl only_snp.fasta phylip
raxmlHPC-PTHREADS -s phylip -n out.nwk -m PROTGAMMAJTT -p 12345 -T 20 &
raxmlHPC-PTHREADS -s phylip -n out_boot.nwk -m PROTGAMMAJTT -p 12345 -T 20 -b 12345 -# 100
raxmlHPC-PTHREADS -f b -s phylip -m PROTGAMMAJTT -n haizhe_20170801.nwk -z RAxML_bootstrap.out_boot.nwk -t RA
```

27. Line 183: "The results supported the view that R. esculentum and H. vulgaris are sister groups."
-- This is wrong. Figure 4 has A. aurita as sister to R. esculentum
Response: The results supported the view that R. esculentum together with A. aurita and H. vulgaris are sister groups. Th

28. Line 189: "27 and 27 gene families"
-- typo?
Response: To avoid misleading, the sentence has been re-written as “Twenty-seven gene families were found to be signifi

29. Line 200: "244 unique gene families that could be annotated in NR"
-- How could they be annotated? What software? What cutoff?
Response: Blastp was used for gene annotation with a cutoff E-value of 1e-5 and the corresponding description has been

30. Line 202: "were annotated with the proteins of the species in Anthozoa"
-- How were they annotated?
Response: The sentence has been re-written as “It was surprising that more than half of those (136 unique gene families)

31. Line 203: "This was also supported by the results of phylogenetic analysis among the 13 species"
-- what was supported. I don't understand.
Response: Considering the problem you mentioned, this sentence has been corrected as “This result implied that some ge
supported by the phylogenetic analysis of the 13 species, in which R. esculentum was found to exhibit fewer gene gains (3

32. Line 204: "Compared to H. vulgaris and Aurelia, R. esculentum had fewer gene gains (331) and fewer gene losses (29
-- Sentence should include the number of gains and losses in H. vulgaris and Aurelia.
Response: Combining the problem pointed out in line 203 of the original manuscript, this sentence has been corrected as
vulgaris. This was also supported by the phylogenetic analysis of the 13 species, in which R. esculentum was found to exh

33. Line 206: "were kept by Anthozoa species and R. esculentum, but were lost in other Scyphozoa species."
-- the entire quote above would be clearer and more accurate if replaced by "were lost in Aurelia and H. vulgaris."
Response: Thanks for your advice. The sentence has been revised as you suggested.

34. Line 208: "To further explore this hypothesis..."
-- What hypothesis? There has not been one stated.
Response: Please see the next respondence.

35. Line 208: "There were 7542, 7864, 7611 and 6141 orthogroups found in Re-Hv, Aa-Hv, Nn-Hv and Cx-Hv, 10213 resp
-- How were paralogs treated? How were sequences aligned? This very rough positive selection analysis is not convincing.
Response: The sequences were analyzed using OrthoMCL - ParaAT - KaKs_Calculator pipeline. The results are supposed to
resources available. As you suggested, the distant phylogenetical species may lead to rough results. Thus, we deleted this

36. Line 219: "Jellyfish, one of the main subgroups of Cnidaria, is one of the oldest extant lineages of venomous animals"
-- This sentence implies that Jellyfish are older than other cnidarian groups. I think the authors were trying to say that cni
ancestor (e.g. from the last common ancestor of all animals). This sentence could be deleted.
Response: The corresponding sentence has been revised.

37. Line 235: "Second, according to the gene annotations of NR, Uniprot and Tox-Prot, the genes annotated consistently w
-- This sentence is not clear. Active voice may help. Also, if there is explicit criteria being used, it should be stated clearly.
Response: It has been revised as follows: “In step 2, according to the best hits of gene annotations of NR, Uniprot and To

38. Line 237: "Third, to make the pool of venom-related genes more complete, we screened all the genes predicted in the
-- This too is unclear. Also, manual curation is difficult to repeat, but if it must be manual, than detailed criteria should be
Response: The manuscript has been revised as follows: “In step 3, to make the pool of venom-related genes more comple
considered as toxin-like genes”.

39. Line 253: "were observed in the tentacles of scyphozoan and cubozoan species "
-- Assuming these observations were not made as part of this study, it would be more clear to say "have been observed i
Response: This description is indeed not part of this study and the corresponding presentation has been corrected as you

40. Line 256" "suggesting their important roles during evolution."
-- It is not clear why this result suggests "important roles during evolution." This should be clarified or removed.
Response: The presentation has been deleted for the context.

41. Line 259: "were highly abundant in cubozoan venoms"
-- Assuming these observations were not made as part of this study, it would be
more clear to say "have been observed in high abundance in cubozoan venoms"
Response: The sentence has been re-written as you suggested.

42. Line 259: "were also reported"
-- "have been reported"
Response: The mistake has been revised in the context.

43. Line 265: "Three new toxin-like genes that have not been previously reported in jellyfish were..."
-- Prothrombin was reported in Cassiopea in Ohdera et al. 2019 (Table S4 -- line 73).
Response: The corresponding result and description have been removed from the manuscript.

44. Line 274: "was mainly identified in the honeybee"
-- grammar issue makes this confusing.

Response: This sentence has been reformed as “It was mostly found in honeybee” for better understanding.

45. Line 275: "The new toxin-like genes identified in this study will provide insight into the complex composition of jellyfis
-- as written this sentence is kind of a throwaway. It would be better to mention that: the discovery of these toxin-coding
Response: We have revised this sentence as you suggested.

46. Line 281: "The different composition of venom may account for the different symptoms after sting."
-- what are the different symptoms?
Response: According to the present analysis, stinging by Rhopilema hispidum would cause immediate strong burning sens
result in a very painful sting with a strong burning sensation. The reference for these has been cited in the revised manus
context.

47. Line 291: "It was also reported that neighboring genes tend to be co-expressed rather than expressed by chance"
-- This is misleading. This is an Arabidopsis paper. At least find a paper that shows this in animals and mention explicitly t
Response: The related studies have been cited in the manuscript.

48. Line 294: "Further studies are needed to clarify its specific functions."
-- This is not a great way to end the paper. Why not add a paragraph conclusion saying how these new data can be used i
Response: A new ending paragraph has been added in the context.

49. Table 3: "The green and yellow boxes indicate the identified and unidentified toxin-like genes respectively"
-- "unidentified toxin-like genes" is confusing. It would be more clear to make yellow boxes white (or unfilled) and state th
Response: The boxes and the table illustration of Table 3 have been revised as you suggested.

50. Figure 1: "Picture of a jellyfish R. esculentum captured from Yingkou, Liaoning Province, China."
-- "Photograph of Rhopilema esculentum collected from Yingkou, Liaoning Province, China." Either include approximate siz
-- Also, Figure 1 is not (but should be) referenced in the main text.
Response: Figure 1 has been referenced in the revised manuscript.

Reviewer #2
This manuscript presents a chromosome-level genome assembly of the edible scyphozoan jellyfish Rhopilema esculentum,

Major comments:

1. Several relevant methodological details are missing, and would need to be provided: The origin of the biological materi
so, how many individuals/piece of tissues were used for each extraction?
Response: Some of the details were included in the NCBI database. The experimental individuals are all cultured animals.
used for genomic sample, and one another individual was used for Hi-C analysis. For transcriptomic analysis, a total of 60

2. Which procedures were performed in order to avoid contamination? Were the organisms starved prior to extraction? We
Response: The experimental individuals were starved for two days prior to extraction, and sterile water was used to treat

3. For RNAseq experiments - please provide details about the sequencing. Was it pair-ended? How many replicates? This i
Response: This study was carried out by PE-250 sequencing utilizing Illummina HiSeq2500 platform. Three replications we

4. The phylogenetic methods used to produce figure 4 should be provided. Which model was used for the RaXML analyses
Response: PROTGAMMAJTT model was used for RaXML analyses. The divergence times of Mnemiopsis leidyi vs. Aurelia au
each node in phylogenetic tree. These have been added in the manuscript. The command lines are as follows.

RaXML command :

```
perl Fasta2Phylip.pl only_snp.fasta phylip
raxmlHPC-PTHREADS -s phylip -n out.nwk -m PROTGAMMAJTT -p 12345 -T 20 &
raxmlHPC-PTHREADS -s phylip -n out_boot.nwk -m PROTGAMMAJTT -p 12345 -T 20 -b 12345 -# 100
raxmlHPC-PTHREADS -f b -s phylip -m PROTGAMMAJTT -n haizhe_20170801.nwk -z RAxML_bootstrap.out_boot.nwk -t RA
```

fossil calibration :

```
MRCA AA Mnemiopsis_leidyi Aurelia_aurita;
MRCA BB Strongylocentrotus_purpuratus Aurelia_aurita;
MRCA CC Danio_rerio Nematostella_vectensis;
CONSTRAIN taxon=AA min_age=698 max_age=1047;
CONSTRAIN taxon=BB min_age=617 max_age=902;
CONSTRAIN taxon=CC min_age=641.8 max_age=1250;
```

Café command :
perl cafe_pipe.pl geneFamily.tab time_tree.nwk --output cafe --filter

5. All the scripts mentioned in the main text should be provided as supplementary information (e.g. L102, L182).
Response: All scripts have been made available on GigaDB.

6. The statement that R. esculentum evolved less than other scyphozoan species is not very convincing. This would need t
schyphozoans, relies on the comparison of too few species. I would also suggest entirely removing the part on positive se
Response: As you suggested, our analysis was only focus on several Scyphozoa species, not all Scyphozoa species. Thus,
resources available. As you suggested, the distant phylogenetical species may lead to rough results. Thus, we deleted this

Other comments:
7. I would suggest removing the parentheses in the title.
Response: The parentheses have been deleted from the title.

8. L17-18: The phylogenetic position of ctenophore is still highly debated. I would suggest removing "after their divergenc
Response: The debatable description has been deleted from the context.

9. L19: add 'an': "is an important".
Response: Done.

10. L23-24. The sentence "A total… respectively." seems to me unnecessary for an abstract. I would suggest removing it.
Response: Done.

11. L32: replace "we first finished" with "we generated a"
Response: Done.

12. L41: add the year of species description "1891" after "Kishinouye".
Response: Done.

13. L42: lowercase for "Class" - also L198.
Response: Done.

14. L47: rephrase "their harmfulness to industry and the community in blooms".
Response: The sentence has re-written as "In contrast to many other jellyfish species that have drawn public attention be

15. L48: replace "for" with "because of" and provide a reference in support of that claim.
Response: The word has been revised in the main context and the reference has been added.

16. L54: replace "Aurelia and Aurelia aurita" with "Aurelia aurita".
Response: Done.

17. L53-56. Other recent releases of genome assembly of non-scyphozoan medusozoans - cubozoan (Morbakka virulenta,
Response: New citation has been made in the manuscript and the detailed information has been added both in the contex

18. L56. I would delete "mainly concerning the increasing jellyfish blooms throughout the world" as the recent publications
Response: This inappropriate description has been removed from the manuscript.

19. Rhopilema esculentum is often referenced in the text simply as "jellyfish". To avoid confusion, I would replace "jellyfis
Response: Done.

20. L151. "were performed essentially as previously described [28]." Please detail the difference with the published protoc
Response: The protocol used in this study is exactly same as described in reference 28. To make it clear, the word "essen

21. L198. Replace "Aurelia" with "A. aurita".
Response: Done.

22. L219. Replace "Jellyfish" with "Medusozoa".
Response: Considering of the suggestion from you and the other two reviewers, this sentence has been removed from the

23. L236. "the genes annotated… toxin-like genes". This part of the sentence is unclear, please reformulate.
Response: The sentence has been re-written as "In step 2, according to the best hits of gene annotations of NR, Uniprot a

24. L258. "Two copies… found". This sentence is unclear, please reformulate.
Response: The sentence has been re-organized as "Two copies of "jellyfish toxin", also called cubozoan-related porins, we

25. L281. "They are known to…. neurotoxins". A reference needs to be added here.
Response: The reference information has been added in the context.

26. L283. "Some" - how many?
Response: Eight toxin-like genes were located closely on contig 521 and the exact number has been added in the manusc

27. L285. The term "associated" is too imprecise and should be replaced. Do you mean: found on the same scaffold? If so
Response: The sentence has been re-written as "The functions of toxin-like genes in the hub included phospholipase A2 ac

28. L290. It is not true that "genes located closely in genome are always involved in related functions and expressed in sir
Response: The corresponding sentence has been deleted.

29. L292. I would avoid the passive mode and replace "It was suggested" with "We suggest".
Response: Done.

30. L294. Remove "the". "and function of jellyfish" is unclear - reformulate or delete. Please also replace "its" with "their".
Response: To make it clear, "jellyfish" has been replaced by "R. esculentum" and the other two grammar issues have been

31. Figure 2F: How were calculated those "major interchromosomal relationships"? How to interpret them? Please also pro
Response: The major interchromosomal relationships was conducted utilizing lastZ with the following command line:
lastz chr1.2bit chr2.2bit --notransition --nogapped --step=40 --format=general:name1,start1,end1,length1,name2,start2
Figure 2 has been replaced by a new version. The scale bars were provided at the left bottom of the figure.

32. Figure 3. Please describe in the figure legend the meaning of the numbers and numbers between brackets shown on t
Response: The detailed description has been added in the figure illustration.

Reviewer #3
In this paper, Li et al. describe the genome of the jellyfish Rhopilema. This is the first chromosome-level assembly of a sc

1. A small (but important) point. In several places the authors discuss the Rhopilema genome as being "less evolved" than
Response: Thank you for your suggestion. Combining your and other reviewers' opinion, "less evolved" has been replaced

2. The authors do not include any comparative macro- or microsynteny analyses, which is standard in a genome paper. G
less derived nature of the Rhopilema genome, as gene gain/loss analysis appears to.
Response: Actually, we did synteny analyses with other Scyphozoans, and the results were disordered and not adequate f
level genome assembly by now. Instead, we conducted synteny analysis within R. esculentum, and the results were showe

3. There is no description on how the authors dated their molecular clock for Figure 4, other than they used "the phylogen
more information on how their tree is dated and consider methodologies that bring their clock closer in line with more tho
Response: PROTGAMMAJTT model was used for RaXML analyses. The divergence times of Mnemiopsis leidyi vs. Aurelia au
each node in phylogenetic tree. These have been added in the manuscript.

The command lines are as follows.

```
r8s -b -f r8s-contig.txt > r8s_out.txt
perl Fasta2Phylip.pl only_snp.fasta phylip
raxmlHPC-PTHREADS -s phylip -n out.nwk -m PROTGAMMAJTT -p 12345 -T 20 &
raxmlHPC-PTHREADS -s phylip -n out_boot.nwk -m PROTGAMMAJTT -p 12345 -T 20 -b 12345 -# 100
raxmlHPC-PTHREADS -f b -s phylip -m PROTGAMMAJTT -n haizhe_20170801.nwk -z RAxML_bootstrap.out_boot.nwk -t RA
fossil calibration :
MRCA AA Mnemiopsis_leidyi Aurelia_aurita;
MRCA BB Strongylocentrotus_purpuratus Aurelia_aurita;
MRCA CC Danio_rerio Nematostella_vectensis;
CONSTRAIN taxon=AA min_age=698 max_age=1047;
CONSTRAIN taxon=BB min_age=617 max_age=902;
CONSTRAIN taxon=CC min_age=641.8 max_age=1250;
Café command :
perl cafe_pipe.pl geneFamily.tab time_tree.nwk --output cafe --filter
```

The content of the config file of r8s is as follows:
#NEXUS
Begin trees;
tree STATE_0 = [&R]
(((((((Aurelia_aurita:0.12013130,Rhopilema_esculentum:0.12586633)1.0000:0.17380521,Hydra_vulgaris:0.37848113)1.
ccoglossus_kowalevskii:0.25047315,Strongylocentrotus_purpuratus:0.33882824)1.0000:0.06040144)0.7900:0.0250785
End;
BEGIN r8s;
BLFORMAT lengths=persite nsites=52236 ultrametric=no;
COLLAPSE;
MRCA AA Mnemiopsis_leidyi Aurelia_aurita;
MRCA BB Strongylocentrotus_purpuratus Aurelia_aurita;
MRCA CC Danio_rerio Nematostella_vectensis;
CONSTRAIN taxon=AA min_age=698 max_age=1047;
CONSTRAIN taxon=BB min_age=617 max_age=902;
CONSTRAIN taxon=CC min_age=641.8 max_age=1250;
set checkgradient=yes smoothing=100;
divtime method=pl algorithm=tn;
showage;
set penalty=add checkgradient=no;
DIVTIME method=PL fossilconstrained=yes crossv=yes cvstart=0 cvinc=0.50 cvnum=6 algorithm=tn;
DIVTIME method=PL fossilfixed=yes crossv=yes cvstart=0 cvinc=0.50 cvnum=6 algorithm=tn;
describe plot=chrono_description;
END;

4. This last suggestion is optional. The authors note a set of venom genes lie close together in the genome and posit that
looks to be of high quality) and only use it for gene modelling. The paper could be much more compelling if that data were
Response: The RNA-Seq data we collected will be analyzed together with more sequencing data in the future to address a

Overall this is a well-written manuscript with a quality dataset. I look forward to seeing this paper published once some ad

References:

Dohrmann M, Wörheide G. 2017. Dating early animal evolution using phylogenomic data. Sci Rep 7:3599.

dos Reis M, Thawornwattana Y, Angelis K, Telford MJ, Donoghue PC, Yang Z. 2015. Uncertainty in the timing of origin of a

Response: We have gone through the references you kindly provided and carefully revised the description of how to date

Yours Sincerely,

Zunchun Zhou, Ph.D.
Professor and Director of
Liaoning Ocean and Fisheries Science Research Institute

Close
