# Supplementary material for: Chromosome-level reference genome of the jellyfish Rhopilema esculentum
Source: Gigascience. 2020 Apr 21;9(4):giaa036. doi: 10.1093/gigascience/giaa036 (PMC7172023; doi:10.1093/gigascience/giaa036)
Supplement: giaa036_Response_to_Reviewer_Comments_Revision_1 [file giaa036_response_to_reviewer_comments_revision_1.pdf]

Author's Response To Reviewer Comments

Close

We greatly appreciate the editor and the reviewers for your time and efforts to improve our manuscript. Please see below for our point-by-point replies to the comments.

Editor:
1. When you mention the species sampling in the methods section, please also include the NCBI taxon ID for the species (I believe it is NCBI:txid499914, but please double check).
Authors' respondence: The NCBI taxon ID has been added.

2. Please include a citation to your upcoming GigaDB dataset (including the DOI link) to your reference list, and cite this in the data availability section.
Authors' respondence: The citation has been added and was cited in the data availability section.

Reviewer #2:
1. The sentence "The phylogenic results supported the view that R. esculentum, A. aurita and H. vulgaris are sister groups" is however still misleading. It could be replaced by "The phylogenetic results supported the monophyly of R. esculentum, A. aurita and H. vulgaris".
Authors' respondence: The manuscript has been revised as the reviewer suggested.

Close
