# Supplementary material for: Chromosome-level reference genome of the jellyfish Rhopilema esculentum
Source: Gigascience. 2020 Apr 21;9(4):giaa036. doi: 10.1093/gigascience/giaa036 (PMC7172023; doi:10.1093/gigascience/giaa036)
Supplement: giaa036_Reviewer_1_Report_Original_Submission — Joseph F. Ryan -- 10/31/2019 Reviewed [file giaa036_reviewer_1_report_original_submission.pdf]

Reviewer Report

Title: Chromosome-level reference genome of the jellyfish Rhopilema esculentum

Version: Original Submission **Date:** 10/31/2019

Reviewer name: Joseph F. Ryan

Reviewer Comments to Author:

This is a good paper describing a very exciting/important dataset. The assembly is of high quality and the annotations and analyses are thorough. These data will make a great contribution to several fields of biology, especially evolutionary biology, and venom-omics. The scope of the paper is appropriate for a data note. The figures look nice (especially Figure 1!). The venom analyses at the end of the paper provides a great example of how these data can be used to make impactful discoveries.

There are grammar issues throughout the paper (not terrible but substantial). The paper would benefit greatly from being edited by an editor whose first language is English, with an eye towards grammar. The paper would be more clear if passive voice was converted to active voice throughout. In that way it would be clear that the authors performed the work being described (it is often ambiguous). For example on line 178: "A set of 32,138 gene families were eventually identified" would be more clear as "We identified a set of 32,138 gene families..."

Methods and results are intermingled throughout the paper. This serves to make the methods difficult to repeat and the results hard to follow. I highly recommend separating them. Regarding the methods, it is very important that command lines and information associated with each analyses be made available in the supplemental data. The current 'readme.txt' file is minimal and it is not possible to reconstruct the analyses from this document. Transcriptome assembly should be made made available in the supporting data. The availability of assembled datasets should be mentioned in the section titled "Availability of supporting data."

Below are specific comments with line numbers:

NOTE: I received access to supplemental data after my initial review. A few of these comments may be addressed by current supplemental data.

Line 17: "Jellyfish belongs"

-- should be "Jellyfish belong"

Line 17: "occupies an important phylogenetic location together with Porifera as one of the earliest branching Metazoa lineages after their divergence with Ctenophora."

-- This is confusing. Jellyfish did not diverge "with" Ctenophora. They also did not diverge "from" Ctenophora, which I assume was the intended meaning (the diverged from the last common ancestor of ctenophora and jellyfish). It feels strange to refer to "Porifera" especially if "Placozoa" is not also referred to. This first sentence has a lot of problems and could be deleted.

Line 19: "The jellyfish Rhopilema esculentum is important fishery resource in China."

-- This sentence is not grammatically correct. The first two sentences suffer from grammar issues. I will skip most grammar issues moving forward, but the issues are throughout the manuscript and should be addressed.

Line 29: "that R. esculentum was less evolved than other species in Scyphozoa."
-- It would be more appropriate to say: "that there are fewer inferred mutations in the R. esculentum genome as compared to other available scyphozoan genomes and therefore that it might be evolving slower than those of other scyphozoans."
Line 39: "Jellyfish belongs..."
-- See comments on the first sentence (line 17) of the abstract above.
Line 43: "abundant fishery animals in China"
-- Not clear if this refers to the abundance of these animals in China seas or in China fish markets.
Line 50: "122,800,000 dollars/year of the profit in China"
-- US dollars?
Line 56: "mainly concerning the increasing jellyfish blooms throughout the world"
-- I assume this is referring to the motivation of these scyphozoan genome projects. As one of the authors of [9], I disagree with this being the motivation. This phrase could be deleted.
Line 73: "For PacBio library construction, genomic DNA of R. esculentum"
-- it should be specified whether this is a different sample or the same sample as the one used for Illumina gDNA sequencing.
Line 82: "We adapted a method using Jellyfish software based"
-- How does this estimation compare to the standard method implemented in GenomeScope (http://qb.cshl.edu/genomescope/)? The details of the estimating method used in this publicaiton should be provided as code or a series of command lines (in a supplement or public repository like GitHub). Or just report the results from GenomeScope, which have been reported in more than 20 other papers.
The methods appear thorough, however repeating these analyses in full would be impossible without further supplied information. In order to make the work repeatable, please include ALL command lines (including infiles or URLs where infiles can be found) and software versions in a supplemental document. There is an excellent example in the supplement linked here:
https://academic.oup.com/mbe/article/35/2/486/4644721#113627427
All analyses including the following should be included in this document:

1. Genome estimation analysis
2. wtdbg2 analyses (Line 98)
3. Quiver analyses (Line 99)
4. Illumina clean genome data by in-house script (Line 101; This script should be made available -- for example in a software repo like Github)
5. Genome assembly that led to 760 contigs and N50 of 1.13Mb (line 102)
6. RepeatModeler (Line 104)
7. RepeatMasker (Line 104)
8. Augustus (Line 113)
9. GlimmerHMM (Line 114)
10. SNAP15 (Line 114)
11. Genewise (Line 119)
12. TopHat (sidenote: spelled wrong on page 123)
13. Cufflinks (Line 124)

14. EvidenceModeler (Line 126)
15. bwa (line 137)
16. BUSCO (Line 138)
17. HiCPlotter (Line 158)
18. OrthoMCL (Line 173)
18a. --BLASTP (line175: I assume this is a step in OrthoMCL)
18b. --MCL (line177: assume this is a step in OrthoMCL)
19. MUSCLE (line 181)
20. RAxML (line 183)
21. CAFE (line 188)
22. ParaAT and KaKs_Calculator (Line 211)

Line 106: "the low complexity or simple repeats were not masked"
-- What WAS masked?
Line 118: "aligned to the assembled genome"
-- how was it aligned? Provide software, version, and command lines.
Line 135: Quality assessment
-- It is impossible to judge the quality of the cufflinks transcriptome and the predicted gene models from this paper. Please provide BUSCO scores for both.
Line 129: "All the gene sequences were mapped"
-- how were they mapped? Provide software, version, and command lines.
Line 132: "could be mapped to at least one database"
-- what was criteria used for successful mapping?
Line 138: "The percentage of aligned reads was estimated to be 99.81%."
-- what was criteria used for successful mapping?
Line 141: "C: 97.0% [S: 92.1%, D: 5.0%], F: 1.7%, M: 1.3%, n: 303, wherein C, S, D, F,"
-- this would be more clear as a table with the important stats mentioned in the text
Line 151: "essentially as previously described"
-- "essentially" implies that there were variations from the previously described method. Any deviations from the previously described study should be provided.
Line 152: "MboI was used as the restriction enzyme"
-- there needs to be more information here. For example. "The DNA was digested by MboI for X hours at XÂ°C."
Line 158: "Finally, 260.17 Mb (94.46%) of the assemblies were anchored onto 21 pseudochromosomes"
-- Is this the output of HiCPlotter or a different unspecified step. It is not clear.
Line 172: "All data of the other 12 species were obtained from Ensembl or NCBI"
-- A supplemental table with sources of all data (including accessions or URLs) should be supplied.
Line 180: "selected for further alignment using MUSCLE"
-- This sounds like MUSCLE was used to select the orthogroups, which doesn't make sense. This should be clarified.
Line 182: "alignment by an in-house Perl script."
-- This script should be made publicly available either as a text file in the supplement or on a code repository like GitHub.

Line 182: " maximum likelihood phylogeny was reconstructed using RAxML"
-- There is no information regarding model, partitioning, etc. If partitioning was used, the partition file should be supplied. The alignment should be supplied as a textfile. Output treefiles should be made available as text files in the supplement.
Line 183: "The results supported the view that R. esculentum and H. vulgaris are sister groups."
-- This is wrong. Figure 4 has A. aurita as sister to R. esculentum
Line 189: "27 and 27 gene families"
-- typo?
Line 200: "244 unique gene families that could be annotated in NR"
-- How could they be annotated? What software? What cutoff?
Line 202: "were annotated with the proteins of the species in Anthozoa"
-- How were they annotated?
Line 203: "This was also supported by the results of phylogenetic analysis among the 13 species"
-- what was supported. I don't understand.
Line 204: "Compared to H. vulgaris and Aurelia, R. esculentum had fewer gene gains (331) and fewer gene losses (294)."
-- Sentence should include the number of gains and losses in H. vulgaris and Aurelia.
Line 206: "were kept by Anthozoa species and R. esculentum, but were lost in other Scyphozoa species."
-- the entire quote above would be clearer and more accurate if replaced by "were lost in Aurelia and H. vulgaris."
Line 208: "To further explore this hypothesis..."
-- What hypothesis? There has not been one stated.
Line 208: "There were 7542, 7864, 7611 and 6141 orthogroups found in Re-Hv, Aa-Hv, Nn-Hv and Cx-Hv, 10213 respectively."
-- How were paralogs treated? How were sequences aligned? This very rough positive selection analysis is not convincing. I don't trust these results to tell anything (even though it is unclear what the results are supposed to say)
Line 219: "Jellyfish, one of the main subgroups of Cnidaria, is one of the oldest extant lineages of venomous animals"
-- This sentence implies that Jellyfish are older than other cnidarian groups. I think the authors were trying to say that cnidarians are older than other venomous animals. However, even this is problematic since the lineage leading to snakes or cone snails (for example) are as old as the lineage leading to cnidarians assuming we are measuring the age from the ancestor (e.g. from the last common ancestor of all animals). This sentence could be deleted.
Line 235: "Second, according to the gene annotations of NR, Uniprot and Tox-Prot, the genes annotated consistently were chosen as the toxin-like genes."
-- This sentence is not clear. Active voice may help. Also, if there is explicit criteria being used, it should be stated clearly.
Line 237: "Third, to make the pool of venom-related genes more complete, we screened all the genes predicted in the genome manually by their annotation."
-- This too is unclear. Also, manual curation is difficult to repeat, but if it must be manual, than detailed criteria should be supplied. It would be much better to convert the manual curation criteria into a

program and provide this program as supplemental material. Either way, it needs to be repeatable.
Line 253: "were observed in the tentacles of scyphozoan and cubozoan species "
-- Assuming these observations were not made as part of this study, it would be more clear to say "have been observed in the tentacles of scyphozoan and cubozoan species"
Line 256" "suggesting their important roles during evolution."
-- It is not clear why this result suggests "important roles during evolution." This should be clarified or removed.
Line 259: "were highly abundant in cubozoan venoms"
-- Assuming these observations were not made as part of this study, it would be more clear to say "have been observed in high abundance in cubozoan venoms"
Line 259: "were also reported"
-- "have been reported"
Line 265: "Three new toxin-like genes that have not been previously reported in jellyfish were..."
-- Prothrombin was reported in Cassiopea in Ohdera et al. 2019 (Table S4 -- line 73).
Line 274: "was mainly identified in the honeybee"
-- grammar issue makes this confusing.
Line 275: "The new toxin-like genes identified in this study will provide insight into the complex composition of jellyfish venom."
-- as written this sentence is kind of a throwaway. It would be better to mention that: the discovery of these toxin-coding genes in R. esculenum adds to a growing understanding of the composition of jellyfish venoms.
Line 281: "The different composition of venom may account for the different symptoms after sting."
-- what are the different symptoms?
Line 291: "It was also reported that neighboring genes tend to be co-expressed rather than expressed by chance"
-- This is misleading. This is an Arabidopsis paper. At least find a paper that shows this in animals and mention explicitly that in animals sometimes proximity can relate to co-expression.
Line 294: "Further studies are needed to clarify its specific functions."
-- This is not a great way to end the paper. Why not add a paragraph conclusion saying how these new data can be used in the future. etc. You could find many examples from other giganotes.
Table 3: "The green and yellow boxes indicate the identified and unidentified toxin-like genes respectively"
-- "unidentified toxin-like genes" is confusing. It would be more clear to make yellow boxes white (or unfilled) and state that "green boxes represent potential toxin-coding genes."
Figure 1: "Picture of a jellyfish R. esculentum captured from Yingkou, Liaoning Province, China."
-- "Photograph of Rhopilema esculentum collected from Yingkou, Liaoning Province, China." Either include approximate size or a scale bar.
-- Also, Figure 1 is not (but should be) referenced in the main text.

Level of Interest

Please indicate how interesting you found the manuscript: Choose an item.

Quality of Written English

Please indicate the quality of language in the manuscript: Choose an item.

Declaration of Competing Interests

Please complete a declaration of competing interests, considering the following questions:

- Have you in the past five years received reimbursements, fees, funding, or salary from an organisation that may in any way gain or lose financially from the publication of this manuscript, either now or in the future?
- Do you hold any stocks or shares in an organisation that may in any way gain or lose financially from the publication of this manuscript, either now or in the future?
- Do you hold or are you currently applying for any patents relating to the content of the manuscript?
- Have you received reimbursements, fees, funding, or salary from an organization that holds or has applied for patents relating to the content of the manuscript?
- Do you have any other financial competing interests?
- Do you have any non-financial competing interests in relation to this paper?

If you can answer no to all of the above, write 'I declare that I have no competing interests' below. If your reply is yes to any, please give details below.

I declare that I have no competing interests

I agree to the open peer review policy of the journal. I understand that my name will be included on my report to the authors and, if the manuscript is accepted for publication, my named report including any attachments I upload will be posted on the website along with the authors' responses. I agree for my report to be made available under an Open Access Creative Commons CC-BY license (http://creativecommons.org/licenses/by/4.0/). I understand that any comments which I do not wish to be included in my named report can be included as confidential comments to the editors, which will not be published.

Choose an item.

To further support our reviewers, we have joined with Publons, where you can gain additional credit to further highlight your hard work (see: https://publons.com/journal/530/gigascience). On publication of this paper, your review will be automatically added to Publons, you can then choose whether or not to claim your Publons credit. I understand this statement.

Yes Choose an item.
