# Supplementary material for: Chromosome-level reference genome of the jellyfish Rhopilema esculentum
Source: Gigascience. 2020 Apr 21;9(4):giaa036. doi: 10.1093/gigascience/giaa036 (PMC7172023; doi:10.1093/gigascience/giaa036)
Supplement: giaa036_Reviewer_2_Report_Original_Submission — Lucas LeclÃ¨re -- 11/10/2019 Reviewed [file giaa036_reviewer_2_report_original_submission.pdf]

Reviewer Report

Title: Chromosome-level reference genome of the jellyfish Rhopilema esculentum

Version: Original Submission **Date:** 11/10/2019

Reviewer name: Lucas LeclÃ¨re

Reviewer Comments to Author:

This manuscript presents a chromosome-level genome assembly of the edible scyphozoan jellyfish Rhopilema esculentum, combining Illumina, PacBio and HiC technologies. As there is currently no published chromosome-level cnidarian genome assembly, this study represents certainly a very interesting resource for future studies.

Major comments:

Several relevant methodological details are missing, and would need to be provided:

- The origin of the biological material from which the DNA and RNA samples were prepared is not specified. Did they derive from wild animals or cultured animals? Which geographical origin and/or which strain(s)? How were the animals collected? Were the samples pooled, and if so, how many individuals/piece of tissues were used for each extraction?
- Which procedures were performed in order to avoid contamination? Were the organisms starved prior to extraction? Were they treated with antibiotics?
- For RNAseq experiments - please provide details about the sequencing. Was it pair-ended? How many replicates? This information should be provided in the main text.
- The phylogenetic methods used to produce figure 4 should be provided. Which model was used for the RaXML analyses? How was done the fossil calibration? Which method was used to calibrate the tree?

All the scripts mentioned in the main text should be provided as supplementary information (e.g. L102, L182).

The statement that R. esculentum evolved less than other scyphozoan species is not very convincing. This would need to be studied in deeper detail with a greater number of compared species. I would suggest removing this statement from both main text and abstract. The lower number of gene gain and loss detected in R. esculentum, compared to other schyphozoans, relies on the comparison of too few species. I would also suggest entirely removing the part on positive selection in R. esculentum (L208-L216), since the species used for comparison (Aurelia aurita and Hydra vulgaris) are too distant phylogenetically for an accurate estimate of the selective forces.

Other comments:

I would suggest removing the parentheses in the title.

L17-18: The phylogenetic position of ctenophore is still highly debated. I would suggest removing "after their divergence with Ctenophora". Same comment for L40-41.

L19: add 'an': "is an important".

L23-24. The sentence "A total… respectively." seems to me unnecessary for an abstract. I would suggest removing it.

L32: replace "we first finished" with "we generated a"
L41: add the year of species description "1891" after "Kishinouye".
L42: lowercase for "Class" - also L198.
L47: rephrase "their harmfulness to industry and the community in blooms".
L48: replace "for" with "because of" and provide a reference in support of that claim.
L54: replace "Aurelia and Aurelia aurita" with "Aurelia aurita".
L53-56. Other recent releases of genome assembly of non-scyphozoan medusozoans - cubozoan (Morbakka virulenta, and Alatina alata), hydrozoan (Clytia hemisphaerica), staurozoan (Calvadosia cruxmelitensis) could also be mentioned here.
L56. I would delete "mainly concerning the increasing jellyfish blooms throughout the world" as the recent publications of scyphozoan genome were only marginally addressing this point.
Rhopilema esculentum is often referenced in the text simply as "jellyfish". To avoid confusion, I would replace "jellyfish" with "R. esculentum" when appropriate. e.g.: L148, L161, L164.
L151. "were performed essentially as previously described [28]." Please detail the difference with the published protocol, if any.
L198. Replace "Aurelia" with "A. aurita".
L219. Replace "Jellyfish" with "Medusozoa".
L236. "the genes annotated… toxin-like genes". This part of the sentence is unclear, please reformulate.
L258. "Two copies… found". This sentence is unclear, please reformulate.
L281. "They are known to…. neurotoxins". A reference needs to be added here.
L283. "Some" - how many?
L285. The term "associated" is too imprecise and should be replaced. Do you mean: found on the same scaffold? If so, how distant?
L290. It is not true that "genes located closely in genome are always involved in related functions and expressed in similar patterns". Please correct or delete the sentence.
L292. I would avoid the passive mode and replace "It was suggested" with "We suggest".
L294. Remove "the". "and function of jellyfish" is unclear - reformulate or delete. Please also replace "its" with "their".
Figure 2F: How were calculated those "major interchromosomal relationships"? How to interpret them? Please also provide scale bars for A,C,D,E.
Figure 3. Please describe in the figure legend the meaning of the numbers and numbers between brackets shown on the figure.

Level of Interest

Please indicate how interesting you found the manuscript: Choose an item.

Quality of Written English

Please indicate the quality of language in the manuscript: Choose an item.

Declaration of Competing Interests

Please complete a declaration of competing interests, considering the following questions:

- Have you in the past five years received reimbursements, fees, funding, or salary from an organisation that may in any way gain or lose financially from the publication of this manuscript, either now or in the future?
- Do you hold any stocks or shares in an organisation that may in any way gain or lose financially from the publication of this manuscript, either now or in the future?
- Do you hold or are you currently applying for any patents relating to the content of the manuscript?
- Have you received reimbursements, fees, funding, or salary from an organization that holds or has applied for patents relating to the content of the manuscript?
- Do you have any other financial competing interests?
- Do you have any non-financial competing interests in relation to this paper?

If you can answer no to all of the above, write 'I declare that I have no competing interests' below. If your reply is yes to any, please give details below.

I declare that I have no competing interests.

I agree to the open peer review policy of the journal. I understand that my name will be included on my report to the authors and, if the manuscript is accepted for publication, my named report including any attachments I upload will be posted on the website along with the authors' responses. I agree for my report to be made available under an Open Access Creative Commons CC-BY license (http://creativecommons.org/licenses/by/4.0/). I understand that any comments which I do not wish to be included in my named report can be included as confidential comments to the editors, which will not be published.

Choose an item.

To further support our reviewers, we have joined with Publons, where you can gain additional credit to further highlight your hard work (see: https://publons.com/journal/530/gigascience). On publication of this paper, your review will be automatically added to Publons, you can then choose whether or not to claim your Publons credit. I understand this statement.

Yes Choose an item.
