# Supplementary material for: Chromosome-level reference genome of the jellyfish Rhopilema esculentum
Source: Gigascience. 2020 Apr 21;9(4):giaa036. doi: 10.1093/gigascience/giaa036 (PMC7172023; doi:10.1093/gigascience/giaa036)
Supplement: giaa036_Reviewer_3_Report_Original_Submission — David A. Gold -- 11/19/2019 Reviewed [file giaa036_reviewer_3_report_original_submission.pdf]

Reviewer Report

Title: Chromosome-level reference genome of the jellyfish Rhopilema esculentum

Version: Original Submission **Date:** 11/19/2019

Reviewer name: David A. Gold

Reviewer Comments to Author:

In this paper, Li et al. describe the genome of the jellyfish Rhopilema. This is the first chromosome-level assembly of a scyphozoan (true jellyfish), and the data looks of wonderful quality. This paper is worthy of publication in Gigascience, but there are a couple of additional analyses and minor edits that need to be addressed first:

1) A small (but important) point. In several places the authors discuss the Rhopilema genome as being "less evolved" than other scyphozoans (e.g. lines 29-30, 216). All organisms demonstrate lineage-specific patterns of change, and in that regard all species are equally "evolved". The term the authors should be using is "less derived".

2) The authors do not include any comparative macro- or microsynteny analyses, which is standard in a genome paper. Given the large number of cnidarian genomes and the quality of their assembly, it would be valuable to see how genomic structure in Rhopilema compares to other cnidarians. I would especially like to see whether synteny analysis supports the less derived nature of the Rhopilema genome, as gene gain/loss analysis appears to.

3) There is no description on how the authors dated their molecular clock for Figure 4, other than they used "the phylogeny and fossil records" (line 184). The dates near the base of the tree are outside of the range of most molecular clock analyses (see refs. cited below for detail), which makes the subsequent CAFE analysis suspect. The authors need to provide more information on how their tree is dated and consider methodologies that bring their clock closer in line with more thorough analyses.

4) This last suggestion is optional. The authors note a set of venom genes lie close together in the genome and posit that they may be co-expressed. This seems readily testable using the RNA-Seq data that they collected from the different life stages (Table S3). I'm generally confused why the authors collected so much RNA-Seq data (which, as far as I can tell, looks to be of high quality) and only use it for gene modelling. The paper could be much more compelling if that data were leveraged to understand how gene expression changes through the life cycle. But perhaps the authors are saving that for a subsequent paper?

Overall this is a well-written manuscript with a quality dataset. I look forward to seeing this paper published once some additional, minor analyses are done.

References:

Dohrmann M, WÃ¶rheide G. 2017. Dating early animal evolution using phylogenomic data. Sci Rep 7:3599.

dos Reis M, Thawornwattana Y, Angelis K, Telford MJ, Donoghue PC, Yang Z. 2015. Uncertainty in the timing of origin of animals and the limits of precision in molecular timescales. Curr Biol 25:2939-2950.

Level of Interest

Please indicate how interesting you found the manuscript: Choose an item.

Quality of Written English

Please indicate the quality of language in the manuscript: Choose an item.

Declaration of Competing Interests

Please complete a declaration of competing interests, considering the following questions:

- Have you in the past five years received reimbursements, fees, funding, or salary from an organisation that may in any way gain or lose financially from the publication of this manuscript, either now or in the future?
- Do you hold any stocks or shares in an organisation that may in any way gain or lose financially from the publication of this manuscript, either now or in the future?
- Do you hold or are you currently applying for any patents relating to the content of the manuscript?
- Have you received reimbursements, fees, funding, or salary from an organization that holds or has applied for patents relating to the content of the manuscript?
- Do you have any other financial competing interests?
- Do you have any non-financial competing interests in relation to this paper?

If you can answer no to all of the above, write 'I declare that I have no competing interests' below. If your reply is yes to any, please give details below.

I declare that I have no competing interests

I agree to the open peer review policy of the journal. I understand that my name will be included on my report to the authors and, if the manuscript is accepted for publication, my named report including any attachments I upload will be posted on the website along with the authors' responses. I agree for my report to be made available under an Open Access Creative Commons CC-BY license (http://creativecommons.org/licenses/by/4.0/). I understand that any comments which I do not wish to be included in my named report can be included as confidential comments to the editors, which will not be published.

Choose an item.

To further support our reviewers, we have joined with Publons, where you can gain additional credit to further highlight your hard work (see: https://publons.com/journal/530/gigascience). On publication of

this paper, your review will be automatically added to Publons, you can then choose whether or not to claim your Publons credit. I understand this statement.

Yes Choose an item.
